# ^1^H NMR-Based Framework for Estimating Molecular Formation Enthalpies of Organic Compounds

**DOI:** 10.1021/acs.joc.6c01225

**Published:** 2026-08-02

**Authors:** Daniel Konnikov, Shira D. Shamis, Keren Iudanov, Steven C. Zimmerman, N. Gabriel Lemcoff

**Affiliations:** † Department of Chemistry, 26732 Ben-Gurion University of the Negev, Beer-Sheva, 84105, Israel; ‡ Department of Chemistry, University of Illinois at Urbana−Champaign, Urbana, Illinois 61801, United States

## Abstract

We report a method for estimating standard enthalpies of formation (ΔHf°) directly from routine ^1^H NMR spectra. By combining spectral features with heteroatom-based functional motifs, accurate models were obtained (R^2^ ≈ 0.99; RMSE ≈ 5.5 kcal mol^–1^). The predictive performance was consistent across multiple validation approaches and comparable to established group contribution methods, while avoiding the need for knowledge of compound structure. The obtained formation enthalpies further enabled reasonable estimation of reaction enthalpies for representative organic transformations. Overall, the results demonstrate that routinely accessible NMR observables capture trends associated with molecular thermodynamic stability.

## Graphical abstract

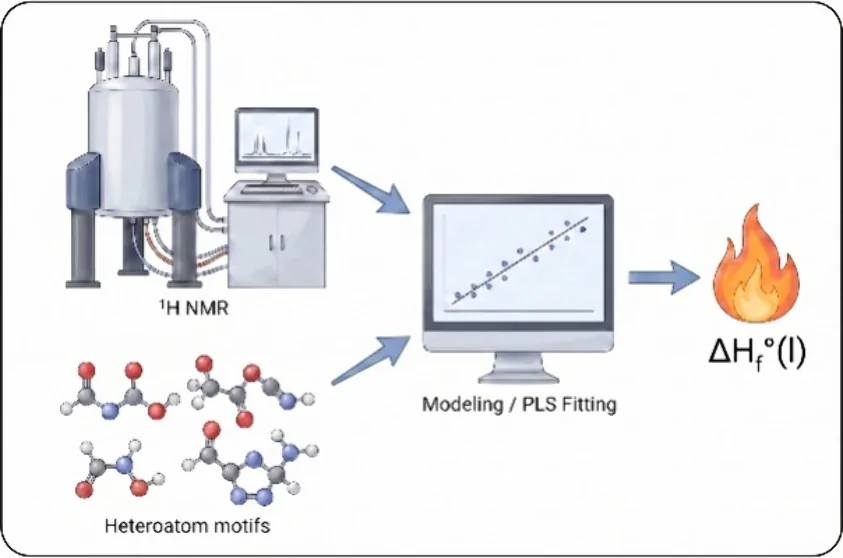

The standard enthalpy of formation (ΔHf°) is a fundamental thermodynamic property of organic compounds and an important descriptor of molecular stability and reactivity. However, its experimental determination remains challenging. Direct measurements, such as bomb calorimetry, are reliable but require complete consumption of the sample.^1^ Additionally, modern thermochemical reference data have been significantly improved through the development of the Active Thermochemical Tables (ATcT), which integrate experimental measurements and high-level theoretical calculations within a self-consistent thermochemical network.^2^ Computational approaches offer accurate thermochemical predictions and have become indispensable tools in this field. Nevertheless, they typically require detailed structural information and methodological expertise.^3^ Simpler methods, such as group contribution approaches, reduce computational cost but still depend on explicit atom connectivity and predefined structural fragments.^4^ Consequently, experimentally accessible approaches capable of estimating formation enthalpies with minimal structural input remain of considerable interest.

Proton nuclear magnetic resonance (^1^H NMR) spectroscopy is one of the most routine and information-rich techniques in organic chemistry, providing highly reproducible chemical shift data across instruments.^5^ In addition to structural elucidation, ^1^H NMR spectra reflect functional group composition and the local electronic environment surrounding hydrogen atoms.^6–9^ Because the enthalpy of formation originates from the energetic contributions of chemical bonding and electronic structure,^10–12^ it is reasonable to expect that thermodynamically relevant information may also be embedded within NMR observables.

The possibility that NMR observables correlate with energetic properties has long been recognized, although relatively few general approaches have been developed. Existing studies are often restricted to specific molecular classes or rely on isolated descriptors combined with additional information.^13–15^ At the same time, analyses of other spectroscopic techniques, such as IR spectroscopy, have demonstrated that complex physicochemical properties can be inferred from spectral features.^16,17^ These precedents suggest that spectral fingerprints may contain broader quantitative chemical information than what is typically extracted through conventional interpretation.

In this study, we investigate whether routinely acquired ^1^H NMR spectral information can be quantitatively related to standard enthalpies of formation across a chemically diverse data set of 364 organic molecules and analyze its predictive power.

## Results and Discussion

In conventional group additivity approaches (Figure 1B), the enthalpy of formation is estimated by summing atom-centered energetic increments derived from structural decomposition. In contrast, the present approach uses experimentally observed spectral information directly (Figure 1A). Molecular features are generated from the ^1^H NMR spectrum by assigning signals to chemical shift bins and summing their corresponding proton counts. This representation reflects the distribution of hydrogen environments throughout the molecule and enables extraction of chemically relevant information without the need for explicit structural decomposition.

**Figure 1:**
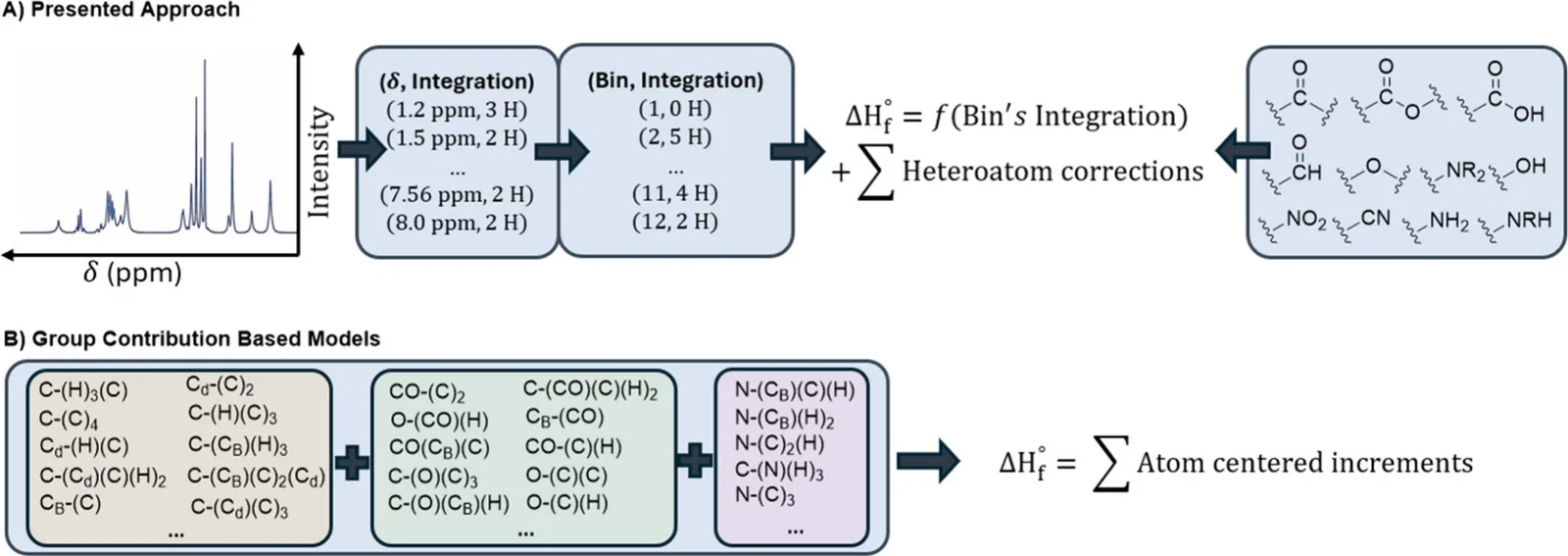
Conceptual framework of the hybrid NMR-based model compared with traditional group additivity approaches.

For hydrocarbons, these spectral descriptors capture thermochemical trends remarkably effectively. However, predictive performance decreases in the presence of heteroatom-containing functional groups. This behavior likely reflects a fundamental thermodynamic limitation: whereas the standard enthalpy of formation depends strongly on elemental composition, proton-based spectral information alone does not uniquely define heteroatom identity or abundance. To address this case, a limited set of functional motif corrections associated with heteroatom-containing groups was introduced to capture thermochemical contributions not fully reflected by proton environments alone. Together, these parameters enable accurate estimation of formation enthalpies.

Several spectral representations were evaluated, with the integration sum vector (ISV), defined as the total proton integration within each chemical shift bin, providing the best balance between predictive performance and simplicity (Table S4). The final model therefore employs ISV descriptors together with a limited number of heteroatom corrections.

The relationship between spectral features and formation enthalpy was modeled using partial least-squares (PLS) regression, a method well suited for correlated spectroscopic variables.^18,19^ The number of latent components was selected through inner cross-validation using the one-standard-error criterion. The influence of bin width and spectral range was additionally examined (Tables S5–S9). Comparable performance was obtained across multiple binning schemes, although a bin width of 0.75 ppm provided a favorable balance between predictive accuracy and model simplicity. The spectral region was restricted to 0–10.5 ppm, simply because inclusion of higher chemical shifts did not improve performance and introduced less reproducible resonances associated primarily with carboxylic acid O–H signals.

Model performance was evaluated using repeated nested 10-fold cross-validation (30 repetitions) together with repeated 75/25 hold-out validation (30 random splits). All parameter optimization procedures were confined to the training data in each iteration to avoid information leakage. Predictor standardization was likewise derived exclusively from training-set statistics before application to validation data. Additional implementation details are provided in the Supporting Information.

Model I is based solely on ISV (^1^H NMR integration) descriptors and includes 138 hydrocarbon molecules (Figure 2B). Model II expands the chemical space by incorporating 161 oxygen-containing compounds, whereas Model III further extends it to 65 nitrogen-containing species, resulting in a total data set of 364 molecules. With increasing chemical complexity, Models II and III additionally introduce heteroatom motif count descriptors to account for compositional effects not captured by spectral integration alone.

**Figure 2:**
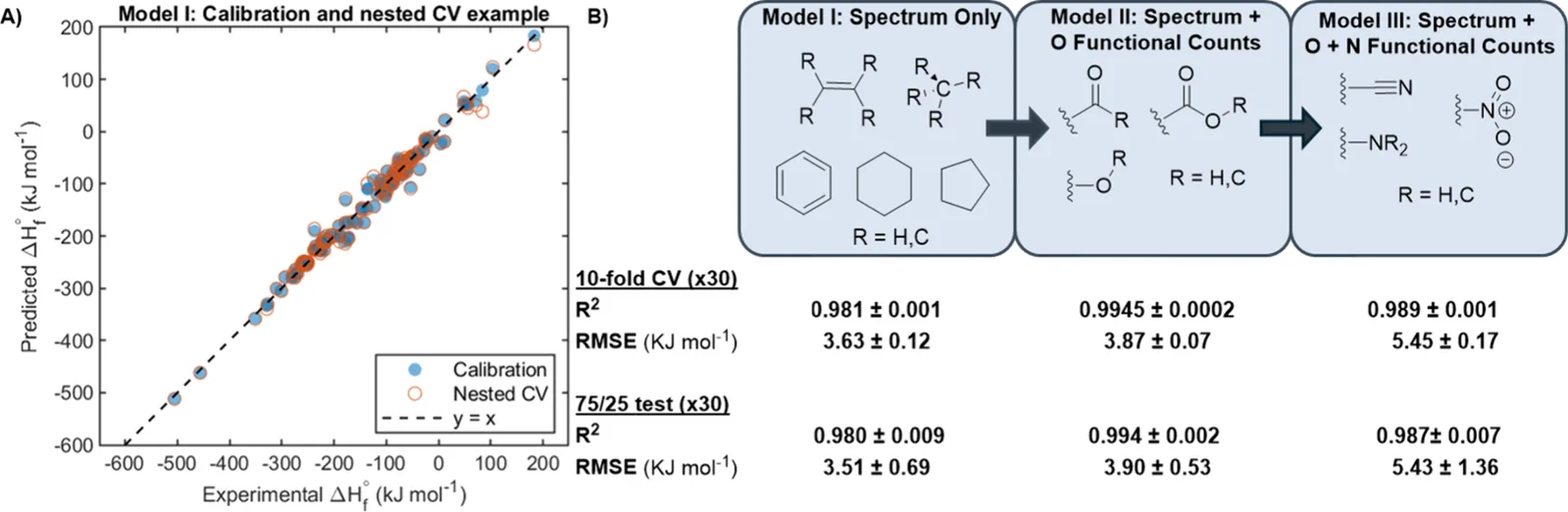
A) parity plot for model I (hydrocarbons;^1^H NMR descriptors), training and nested cross-validation (CV) example predictions vs experimental values. B) chemical progression from Model I to III and accuracy metrics for each model.

Figure 2A presents the parity plot for Model I, including both calibration results and a representative nested 10-fold cross-validation. Within the hydrocarbon domain, the model captures variation in standard enthalpy of formation effectively, consistent with changes in proton density distribution along the chemical shift axis. The data set spans aliphatic, olefins, aromatic, and cyclic hydrocarbons, including mixed structural motifs such as cycloalkyl–aryl frameworks.

However, when extending the chemical space to oxygen- and nitrogen-containing compounds, predictive performance deteriorates if only spectral integration descriptors are used (Figures S8 & S10). Although the overall linear relationship remains largely preserved, systematic offsets emerge between functional group classes, indicating that spectral descriptors alone do not fully encode compositional effects. As aforesaid, heteroatom motif count descriptors were introduced, providing minimal but effective compositional correction without introducing connectivity or positional information.

Figure 2 summarizes model performance across repeated nested 10-fold cross-validation and repeated 75/25 hold-out validation. The strong agreement between these two schemes across all models indicates stable generalization behavior and no evidence of overfitting. The observed increase in prediction error from Model I to Model III is therefore not indicative of reduced model robustness, but rather reflects increasing chemical diversity and reduced descriptor-space density as the domain expands.

To further exclude chance correlation, permutation testing was performed. In Y-scrambling, the target vector (ΔH_f_°) was randomly permuted while the descriptor matrix remained fixed, and the full validation pipeline was repeated. Under randomization, predictive performance collapsed to near-zero R^2^ values, with substantially increased errors. Across 500 permutations, an empirical P-value of approximately 0.002 was obtained (Figures S11–S13), confirming that the learned relationships are statistically significant and not artifacts of random correlation.

Following validation, the framework was benchmarked against a classical group contribution approach using the Benson increment method. From the full data set of 364 compounds, a random subset of 42 molecules that fell within the applicability domain of the Benson group additivity scheme and for which experimental liquid-phase vaporization enthalpies were available was selected, and their standard liquid-phase enthalpies of formation were estimated using the Benson scheme (see Supporting Information). Molecular structures are decomposed into atom-centered fragments, each contributing tabulated energetic increments, which are summed to yield gas-phase formation enthalpies.^20^ These were subsequently converted to liquid-phase values using experimental enthalpies of vaporization to ensure consistency.

On this subset, the proposed model achieves performance comparable to the Benson approach (Benson R^2^ = 0.9955; Model cross-validation R^2^ = 0.9925, Figure S14). Importantly, this comparison is notable because the Benson method relies on experimentally derived phase-correction terms, whereas the present approach predicts liquid-phase formation enthalpies directly from spectroscopic information without requiring auxiliary thermodynamic data.

To interpret the model structure, variable importance in projection (VIP) analysis was performed (Section S10). The results indicate that both descriptor blocks contribute meaningfully to the latent space, with comparable influence from ^1^H NMR-derived descriptors and heteroatom motif counts. When heteroatom descriptors are removed (Model III reduced form), the model still captures the overall thermochemical trend, but with reduced accuracy (Figure S10). This behavior is consistent with Model I, which demonstrates that spectral descriptors alone already encode a significant fraction of enthalpic variance.

Overall, these findings support the hypothesis that ^1^H NMR-derived spectral features encode substantial thermochemical information, while compositional descriptors provide essential corrections for heteroatom-containing systems.

A direct application of this framework is the estimation of reaction enthalpies. Because elemental composition is conserved in chemical reactions, reaction enthalpies can be obtained from differences in predicted formation enthalpies of products and reactants:

$$\Delta H_{rxn}=\sum H_{f}\left(products\right)-\sum H_{f}\left(reactants\right)$$

To demonstrate this, reaction enthalpies for six representative reactions were calculated using predicted formation enthalpies and compared with experimental values. To ensure a strict external validation setting, all molecules present in these reactions were excluded from training, resulting in a final model trained on 355 compounds. The resulting model exhibits an MAE of 3.37 kcal mol^–1^.

Table 1 summarizes the reaction set alongside experimental and predicted reaction enthalpies. In all cases, the model correctly reproduces both the sign and the order of magnitude of the reaction enthalpy. Larger deviations are observed for the hydrogenation of nitrobenzene to aniline (6). These discrepancies are likely influenced by the fact that reaction (6) was measured under nonstandard conditions (elevated temperature and pressure).

**Table 1:**
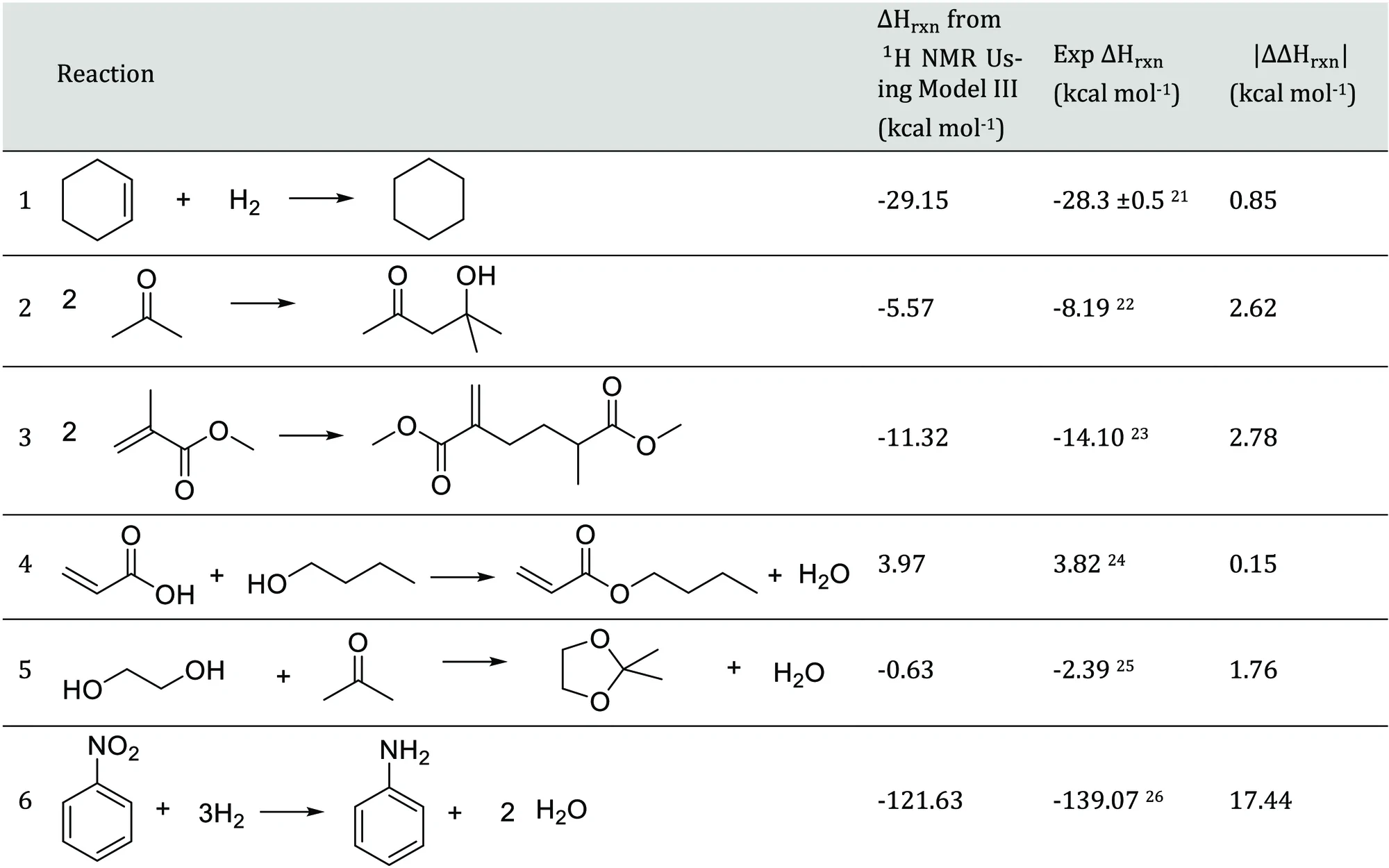
Calculated Reaction Enthalpies Estimated Using the Presented Model, the Experimental Values, and the Difference^21,22,23,24,25,26^

Overall, these results demonstrate that the model can be extended beyond molecular properties to provide reasonable estimates of reaction enthalpies for previously unseen systems within its applicability domain.

A key limitation of the present approach is the bounded applicability domain, which is constrained by the availability of reliable liquid-phase enthalpies of formation and corresponding ^1^H NMR spectra in CDCl_3_. Expanding this data set is nontrivial, as such paired experimental data are not uniformly available across chemical space.

In addition, whereas ^1^H NMR integration descriptors capture broad structural trends, they are inherently limited in resolving highly substituted or densely functionalized systems. Although moderately substituted aromatics and aliphatic frameworks are well represented and accurately predicted, more heavily substituted systems may not be fully distinguishable using proton environments alone. Future work will therefore focus on expanding chemical diversity, improving descriptor richness through complementary spectroscopic inputs, and increasing coverage of highly functionalized molecular systems.

In conclusion, this work demonstrates that routinely acquired ^1^H NMR spectra contain sufficient encoded information to enable estimation of thermochemical properties. By combining spectral integration descriptors for hydro

carbons with minimal heteroatom motif corrections, the model predicts liquid-phase enthalpies of formation without explicit structural enumeration. This establishes the feasibility of linking simple, routinely available spectroscopic measurements to thermochemical properties in a data-driven framework, offering a low-cost alternative to traditional experimental and computational approaches

## Supplementary Material



## Data Availability

The data underlying this study are available in the published article and its Supporting Information.

## Notes

Additional details on model development, validation procedures, benchmark analyses, variable importance in projection (VIP) analysis, NMR spectra, and data set information (PDF)
