# Supplementary material for: 1H NMR-Based Framework for Estimating Molecular Formation Enthalpies of Organic Compounds
Source: J Org Chem. 2026 Aug 2;91(39):13103–7. doi: 10.1021/acs.joc.6c01225 (PMC13640429; doi:10.1021/acs.joc.6c01225)
Supplement: Supplementary file 1 [file jo6c01225_si_001.pdf]

Supporting information

^{1}H NMR Based Framework for Estimating Molecular Formation Enthalpies of Organic Compounds

Daniel Konnikov,[a] Shira D. Shamis,[a] Keren Iudanov,[a] Steven C. Zimmerman,[a,b] and N. Gabriel Lemcoff*[a]

Table of contents:

**S1**	Materials and Methods	S2-S4
**S2**	Sources of Enthalpy and ^{1}H NMR Data	S5-S15
**S3**	Dataset Used in the Study	S16-S26
**S4**	Comparison of Descriptor Vector Combinations	S27
**S5**	Comparison of Analyzed NMR Area	S28
**S6**	Comparison of Bin Widths	S29-S31
**S7**	Correlation Plots for Individual Models	S32-S34
**S8**	Permutation Tests (Y-scrambling)	S35-S37
**S9**	Comparison to Benson	S38-S40
**S10**	Variable Importance in Projection (VIP) Plots	S41-S42
**S11**	Regression equation of the final PLS model	S43
**S12**	Reaction enthalpy calculation	S44-S45
**S13**	MATLAB Script for Data Analysis	S46-S53
**S14**	References	S54-S57

S1 - Materials and Methods :

All standard enthalpies of formation were obtained from the *CRC Handbook of Chemistry and Physics*, 106th Edition, Section 9: *Standard Thermodynamic Properties of Chemical Substances*.[1] For consistency, **only experimental values reported for the liquid phase** were used in this work. Because experimental liquid-phase enthalpies of formation are unavailable for several molecules of interest that are relevant to the dataset, additional compounds with reliable gas-phase enthalpies of formation and experimentally reported enthalpies of vaporization were included. Their corresponding liquid phase enthalpies were derived accordingly, as detailed in section S2, Table S2.

The ^{1}H NMR spectra used for correlation analysis were obtained from the Spectral Database for Organic Compounds (SDBS).[2] When SDBS data were unavailable or the corresponding spectrums were recorded in a solvent other than $CDCl_3$, the chemical shift values were verified against independent literature sources that reported spectra measured in $CDCl_3$. If no suitable data were available, ^{1}H NMR spectra was measured in our laboratory. All spectra were recorded in **deuterated chloroform ($CDCl_3$) at near ambient temperature conditions**.

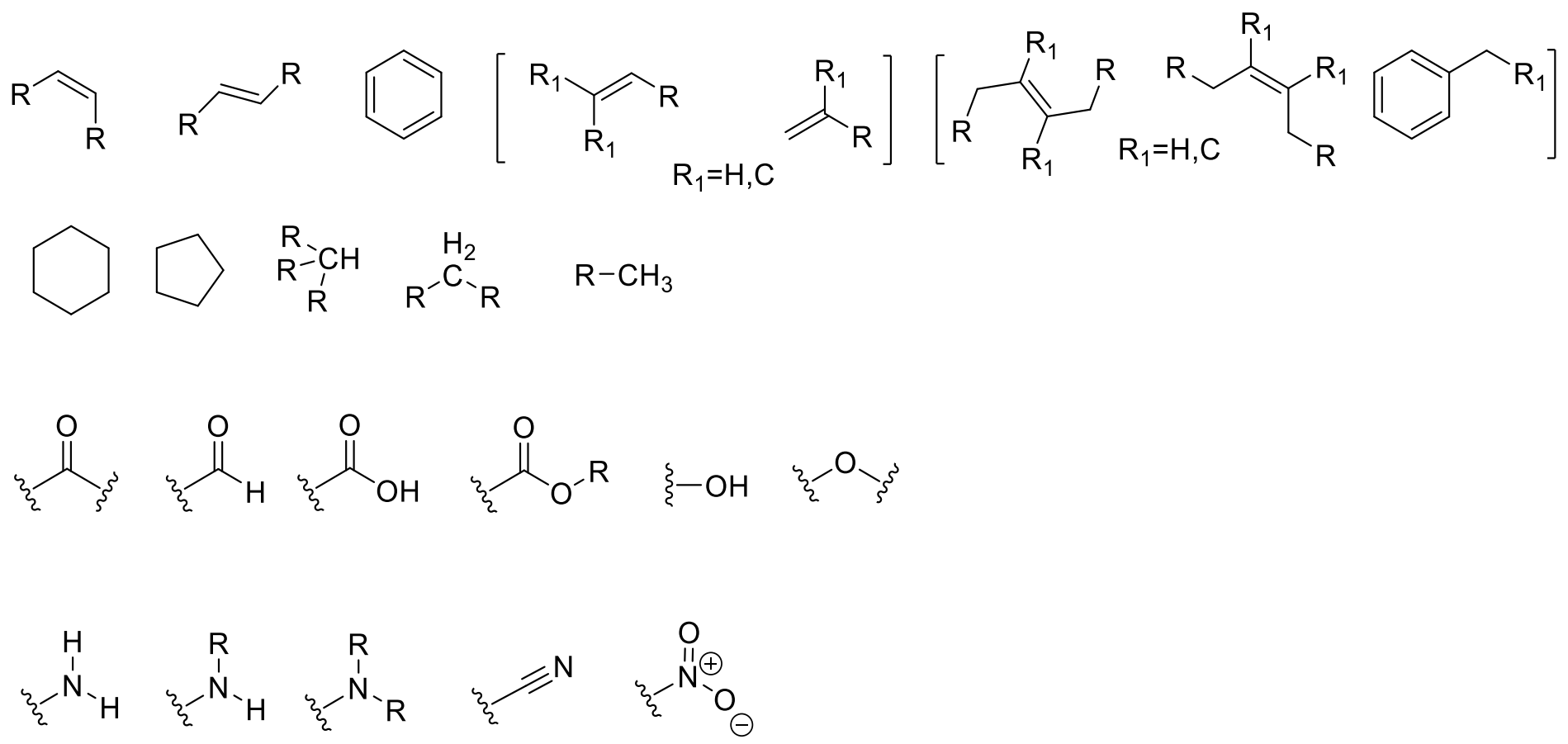

Figure S1: Groups that the molecules in the database are composed of.

The molecules included in the dataset are composed of a variety of structural and functional motifs. The groups present within the molecular population analyzed in this study are illustrated in Figure S1.

For each molecule, the dataset includes the ^{1}H NMR chemical shift values relative to reference standard, the corresponding signal integration values, and, for oxygen and nitrogen containing motifs, the associated existence descriptors (exists vector). The complete dataset is provided in Section S3.

The unparsed ^{1}H NMR data consisted of two columns: one containing the chemical shift values, corresponding to the position of the maximum of each signal, and a second column containing the integration values of the respective signals. For example, if a compound exhibited signals at *a*, *b*, and *c* ppm with corresponding integrations *A*, *B*, and *C*, the unparsed data would be represented as:

Shift Data: (a, b, c)
Integration Data: (A, B, C)

The selected ^{1}H NMR spectral region, defined from 0.0 ppm to *x* ppm, was divided into equal-width bins, producing *n* bins. The chemical shift and integration values were then assigned to bins according to the position of each signal. For example, if the bin width is defined as 0.5 ppm, bin 10 corresponds to the region between 4.5–5.0 ppm, and bin 14 corresponds to 6.5–7.0 ppm. If a compound exhibits the following chemical shift values *(4.75, 4.90, 6.8)* with corresponding integrations *(2, 1, 5)*, the signals are distributed according to their positions within the bins. In this case, bin 10 contains two shift values *(4.75, 4.90)* with integrations *(2, 1)*, while bin 14 contains one shift value *(6.8)* with integration *(5)*.This binning procedure was applied to all molecules in the dataset. Different bin widths and spectral regions analyzed were evaluated, and the corresponding results are presented in Section S5.

For each molecule, three base spectral descriptors were constructed in addition to the **exists descriptor**, which is present for molecules containing oxygen- or nitrogen-based functional motifs. The spectral descriptors were defined for each bin obtained from the previously described binning procedure. Consequently, if *n* bins were defined, each descriptor vector contains *n* elements. The descriptors are defined as follows.

1. **Weighted shift vector (WSV):** for each molecule m, the weighted shift value of bin v is defined as

$$W_{v,m} = \sum_{i} \delta_i * S_i$$

Where δ_i is the chemical shift value of signal i and S_i its absolute integration value. Each WGV therefore consists of *n* elements.

$$WSV_m = [W_1, W_2, \ldots, W_{n-1}, W_n]$$

2. **Integration sums vector (ISV):** for each molecule m, the total integrated intensity of bin v is defined as

$$I_{v,m} = \sum_{i} S_i$$

Giving another *n* elements vector:

$$ISV_m = [I_1, I_2, \ldots, I_{n-1}, I_n]$$

3. **Mean shift vector (MSV):** The mean chemical shift of bin v is obtained by dividing its weighted shift by its total integration:

$$M_{v,m} = \frac{W_{v,m}}{I_{v,m}}$$

Thus forming

$$MSV_m = [M_1, M_2, \ldots, M_{n-1}, M_n]$$

Although three spectral descriptors (WSV, ISV, and MSV) were initially defined, several descriptor combinations were evaluated during model development. The comparison of those is presented in Section S4. Among the tested variants, the most parsimonious and accurate model was obtained using the integration sums vector (ISV) descriptors combined with the exists motif descriptors. Consequently, the models discussed in the present work are based primarily on the ISV and Exists descriptors.

The obtained base descriptor vectors are highly collinear, as they originate from correlated spectroscopic features (e.g., integration values and chemical shift–weighted quantities). Consequently, applying conventional linear regression methods such as ordinary least squares (OLS), Ridge, or LASSO regression leads to parameter saturation relative to the dataset size, increasing the risk of overfitting and numerical instability. To address this issue, Partial Least Squares (PLS) regression was employed as the primary modeling technique. PLS constructs a reduced set of orthogonal latent variables that maximize the covariance between the predictor variables and the target property (the standard enthalpy of formation), thereby capturing the most relevant structural information while mitigating multicollinearity.

Prior to PLS analysis, predictor variables were mean-centered and scaled to unit variance, ensuring equal weighting across descriptors. Following standardization, descriptors encoding the absence of a feature (zero values) may take negative values, representing the absence of that feature relative to the average magnitude of the corresponding descriptor across the dataset. The number of latent PLS components was selected using nested 10-fold cross-validation within each training split. Model performance was evaluated using 30 repeated nested 10-fold cross-validation to assess statistical robustness, 30 repeated 75/25 hold-out validations to quantify generalization performance, and Y-scrambling tests to confirm that predictive performance arises from genuine structure–property relationships rather than chance correlations. Finally, the predictive performance of the developed model was compared with estimates obtained using the Benson group additivity method. Details regarding the Benson calculations are provided in Section S9.

All analyses were performed in MATLAB (R2025a) using custom scripts provided in Section S12. ChatGPT (OpenAI) was used to assist with code debugging. The TOC graphic was prepared using illustrae.

All calculations and coding were performed using units of $kJ \cdot mol^{-1}$. Reported values in the manuscript are converted to $kcal \cdot mol^{-1}$ for consistency with common chemical conventions. Unit conversion was performed using $1\ kcal \cdot mol^{-1} = 4.184\ kJ \cdot mol^{-1}$.

S2 - References to the H-NMR and Enthalpy data:

#	CAS RN	ΔH_f° source	$^{1}H-NMR$ source
1	496-11-7	CRC[1]	SDBS[2]
2	538-93-2	CRC[1]	SDBS[2]
3	97-85-8	CRC[1]	SDBS[2]
4	10588-10-0	CRC[1]	SDBS[2]
5	78-84-2	CRC[1]	Ref [3,4]
6	108-21-4	CRC[1]	SDBS[2]
7	75-31-0	CRC[1]	SDBS[2]
8	98-82-8	CRC[1]	SDBS[2]
9	527-84-4	CRC[1]	SDBS[2]
10	535-77-3	CRC[1]	Ref [4,5]
11	99-87-6	CRC[1]	Ref [6,7]
12	88-69-7	CRC[1]	SDBS[2]
13	618-45-1	CRC[1]	Ref [8,9]
14	67-56-1	CRC[1]	SDBS[2]
15	79-20-9	CRC[1]	SDBS[2]
16	95-53-4	CRC[1]	SDBS[2]
17	108-44-1	CRC[1]	SDBS[2]
18	78-78-4	CRC[1]	SDBS[2]
19	93-58-3	CRC[1]	SDBS[2]
20	78-79-5	CRC[1]	SDBS[2]
21	600-07-7	CRC[1]	Ref [10,11]
22	503-74-2	CRC[1]	SDBS[2]
23	34713-94-5	CRC[1]	SDBS[2]<br>Ref [12]
24	123-51-3	CRC[1]	SDBS[2]
25	70116-68-6	CRC[1]	Ref [13]
26	563-80-4	CRC[1]	SDBS[2]
27	563-46-2	CRC[1]	SDBS[2]
28	563-45-1	CRC[1]	Ref [14]
29	513-35-9	CRC[1]	SDBS[2]
30	1634-04-4	CRC[1]	SDBS[2]
31	811-93-8	CRC[1]	SDBS[2]
32	78-82-0	CRC[1]	SDBS[2]
33	78-83-1	CRC[1]	SDBS[2]
34	75-65-0	CRC[1]	SDBS[2]
35	557-17-5	CRC[1]	SDBS[2]
36	124-10-7	CRC[1]	SDBS[2]
37	1731-86-8	CRC[1]	SDBS[2]
38	871-83-0	CRC[1]	SDBS[2]
39	112-62-9	CRC[1]	SDBS[2]
40	1937-62-8	CRC[1]	SDBS[2]
41	111-11-5	CRC[1]	Ref [15,16]
42	7132-64-1	CRC[1]	SDBS[2]
43	107-83-5	CRC[1]	SDBS[2]
44	624-24-8	CRC[1]	SDBS[2]
45	565-67-3	CRC[1]	SDBS[2]

46	565-69-5	CRC[1]	SDBS[2]
47	646-04-8	CRC[1]	SDBS[2]
48	16529-66-1	CRC[1]	SDBS[2]
49	591-80-0	CRC[1]	SDBS[2]
50	107-10-8	CRC[1]	SDBS[2]
51	123-38-6	CRC[1]	SDBS[2]
52	110-89-4	CRC[1]	SDBS[2]
53	504-63-2	CRC[1]	SDBS[2]
54	107-12-0	CRC[1]	SDBS[2]
55	79-09-4	CRC[1]	SDBS[2]
56	71-23-8	CRC[1]	SDBS[2]
57	67-63-0	CRC[1]	SDBS[2]
58	109-66-0	CRC[1]	SDBS[2]
59	100-42-5	CRC[1]	SDBS[2]
60	1678-92-8	CRC[1]	SDBS[2]
61	141-06-0	CRC[1]	SDBS[2]
62	629-63-0	CRC[1]	SDBS[2]
63	1070-87-7	CRC[1]	SDBS[2]
64	16747-38-9	CRC[1]	SDBS[2]
65	112-60-7	CRC[1]	SDBS[2]
66	109-99-9	CRC[1]	SDBS[2]
67	119-64-2	CRC[1]	SDBS[2]
68	142-68-7	CRC[1]	SDBS[2]
69	542-28-9	CRC[1]	SDBS[2]
70	526-73-8	CRC[1]	SDBS[2]
71	108-67-8	CRC[1]	SDBS[2]
72	464-06-2	CRC[1]	SDBS[2]
73	594-56-9	CRC[1]	SDBS[2]
74	112-27-6	CRC[1]	SDBS[2]
75	102-86-3	CRC[1]	SDBS[2]
76	108-88-3	CRC[1]	SDBS[2]
77	3522-94-9	CRC[1]	SDBS[2]
78	540-84-1	CRC[1]	SDBS[2]
79	107-39-1	CRC[1]	SDBS[2]
80	107-40-4	CRC[1]	SDBS[2]
81	1116-76-3	CRC[1]	SDBS[2]
82	95-47-6	CRC[1]	SDBS[2]
83	108-38-3	CRC[1]	SDBS[2]
84	106-42-3	CRC[1]	SDBS[2]
85	1120-21-4	CRC[1]	SDBS[2]
86	75-52-5	CRC[1]	SDBS[2]
87	627-05-4	CRC[1]	SDBS[2]
88	79-24-3	CRC[1]	SDBS[2]
89	625-48-9	CRC[1]	SDBS[2]
90	628-05-7	CRC[1]	SDBS[2]
91	108-03-2	CRC[1]	SDBS[2]
92	638-04-0	CRC[1]	SDBS[2]
93	2146-38-5	CRC[1]	SDBS[2]

94	874-41-9	CRC[1]	SDBS[2]
95	1758-88-9	CRC[1]	SDBS[2]
96	16747-32-3	CRC[1]	SDBS[2]
97	1068-87-7	CRC[1]	SDBS[2]
98	3938-95-2	CRC[1]	SDBS[2]
99	609-26-7	CRC[1]	SDBS[2]
100	1067-08-9	CRC[1]	SDBS[2]
101	111-62-6	CRC[1]	SDBS[2]
102	617-78-7	CRC[1]	SDBS[2]
103	539-82-2	CRC[1]	SDBS[2]
104	90-00-6	CRC[1]	SDBS[2]
105	620-17-7	CRC[1]	SDBS[2]
106	619-99-8	CRC[1]	SDBS[2]
107	149-57-5	CRC[1]	SDBS[2]
108	104-76-7	CRC[1]	SDBS[2]
109	108-64-5	CRC[1]	SDBS[2]
110	107-21-1	CRC[1]	SDBS[2]
111	64-17-5	CRC[1]	SDBS[2]
112	103-73-1	CRC[1]	SDBS[2]
113	141-78-6	CRC[1]	SDBS[2]
114	112-41-4	CRC[1]	SDBS[2]
115	112-40-3	CRC[1]	SDBS[2]
116	142-84-7	CRC[1]	SDBS[2]
117	111-43-3	CRC[1]	SDBS[2]
118	109-92-2	CRC[1]	SDBS[2]
119	105-37-3	CRC[1]	SDBS[2]
120	628-32-0	CRC[1]	SDBS[2]
121	611-14-3	CRC[1]	SDBS[2]
122	620-14-4	CRC[1]	SDBS[2]
123	622-96-8	CRC[1]	SDBS[2]
124	111-71-7	CRC[1]	SDBS[2]
125	142-82-5	CRC[1]	SDBS[2]
126	629-08-3	CRC[1]	SDBS[2]
127	592-76-7	CRC[1]	SDBS[2]
128	14686-13-6	CRC[1]	SDBS[2]
129	14686-14-7	CRC[1]	SDBS[2]
130	544-76-3	CRC[1]	SDBS[2]
131	629-73-2	CRC[1]	SDBS[2]
132	592-42-7	CRC[1]	SDBS[2]
133	111-69-3	CRC[1]	SDBS[2]
134	110-54-3	CRC[1]	SDBS[2]
135	142-62-1	CRC[1]	SDBS[2]
136	111-27-3	CRC[1]	SDBS[2]
137	589-38-8	CRC[1]	SDBS[2]
138	592-41-6	CRC[1]	SDBS[2]
139	763-29-1	CRC[1]	SDBS[2]
140	691-37-2	CRC[1]	SDBS[2]
141	625-27-4	CRC[1]	SDBS[2]

142	691-38-3	CRC[1]	SDBS[2]
143	592-27-8	CRC[1]	SDBS[2]
144	589-53-7	CRC[1]	SDBS[2]<br>Ref [4]
145	106-73-0	CRC[1]	SDBS[2]
146	591-76-4	CRC[1]	SDBS[2]
147	106-70-7	CRC[1]	SDBS[2]
148	3524-73-0	CRC[1]	SDBS[2]
149	90-12-0	CRC[1]	SDBS[2]
150	1120-97-4	CRC[1]	SDBS[2]
151	497-26-7	CRC[1]	SDBS[2]<br>Ref [17]
152	1192-37-6	CRC[1]	SDBS[2]
153	108-87-2	CRC[1]	SDBS[2]
154	7731-28-4	CRC[1]	SDBS[2]
155	7731-29-5	CRC[1]	SDBS[2]
156	96-37-7	CRC[1]	SDBS[2]
157	25144-05-2	CRC[1]	SDBS[2]
158	1120-72-5	CRC[1]	SDBS[2]
159	693-89-0	CRC[1]	SDBS[2]
160	110-42-9	CRC[1]	SDBS[2]
161	598-98-1	CRC[1]	SDBS[2]
162	594-70-7	CRC[1]	SDBS[2]
163	112-05-0	CRC[1]	SDBS[2]
164	143-08-8	CRC[1]	SDBS[2]
165	821-55-6	CRC[1]	SDBS[2]
166	111-84-2	CRC[1]	SDBS[2]
167	111-65-9	CRC[1]	SDBS[2]
168	124-12-9	CRC[1]	SDBS[2]
169	111-87-5	CRC[1]	SDBS[2]
170	111-66-0	CRC[1]	SDBS[2]
171	7642-04-8	CRC[1]	SDBS[2]
172	13389-42-9	CRC[1]	SDBS[2]
173	110-62-3	CRC[1]	SDBS[2]
174	111-29-5	CRC[1]	SDBS[2]
175	110-59-8	CRC[1]	SDBS[2]
176	109-52-4	CRC[1]	SDBS[2]
177	71-41-0	CRC[1]	SDBS[2]
178	6032-29-7	CRC[1]	SDBS[2]
179	107-87-9	CRC[1]	SDBS[2]
180	96-22-0	CRC[1]	SDBS[2]
181	109-67-1	CRC[1]	SDBS[2]
182	627-20-3	CRC[1]	SDBS[2]
183	558-37-2	CRC[1]	SDBS[2]
184	2207-03-6	CRC[1]	SDBS[2]
185	2207-04-7	CRC[1]	SDBS[2]
186	108-01-0	CRC[1]	SDBS[2]<br>Figure S2

187	111-92-2	CRC[1]	SDBS[2]
188	84-74-2	CRC[1]	SDBS[2]
189	111-46-6	CRC[1]	SDBS[2]
190	60-29-7	CRC[1]	SDBS[2]
191	95-92-1	CRC[1]	SDBS[2]
192	84-66-2	CRC[1]	SDBS[2]
193	105-57-7	CRC[1]	SDBS[2]
194	629-14-1	CRC[1]	SDBS[2]
195	462-95-3	CRC[1]	SDBS[2]
196	100-37-8	CRC[1]	SDBS[2]
197	135-01-3	CRC[1]	SDBS[2]
198	141-93-5	CRC[1]	SDBS[2]
199	105-05-5	CRC[1]	SDBS[2]
200	590-35-2	CRC[1]	SDBS[2]
201	565-59-3	CRC[1]	SDBS[2]
202	108-08-7	CRC[1]	SDBS[2]
203	562-49-2	CRC[1]	SDBS[2]
204	565-80-0	CRC[1]	SDBS[2]
205	2213-32-3	CRC[1]	SDBS[2]
206	762-62-9	CRC[1]	SDBS[2]
207	625-65-0	CRC[1]	SDBS[2]
208	108-83-8	CRC[1]	SDBS[2]
209	590-73-8	CRC[1]	SDBS[2]
210	584-94-1	CRC[1]	SDBS[2]
211	589-43-5	CRC[1]	SDBS[2]
212	592-13-2	CRC[1]	SDBS[2]<br>Ref [4]
213	110-96-3	CRC[1]	SDBS[2]
214	108-18-9	CRC[1]	SDBS[2]
215	108-20-3	CRC[1]	SDBS[2]
216	91-16-7	CRC[1]	SDBS[2]
217	110-71-4	CRC[1]	SDBS[2]
218	109-87-5	CRC[1]	SDBS[2]
219	4744-10-9	CRC[1]	SDBS[2]
220	124-07-2	CRC[1]	SDBS[2]
221	534-15-6	CRC[1]	SDBS[2]
222	505-22-6	CRC[1]	SDBS[2]
223	123-91-1	CRC[1]	SDBS[2]
224	646-06-0	CRC[1]	SDBS[2]
225	75-84-3	CRC[1]	SDBS[2]
226	103-69-5	CRC[1]	SDBS[2]
227	100-41-4	CRC[1]	SDBS[2]
228	760-21-4	CRC[1]	SDBS[2]
229	1678-91-7	CRC[1]	SDBS[2]
230	1640-89-7	CRC[1]	SDBS[2]
231	544-63-8	CRC[1]	Ref [18,19]
232	4050-45-7	CRC[1]	SDBS[2]
233	7642-09-3	CRC[1]	SDBS[2]

234	107-18-6	CRC[1]	SDBS[2]
235	64-19-7	CRC[1]	SDBS[2]
236	67-64-1	CRC[1]	SDBS[2]
237	75-05-8	CRC[1]	SDBS[2]
238	98-86-2	CRC[1]	SDBS[2]
239	62-53-3	CRC[1]	SDBS[2]
240	100-66-3	CRC[1]	SDBS[2]
241	71-43-2	CRC[1]	SDBS[2]
242	75-07-0	CRC[1]	SDBS[2]
243	100-52-7	CRC[1]	SDBS[2]
244	123-72-8	CRC[1]	SDBS[2]
245	1636-39-1	CRC[1]	SDBS[2]
246	107-88-0	CRC[1]	SDBS[2]
247	110-63-4	CRC[1]	Ref [4,20]
248	109-74-0	CRC[1]	SDBS[2]
249	107-92-6	CRC[1]	SDBS[2]
250	71-36-3	CRC[1]	SDBS[2]
251	78-92-2	CRC[1]	SDBS[2]
252	78-93-3	CRC[1]	SDBS[2]
253	109-75-1	CRC[1]	SDBS[2]
254	123-86-4	CRC[1]	SDBS[2]
255	109-73-9	CRC[1]	SDBS[2]
256	75-64-9	CRC[1]	SDBS[2]
257	104-51-8	CRC[1]	SDBS[2]
258	36383-15-0	CRC[1]	Ref [21]
259	98-06-6	CRC[1]	SDBS[2]
260	98-19-1	CRC[1]	SDBS[2]
261	1075-38-3	CRC[1]	SDBS[2]
262	98-51-1	CRC[1]	SDBS[2]
263	111-34-2	CRC[1]	SDBS[2]
264	108-39-4	CRC[1]	SDBS[2]
265	110-82-7	CRC[1]	SDBS[2]
266	287-92-3	CRC[1]	SDBS[2]
267	1975-78-6	CRC[1]	SDBS[2]
268	124-18-5	CRC[1]	SDBS[2]
269	142-96-1	CRC[1]	SDBS[2]
270	6863-58-7	CRC[1]	SDBS[2]
271	112-30-1	CRC[1]	SDBS[2]
272	872-05-9	CRC[1]	SDBS[2]
273	95-78-3	CRC[1]	SDBS[2]
274	75-83-2	CRC[1]	SDBS[2]
275	563-78-0	CRC[1]	SDBS[2]
276	1003-64-1	CRC[1]	Ref [19,22]
277	96-48-0	CRC[1]	SDBS[2]
278	110-83-8	CRC[1]	SDBS[2]
279	502-56-7	CRC[1]	SDBS[2]
280	591-78-6	CRC[1]	Ref [23,24]
281	108-94-1	CRC[1]	SDBS[2]

282	120-92-3	CRC[1]	SDBS[2]
283	506-30-9	CRC[1]	SDBS[2]
284	57-11-4	CRC[1]	SDBS[2]
285	506-12-7	CRC[1]	SDBS[2]
286	57-10-3	CRC[1]	Ref [25]
287	13269-52-8	CRC[1]	Ref [26]
288	766-05-2	CRC[1]	Ref [27,28]
289	5454-79-5	CRC[1]	Ref [29–31]
290	615-38-3	CRC[1]	Ref [29–31]
291	7688-21-3	CRC[1]	Ref [26,32,33]
292	6270-16-2	CRC[1]	Ref [19,34]
293	629-11-8	CRC[1]	SDBS[2]
294	124-13-0	Table S2	SDBS[2]
295	106-98-9	CRC[1]	Ref [14]
296	33966-50-6	CRC[1]	Ref [4,35]
297	591-68-4	CRC[1]	Ref [4,36]
298	143-07-7	CRC[1]	SDBS[2]<br>Ref [37]
299	111-14-8	CRC[1]	Ref [19,38]
300	102-82-9	CRC[1]	Ref [4,39]
301	7378-99-6	CRC[1]	Ref [19,40]
302	102-69-2	CRC[1]	Ref [19,41]
303	92-51-3	CRC[1]	SDBS[2]
304	1678-93-9	CRC[1]	SDBS[2]
305	590-66-9	CRC[1]	SDBS[2]
306	6876-23-9	CRC[1]	SDBS[2]
307	624-29-3	CRC[1]	SDBS[2]
308	2207-01-4	CRC[1]	SDBS[2]
309	104-72-3	CRC[1]	SDBS[2]
310	100-51-6	CRC[1]	SDBS[2]
311	563-79-1	CRC[1]	SDBS[2]
312	142-29-0	CRC[1]	SDBS[2]
313	108-91-8	CRC[1]	SDBS[2]
314	1003-03-8	CRC[1]	SDBS[2]
315	100-46-9	CRC[1]	SDBS[2]
316	66-25-1	Table S2	Ref [33,42]
317	334-48-5	CRC[1]	Ref [4,43,44]
318	1731-88-0	CRC[1]	Ref [45–47]
319	827-52-1	CRC[1]	SDBS[2]
320	142-77-8	CRC[1]	SDBS[2]
321	2040-96-2	CRC[1]	SDBS[2]
322	3524-75-2	CRC[1]	SDBS[2]
323	79-46-9	CRC[1]	Ref [33,48,49]
324	622-42-4	CRC[1]	Ref [50–52]
325	1638-26-2	CRC[1]	Ref [53]
326	123-75-1	CRC[1]	Ref [19,54]
327	4609-87-4	CRC[1]	Ref [55]
328	108-93-0	CRC[1]	SDBS[2]

329	96-41-3	CRC[1]	SDBS[2]
330	1002-84-2	CRC[1]	SDBS[2]<br>Ref [19,56]
331	121-44-8	CRC[1]	Figure S4
332	51-80-9	CRC[1]	Figure S3
333	121-69-7	CRC[1]	Figure S5
334	760-20-3	CRC[1]	Ref [4,57]
335	1731-84-6	CRC[1]	Ref [4,47]
336	111-82-0	CRC[1]	Ref [19,58]
337	593-45-3	Table S2	SDBS[2]
338	123-19-3	Table S2	SDBS[2]
339	95-48-7	Table S2	SDBS[2]
340	106-44-5	Table S2	SDBS[2]
341	108-95-2	Table S2	SDBS[2]
342	99-65-0	CRC[1]	SDBS[2]
343	100-47-0	CRC[1]	SDBS[2]
344	75-86-5	CRC[1]	SDBS[2]
345	98-95-3	CRC[1]	SDBS[2]
346	88-74-4	CRC[1]	SDBS[2]
347	88-72-2	CRC[1]	SDBS[2]
348	99-09-2	CRC[1]	SDBS[2]
349	100-01-6	CRC[1]	SDBS[2]
350	99-08-1	CRC[1]	SDBS[2]
351	107-11-9	CRC[1]	SDBS[2]
352	626-35-7	CRC[1]	SDBS[2]
353	75-85-4	CRC[1]	SDBS[2]
354	77-76-9	CRC[1]	SDBS[2]
355	126-84-1	CRC[1]	SDBS[2]
356	96-33-3	CRC[1]	SDBS[2]
357	140-88-5	CRC[1]	SDBS[2]
358	141-32-2	CRC[1]	SDBS[2]
359	79-10-7	CRC[1]	SDBS[2]
360	112-72-1	CRC[1]	SDBS[2]
361	612-17-9	CRC[1]	SDBS[2]
362	645-49-8	CRC[1]	SDBS[2]
363	1014-60-4	CRC[1]	SDBS[2]
364	447-53-0	CRC[1]	SDBS[2]

Table S1. CAS registry numbers of the compounds included in the dataset and the corresponding sources of thermodynamic and spectroscopic data.

H-NMR of 108-01-0 ~25mM in Chloroform-d 25°C, recorded at 400 MHz:

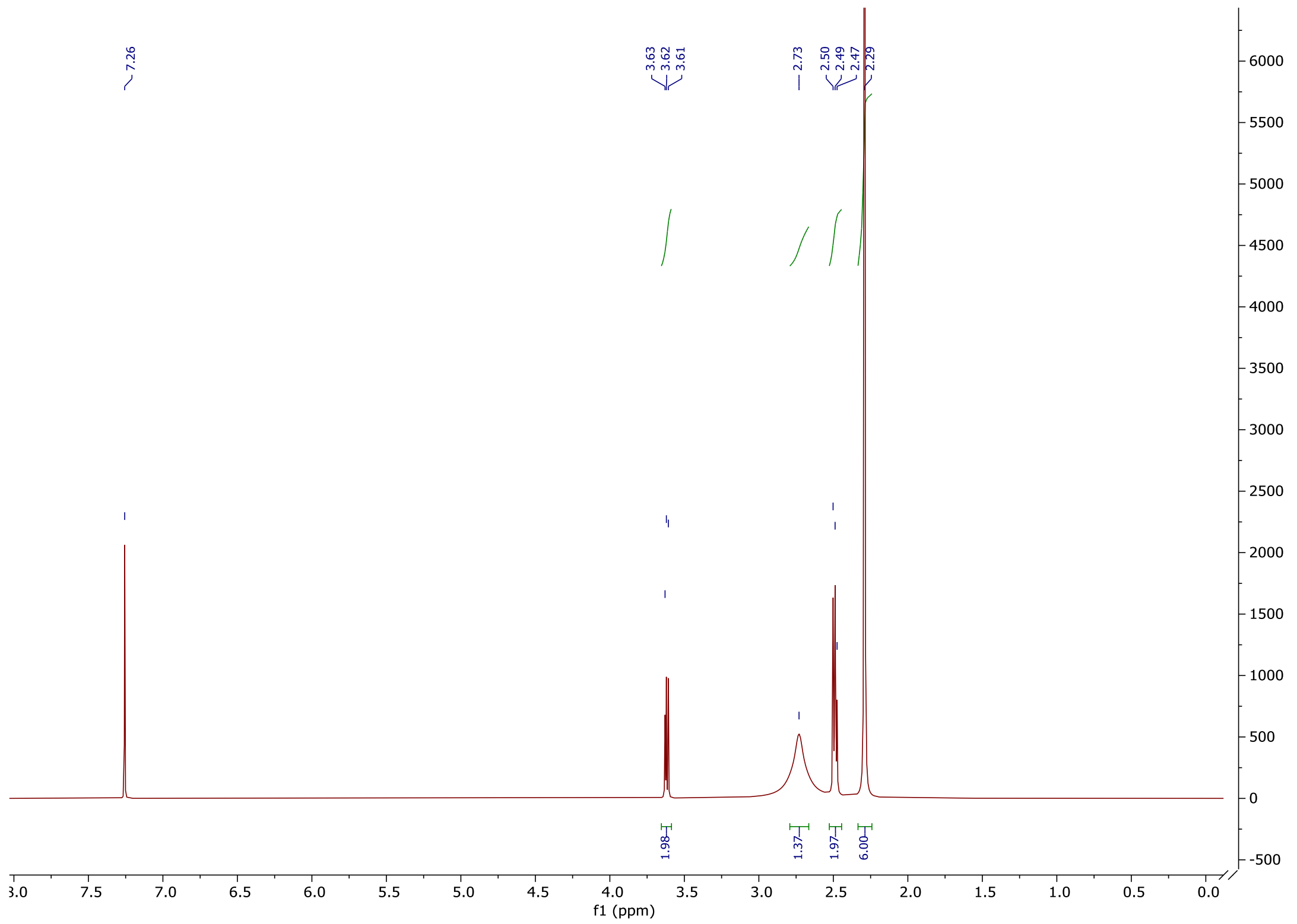

Figure S2: 108-01-0

H-NMR of 51-80-9 ~25mM in Chloroform-d 25°C, recorded at 400 MHz:

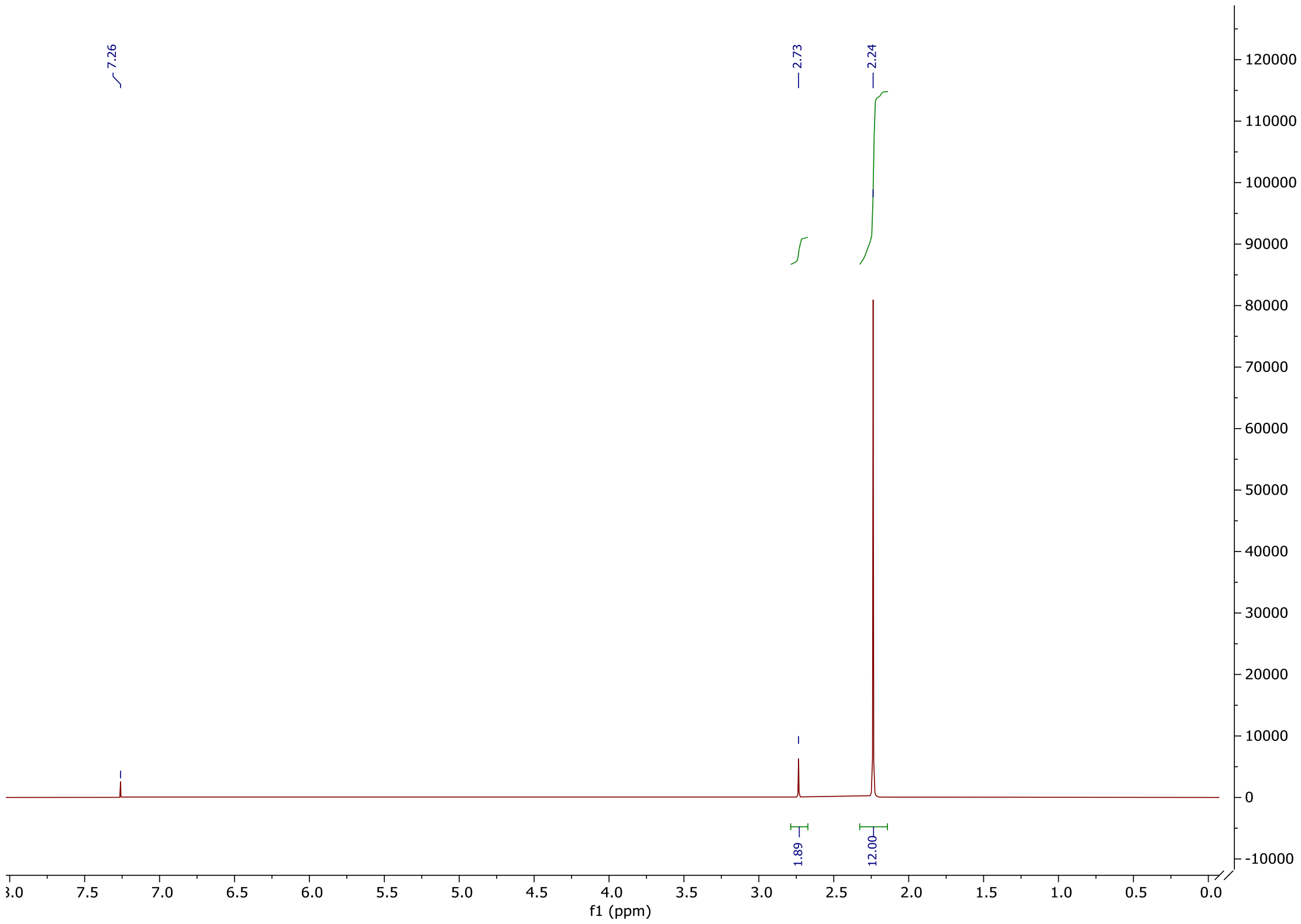

Figure S3: 51-80-9

H-NMR of 121-44-8 ~25mM in Chloroform-d 25°C, recorded at 400 MHz:

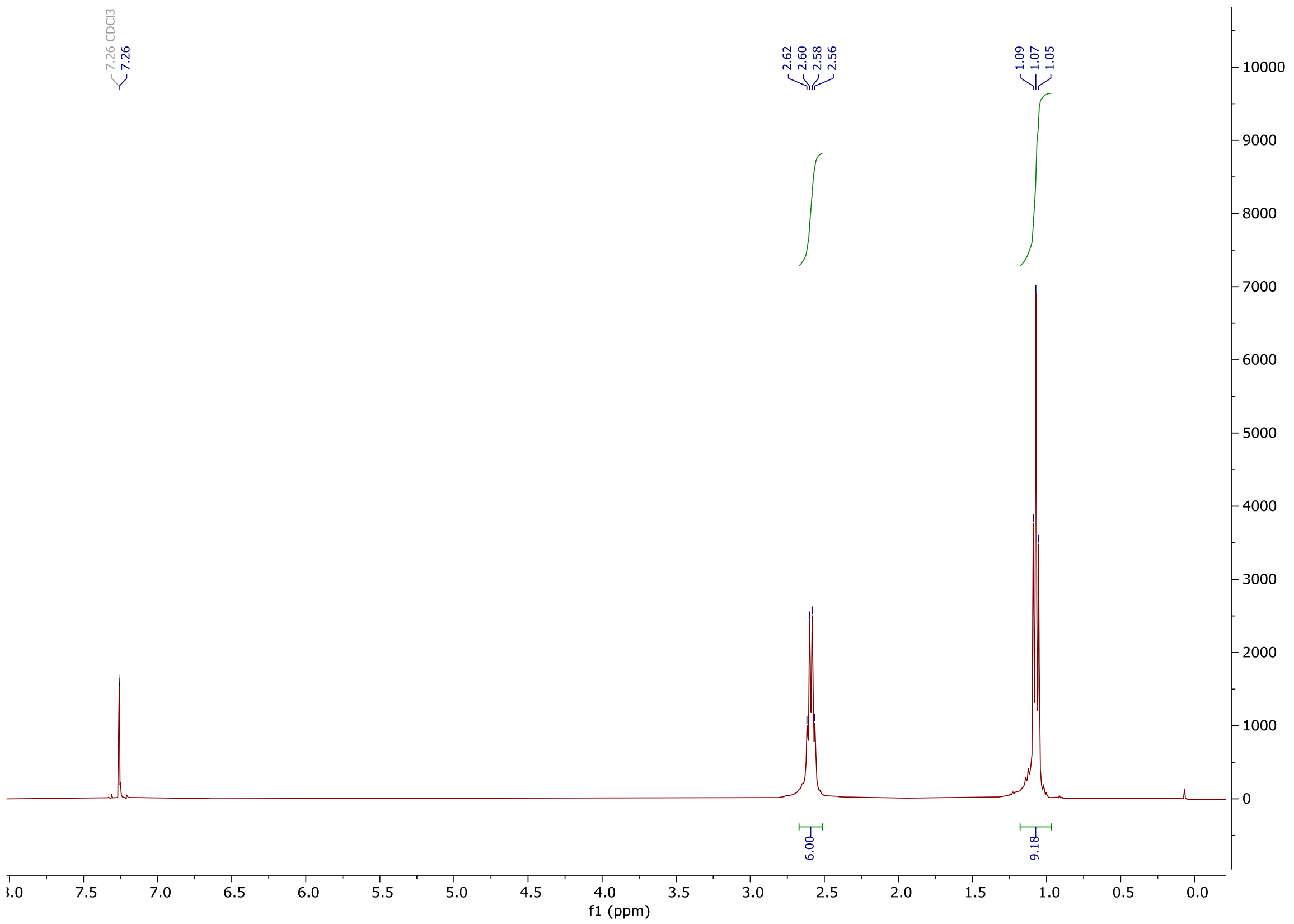

Figure S4: 121-44-8

H-NMR of 121-69-7 ~25mM in Chloroform-d 25°C, recorded at 400 MHz:

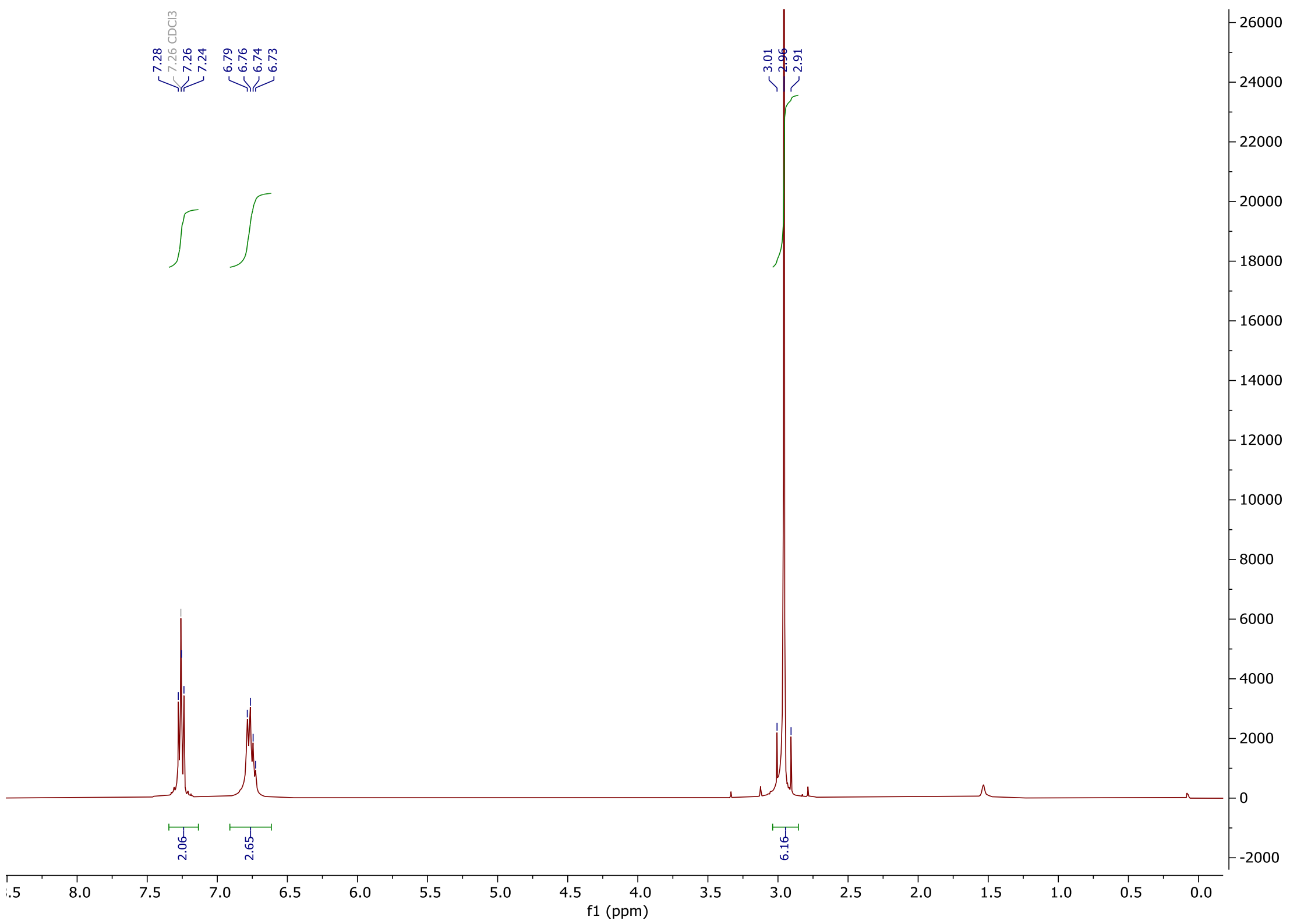

Figure S5: 121-69-7

References for enthalpies of formation not obtained from the CRC.

For the following molecules, the Standard formation enthalpy in liquid phase was calculated using the following equation:

$$\Delta H^{\circ}_{f,liquid} = \Delta H^{\circ}_{f,gas} - \Delta H^{\circ}_{vaporization}$$

The source of the values and the obtained standard liquid phase enthalpy of formation is presented in the following table (table S2).

CAS	Standard formation enthalpy in gas	Standard vaporization enthalpy	Standard formation enthalpy in liquid
66-25-1	-248.4 ± 1.6 $kjmol^{-1}$ (Ref [59])	42.5 ± 0.4 $kjmol^{-1}$ (Ref [60])	-291 $kjmol^{-1}$
124-13-0	-291.9 $kjmol^{-1}$ (CRC [1])	51.0 ± 0.3 $kjmol^{-1}$ (Ref [60])	-342.9 $kjmol^{-1}$
593-45-3	-414.6 $kjmol^{-1}$ (CRC [1])	90.6 ± 0.1 $kjmol^{-1}$ (Ref [61])	-505.2 $kjmol^{-1}$
123-19-3	-298.3 $kjmol^{-1}$ (CRC [1])	46.7 ± 0.4 $kjmol^{-1}$ (Ref [62])	-345 $kjmol^{-1}$
95-48-7	-128.6 $kjmol^{-1}$ (CRC [1])	44.9 ± 0.1 $kjmol^{-1}$ (Ref [63])	-173.5 $kjmol^{-1}$
106-44-5	-125.4 $kjmol^{-1}$ (CRC [1])	46.6 ± 0.1 $kjmol^{-1}$ (Ref [63])	-172 $kjmol^{-1}$
108-95-2	-96.4 $kjmol^{-1}$ (CRC [1])	58.8 ± 0.1 $kjmol^{-1}$ (Ref [64])	-155.2 $kjmol^{-1}$

Table S2: Gas-phase formation enthalpies and vaporization enthalpies used to derive liquid-phase standard enthalpies of formation.

S3 - Dataset used in this Study section:

CAS	Enthalpy	ShiftVecs	IntVecs	Exists
75-31-0	-112.3	3.102, 1.221, 1.071	1, 2, 6	0, 0, 0, 0, 0, 0, 1, 0, 0, 0, 0
95-53-4	-6.3	3.48, 7.01, 7, 6.676, 6.586, 2.09	2, 1, 1, 1, 1, 3	0, 0, 0, 0, 0, 0, 1, 0, 0, 0, 0
108-44-1	-8.1	3.46, 7.01, 6.56, 6.49, 6.47, 2.25	2, 1, 1, 1, 1, 3	0, 0, 0, 0, 0, 0, 1, 0, 0, 0, 0
811-93-8	-133.9	2.494, 1.38, 1.058	2, 4, 6	0, 0, 0, 0, 0, 0, 2, 0, 0, 0, 0
78-82-0	-13.8	1.331, 2.721	6, 1	0, 0, 0, 0, 0, 0, 0, 0, 0, 1, 0
124-12-9	-107.3	0.891, 1.34, 1.45, 1.652, 2.339	3, 6, 2, 2, 2	0, 0, 0, 0, 0, 0, 0, 0, 0, 1, 0
110-59-8	-33.1	0.959, 1.5, 1.63, 2.342	3, 2, 2, 2	0, 0, 0, 0, 0, 0, 0, 0, 0, 1, 0
107-10-8	-101.5	2.653, 1.24, 0.92, 1.45	2, 2, 3, 2	0, 0, 0, 0, 0, 0, 1, 0, 0, 0, 0
110-89-4	-86.4	2.79, 2.04, 1.58	4, 1, 6	0, 0, 0, 0, 0, 0, 0, 1, 0, 0, 0
107-12-0	15.5	1.3, 2.36	3, 2	0, 0, 0, 0, 0, 0, 0, 0, 0, 1, 0
629-63-0	-260.2	0.89, 1.26, 1.65, 2.32	3, 20, 2, 2	0, 0, 0, 0, 0, 0, 0, 0, 0, 1, 0
102-86-3	-433	2.403, 0.885, 1.42, 1.31, 1.28	6, 9, 6, 6, 12	0, 0, 0, 0, 0, 0, 0, 0, 1, 0, 0
1116-76-3	-585	2.37, 0.88, 1.27, 1.45	6, 9, 30, 6	0, 0, 0, 0, 0, 0, 0, 0, 1, 0, 0
75-52-5	-122.6	4.341	3	0, 0, 0, 0, 0, 0, 0, 0, 0, 0, 1
627-05-4	-192.5	1.065, 1.504, 2.065, 4.466	3, 2, 2, 2	0, 0, 0, 0, 0, 0, 0, 0, 0, 0, 1
79-24-3	-143.9	1.577, 4.428	3, 2	0, 0, 0, 0, 0, 0, 0, 0, 0, 0, 1
625-48-9	-350.7	4.545, 3.59, 4.11	2, 1, 2	1, 0, 0, 0, 0, 0, 0, 0, 0, 0, 1
628-05-7	-215.4	0.92, 1.5, 2.01, 4.379	3, 4, 2, 2	0, 0, 0, 0, 0, 0, 0, 0, 0, 0, 1
108-03-2	-167.2	1.029, 2.033, 4.361	3, 2, 2	0, 0, 0, 0, 0, 0, 0, 0, 0, 0, 1
108-01-0	-253.7	3.61, 2.265, 2.46, 3.67	2, 6, 2, 1	1, 0, 0, 0, 0, 0, 0, 0, 1, 0, 0
111-92-2	-206	2.594, 1.56, 0.916, 1.45	4, 1, 6, 8	0, 0, 0, 0, 0, 0, 0, 1, 0, 0, 0
100-37-8	-305.9	3.542, 2.59, 2.56, 3, 1.028	2, 2, 4, 1, 6	1, 0, 0, 0, 0, 0, 0, 0, 1, 0, 0
110-96-3	-218.5	2.392, 1.09, 0.905, 1.73	4, 1, 12, 2	0, 0, 0, 0, 0, 0, 0, 1, 0, 0, 0
108-18-9	-178.5	2.91, 1.042, 1	2, 1, 12	0, 0, 0, 0, 0, 0, 0, 1, 0, 0, 0
103-69-5	8.2	3.114, 3.42, 6.58, 6.68, 7.147, 1.216	2, 1, 2, 1, 2, 3	0, 0, 0, 0, 0, 0, 0, 1, 0, 0, 0
142-84-7	-156.1	2.564, 1.9, 0.914, 1.502	4, 1, 6, 4	0, 0, 0, 0, 0, 0, 0, 1, 0, 0, 0
629-08-3	-82.8	0.903, 1.32, 1.455, 1.651, 2.341	3, 4, 2, 2, 2	0, 0, 0, 0, 0, 0, 0, 0, 0, 1, 0
111-69-3	85.1	1.84, 2.43	4, 4	0, 0, 0, 0, 0, 0, 0, 0, 0, 2, 0
75-05-8	40.6	2	3	0, 0, 0, 0, 0, 0, 0, 0, 0, 1, 0
62-53-3	31.6	3.47, 6.66, 6.74, 7.135	2, 1, 2, 2	0, 0, 0, 0, 0, 0, 1, 0, 0, 0, 0
109-74-0	-5.8	1.081, 1.699, 2.339	3, 2, 2	0, 0, 0, 0, 0, 0, 0, 0, 0, 1, 0
109-73-9	-127.6	2.68, 1.77, 0.92, 1.33, 1.43	2, 2, 3, 2, 2	0, 0, 0, 0, 0, 0, 1, 0, 0, 0, 0
75-64-9	-150.6	1.41, 1.149	2, 9	0, 0, 0, 0, 0, 0, 1, 0, 0, 0, 0
1975-78-6	-158.4	0.883, 1.29, 1.442, 1.65, 2.334	3, 12, 2, 2, 2	0, 0, 0, 0, 0, 0, 0, 0, 0, 1, 0

95-78-3	-38.9	3.43, 6.47, 6.49, 6.9, 2.101, 2.225	2, 1, 1, 1, 3, 3	0, 0, 0, 0, 0, 0, 1, 0, 0, 0, 0
102-82-9	-281.6	2.43, 0.95, 1.335, 1.45	6, 9, 6, 6	0, 0, 0, 0, 0, 0, 0, 0, 1, 0, 0
7378-99-6	-232.8	2.215, 0.87, 1.28, 1.445	8, 3, 10, 2	0, 0, 0, 0, 0, 0, 0, 0, 1, 0, 0
102-69-2	-207.1	2.38, 0.85, 1.42	6, 9, 6	0, 0, 0, 0, 0, 0, 0, 0, 1, 0, 0
79-46-9	-180.3	1.56, 4.66	6, 1	0, 0, 0, 0, 0, 0, 0, 0, 0, 0, 1
622-42-4	-22.8	7.475, 5.44	5, 2	0, 0, 0, 0, 0, 0, 0, 0, 0, 0, 1
123-75-1	-41.1	2.835, 2.25, 1.655	4, 1, 4	0, 0, 0, 0, 0, 0, 0, 1, 0, 0, 0
4609-87-4	-351.5	0.85, 1.33, 4.175	3, 14, 2	0, 0, 0, 0, 0, 0, 0, 0, 0, 0, 1
766-05-2	-47.2	1.78, 1.65, 1.43, 2.75	2, 4, 4, 1	0, 0, 0, 0, 0, 0, 0, 0, 0, 1, 0
6270-16-2	-390	4.05, 2.87, 1.05, 1.35, 4.35	1, 1, 3, 3, 1	1, 0, 0, 0, 0, 0, 0, 0, 0, 0, 1
108-91-8	-147.6	2.62, 1.18, 1.97, 1.44	1, 2, 5, 5	0, 0, 0, 0, 0, 0, 1, 0, 0, 0, 0
1003-03-8	-95.1	3.314, 1.37, 1.82, 1.1715, 1.547, 1.277	1, 2, 2, 2, 2, 2	0, 0, 0, 0, 0, 0, 1, 0, 0, 0, 0
100-46-9	34.2	3.84, 1.52, 7.49	2, 2, 5	0, 0, 0, 0, 0, 0, 1, 0, 0, 0, 0
16529-66-1	80.9	5.829, 5.375, 1.729, 3.058	1, 1, 3, 2	0, 0, 0, 0, 0, 0, 0, 0, 0, 1, 0
109-75-1	117.8	5.722, 5.31, 5.46, 3.132	1, 1, 1, 2	0, 0, 0, 0, 0, 0, 0, 0, 0, 1, 0
121-44-8	-127.7	2.59, 1.07	6, 9	0, 0, 0, 0, 0, 0, 0, 0, 1, 0, 0
51-80-9	-51.1	2.24, 2.73	12, 2	0, 0, 0, 0, 0, 0, 0, 0, 2, 0, 0
121-69-7	46	3.06, 6.75, 7.26	6, 3, 2	0, 0, 0, 0, 0, 0, 0, 0, 1, 0, 0
33966-50-6	-137.5	2.8, 1.225, 1.075, 0.925, 1.35	1, 2, 3, 3, 2	0, 0, 0, 0, 0, 0, 1, 0, 0, 0, 0
594-70-7	-217.2	1.619	9	0, 0, 0, 0, 0, 0, 0, 0, 0, 0, 1
99-65-0	-36	9.083, 8.615, 7.871	1, 2, 1	0, 0, 0, 0, 0, 0, 0, 0, 0, 0, 2
100-47-0	163.2	7.33	5	0, 0, 0, 0, 0, 0, 0, 0, 0, 1, 0
75-86-5	-120.9	3.63, 1.627	1,6	1, 0, 0, 0, 0, 0, 0, 0, 0, 1, 0
98-95-3	12.5	8.245, 7.557, 7.705	2,2,1	0, 0, 0, 0, 0, 0, 0, 0, 0, 0, 1
88-74-4	-9.4	8.090, 7.336, 6.81, 6.68, 6.1	1, 1, 1, 1, 2	0, 0, 0, 0, 0, 0, 1, 0, 0, 0, 1
88-72-2	-9.7	7.94, 7.48, 7.41, 2.589	1,1,2,3	0, 0, 0, 0, 0, 0, 0, 0, 0, 0, 1
99-09-2	-14.4	7.54, 7.48, 7.26, 6.947, 4.0	1,1,1,1,2	0, 0, 0, 0, 0, 0, 1, 0, 0, 0, 1
100-01-6	-20.7	7.972, 6.71, 6.64	2, 2, 2	0, 0, 0, 0, 0, 0, 1, 0, 0, 0, 1
99-08-1	-31.5	8.09, 7.51, 7.39, 2.46	2, 1, 1, 3	0, 0, 0, 0, 0, 0, 0, 0, 0, 0, 1
107-11-9	-10	5.954, 5.159, 5.051, 3.311, 1.29	1,1,1,2,2	0, 0, 0, 0, 0, 0, 1, 0, 0, 0, 0
626-35-7	-487.1	5.204, 4.321, 1.328	2,2,3	0, 0, 0, 0, 1, 0, 0, 0, 0, 0, 1
75-85-4	-379.5	1.52, 1.49, 1.197, 0.924	1, 2, 6, 3	1, 0, 0, 0, 0, 0, 0, 0, 0, 0, 0
77-76-9	-459.4	3.193, 1.334	6,6	0, 0, 0, 0, 0, 2, 0, 0, 0, 0, 0
126-84-1	-538.9	3.471, 1.172, 1.352	4,6,6	0, 0, 0, 0, 0, 2, 0, 0, 0, 0, 0
96-33-3	-362.2	6.402, 6.128, 5.824, 3.759	1,1,1,3	0, 0, 0, 0, 1, 0, 0, 0, 0, 0, 0
140-88-5	-370.6	6.369, 6.130, 5.811, 4.214, 1.302	1,1,1,2,3	0, 0, 0, 0, 1, 0, 0, 0, 0, 0, 0

141-32-2	-422.6	0.96, 1.41, 1.65, 4.158, 5.795, 6.108, 6.364	3, 2, 2, 2, 1, 1, 1	0, 0, 0, 0, 1, 0, 0, 0, 0, 0, 0
79-10-7	-383.8	12.0, 6.52, 6.14, 5.96	1,1,1,1	0, 1, 0, 0, 0, 0, 0, 0, 0, 0, 0
112-72-1	-580.6	0.88, 1.26, 1.53, 1.56, 3.63	3, 22, 2, 1, 2	1, 0, 0, 0, 0, 0, 0, 0, 0, 0, 0
97-85-8	-587.4	2.525, 3.83, 0.91, 1.15, 1.9	1, 2, 6, 6, 1	0, 0, 0, 0, 1, 0, 0, 0, 0, 0, 0
10588-10-0	-620	2.313, 3.855, 0.932, 0.923, 1.356, 1.616, 1.924	2, 2, 6, 3, 2, 2, 1	0, 0, 0, 0, 1, 0, 0, 0, 0, 0, 0
78-84-2	-247.3	9.62, 2.405, 1.1	1, 1, 6	0, 0, 1, 0, 0, 0, 0, 0, 0, 0, 0
108-21-4	-518.9	2.017, 4.988, 1.233	3, 1, 6	0, 0, 0, 0, 1, 0, 0, 0, 0, 0, 0
88-69-7	-233.7	4.7, 6.71, 6.92, 7.04, 7.19, 3.2, 1.249	1, 1, 1, 1, 1, 1, 6	1, 0, 0, 0, 0, 0, 0, 0, 0, 0, 0
618-45-1	-252.5	4.76, 6.65, 6.71, 6.81, 7.16, 2.86, 1.23	1, 1, 1, 1, 1, 1, 6	1, 0, 0, 0, 0, 0, 0, 0, 0, 0, 0
67-56-1	-239.2	3.43, 3.66	3, 1	1, 0, 0, 0, 0, 0, 0, 0, 0, 0, 0
79-20-9	-445.9	2.05, 3.662	3, 3	0, 0, 0, 0, 1, 0, 0, 0, 0, 0, 0
93-58-3	-343.5	3.91, 7.425, 7.54, 8.035	3, 2, 1, 2	0, 0, 0, 0, 1, 0, 0, 0, 0, 0, 0
600-07-7	-554.5	2.42, 0.96, 1.17, 1.5, 1.695, 11.32	1, 3, 3, 1, 1, 1	0, 1, 0, 0, 0, 0, 0, 0, 0, 0, 0
503-74-2	-561.6	2.23, 0.99, 2.12, 11.92	1, 6, 1, 1	0, 1, 0, 0, 0, 0, 0, 0, 0, 0, 0
34713-94-5	-356.6	3.392, 3.482, 2.68, 0.9, 0.85, 1.15, 1.53	1, 1, 1, 3, 3, 2, 1	1, 0, 0, 0, 0, 0, 0, 0, 0, 0, 0
123-51-3	-356.4	3.673, 1.57, 0.922, 1.49, 1.66	2, 1, 6, 2, 1	1, 0, 0, 0, 0, 0, 0, 0, 0, 0, 0
70116-68-6	-366.6	3.55, 1.78, 0.9, 1.15, 1.6	1, 1, 6, 3, 1	1, 0, 0, 0, 0, 0, 0, 0, 0, 0, 0
563-80-4	-299.5	2.584, 2.141, 1.111	1, 3, 6	0, 0, 0, 1, 0, 0, 0, 0, 0, 0, 0
1634-04-4	-313.6	3.212, 1.189	3, 9	0, 0, 0, 0, 0, 1, 0, 0, 0, 0, 0
78-83-1	-334.7	3.391, 2.07, 0.92, 1.75	2, 1, 6, 1	1, 0, 0, 0, 0, 0, 0, 0, 0, 0, 0
75-65-0	-359.2	2.01, 1.262	1, 9	1, 0, 0, 0, 0, 0, 0, 0, 0, 0, 0
557-17-5	-266	3.336, 3.337, 0.93, 1.59	2, 3, 3, 2	0, 0, 0, 0, 0, 1, 0, 0, 0, 0, 0
124-10-7	-743.9	2.29, 3.661, 0.87, 1.26, 1.61	2, 3, 3, 20, 2	0, 0, 0, 0, 1, 0, 0, 0, 0, 0, 0
1731-86-8	-665.2	2.29, 3.66, 0.88, 1.26, 1.62	2, 3, 3, 14, 2	0, 0, 0, 0, 1, 0, 0, 0, 0, 0, 0
112-62-9	-734.5	2.287, 3.658, 5.6, 2.18, 0.89, 1.49, 1.62	2, 3, 2, 4, 3, 20, 2	0, 0, 0, 0, 1, 0, 0, 0, 0, 0, 0
1937-62-8	-737	2.29, 3.662, 5.63, 2.12, 0.88, 1.47, 1.64	2, 3, 2, 4, 3, 20, 2	0, 0, 0, 0, 1, 0, 0, 0, 0, 0, 0
111-11-5	-590.3	2.3, 3.67, 0.88, 1.28, 1.62	2, 3, 3, 8, 2	0, 0, 0, 0, 1, 0, 0, 0, 0, 0, 0
7132-64-1	-771	2.29, 3.66, 0.88, 1.26, 1.61	2, 3, 3, 22, 2	0, 0, 0, 0, 1, 0, 0, 0, 0, 0, 0
624-24-8	-514.2	2.311, 3.664, 0.921, 1.33, 1.61	2, 3, 3, 2, 2	0, 0, 0, 0, 1, 0, 0, 0, 0, 0, 0
565-67-3	-396.4	3.272, 1.51, 0.92, 0.93, 1.5, 1.88	1, 1, 6, 3, 2, 1	1, 0, 0, 0, 0, 0, 0, 0, 0, 0, 0
565-69-5	-325.9	2.625, 2.483, 1.096, 1.045	1, 2, 6, 3	0, 0, 0, 1, 0, 0, 0, 0, 0, 0, 0
106-73-0	-567.1	2.3, 3.662, 0.89, 1.14, 1.62	2, 3, 3, 6, 2	0, 0, 0, 0, 1, 0, 0, 0, 0, 0, 0
106-70-7	-540.2	2.29, 3.664, 0.9, 1.56, 1.62	2, 3, 3, 4, 2	0, 0, 0, 0, 1, 0, 0, 0, 0, 0, 0
1120-97-4	-416.1	5.027, 4.699, 4.078, 3.73, 3.7, 1.231, 1.736, 1.479	1, 1, 1, 1, 1, 3, 1, 1	0, 0, 0, 0, 0, 2, 0, 0, 0, 0, 0
497-26-7	-386.9	4.978, 3.966, 3.857, 1.371	1, 2, 2, 3	0, 0, 0, 0, 0, 2, 0, 0, 0, 0, 0
7731-28-4	-413.2	3.94, 1.52, 0.92, 1.86	1, 1, 3, 9	1, 0, 0, 0, 0, 0, 0, 0, 0, 0, 0

7731-29-5	-433.3	3.52, 2.02, 0.87, 2.12, 1.51	1, 1, 3, 4, 5	1, 0, 0, 0, 0, 0, 0, 0, 0, 0, 0
25144-05-2	-345.5	3.7, 2.79, 0.978, 1.94, 1.78, 1.7, 1.6, 1.53, 1.156	1, 1, 3, 2, 1, 1, 1, 1, 1	1, 0, 0, 0, 0, 0, 0, 0, 0, 0, 0
1120-72-5	-265.2	2.11, 2.29, 1.091, 2.007, 1.803, 1.495, 2.25	2, 1, 3, 1, 1, 1, 1	0, 0, 0, 1, 0, 0, 0, 0, 0, 0, 0
110-42-9	-640.5	2.29, 3.662, 0.88, 1.27, 1.61	2, 3, 3, 12, 2	0, 0, 0, 0, 1, 0, 0, 0, 0, 0, 0
598-98-1	-530	3.663, 1.2	3, 9	0, 0, 0, 0, 1, 0, 0, 0, 0, 0, 0
112-05-0	-659.7	2.34, 0.88, 1.27, 1.64, 10.9	2, 3, 10, 2, 1	0, 1, 0, 0, 0, 0, 0, 0, 0, 0, 0
143-08-8	-453.4	3.617, 1.73, 0.89, 1.28, 1.56	2, 1, 3, 12, 2	1, 0, 0, 0, 0, 0, 0, 0, 0, 0, 0
821-55-6	-397.2	2.4, 2.13, 0.88, 1.27, 1.57	2, 3, 3, 8, 2	0, 0, 0, 1, 0, 0, 0, 0, 0, 0, 0
124-07-2	-636	2.338, 0.885, 1.29, 1.63, 11.6	2, 3, 8, 2, 1	0, 1, 0, 0, 0, 0, 0, 0, 0, 0, 0
111-87-5	-426.5	3.604, 2.4, 0.884, 1.29, 1.55	2, 1, 3, 8, 4	1, 0, 0, 0, 0, 0, 0, 0, 0, 0, 0
110-62-3	-267.2	9.763, 2.419, 0.93, 1.35, 1.59	1, 2, 3, 2, 2	0, 0, 1, 0, 0, 0, 0, 0, 0, 0, 0
111-29-5	-528.8	3.645, 2.13, 1.57, 1.48	4, 2, 4, 2	2, 0, 0, 0, 0, 0, 0, 0, 0, 0, 0
109-52-4	-559.4	2.35, 0.93, 1.39, 1.62, 11.96	2, 3, 2, 2, 1	0, 1, 0, 0, 0, 0, 0, 0, 0, 0, 0
71-41-0	-351.6	3.63, 2.35, 0.91, 1.35, 1.57	2, 1, 3, 4, 2	1, 0, 0, 0, 0, 0, 0, 0, 0, 0, 0
6032-29-7	-365.2	3.79, 1.92, 0.928, 1.178, 1.52	1, 1, 3, 3, 4	1, 0, 0, 0, 0, 0, 0, 0, 0, 0, 0
107-87-9	-297.3	2.4, 2.13, 0.93, 1.6	2, 3, 3, 2	0, 0, 0, 1, 0, 0, 0, 0, 0, 0, 0
96-22-0	-296.5	2.439, 1.058	4, 6	0, 0, 0, 1, 0, 0, 0, 0, 0, 0, 0
591-80-0	-430.6	2.71, 2.71, 5.82, 5.07, 5.02, 11.36	2, 2, 1, 1, 1, 1	0, 1, 0, 0, 0, 0, 0, 0, 0, 0, 0
123-38-6	-215.6	9.793, 2.46, 1.113	1, 2, 3	0, 0, 1, 0, 0, 0, 0, 0, 0, 0, 0
504-63-2	-480.8	3.833, 2.75, 1.81	4, 2, 2	2, 0, 0, 0, 0, 0, 0, 0, 0, 0, 0
79-09-4	-510.7	2.38, 1.159, 11.73	2, 3, 1	0, 1, 0, 0, 0, 0, 0, 0, 0, 0, 0
71-23-8	-302.6	3.582, 2.26, 0.94, 1.57	2, 1, 3, 2	1, 0, 0, 0, 0, 0, 0, 0, 0, 0, 0
67-63-0	-318.1	4.008, 2.16, 1.2	1, 1, 6	1, 0, 0, 0, 0, 0, 0, 0, 0, 0, 0
141-06-0	-583	2.299, 4.027, 0.91, 0.94, 1.35, 1.61, 1.66	2, 2, 3, 3, 2, 2, 2	0, 0, 0, 0, 1, 0, 0, 0, 0, 0, 0
112-60-7	-981.7	3.707, 3.605, 3.673, 4.1	4, 4, 8, 2	2, 0, 0, 0, 0, 3, 0, 0, 0, 0, 0
109-99-9	-216.2	3.727, 1.841	4, 4	0, 0, 0, 0, 0, 1, 0, 0, 0, 0, 0
142-68-7	-258.3	3.65, 1.59	4, 6	0, 0, 0, 0, 0, 1, 0, 0, 0, 0, 0
542-28-9	-436.7	2.55, 4.34, 2.15	2, 2, 4	0, 0, 0, 0, 1, 0, 0, 0, 0, 0, 0
112-27-6	-804.3	3.72, 3.603, 3.672, 4.01	4, 4, 4, 2	2, 0, 0, 0, 0, 2, 0, 0, 0, 0, 0
84-74-2	-842.6	4.303, 7.7, 7.53, 0.97, 1.44, 1.7	4, 2, 2, 6, 4, 4	0, 0, 0, 0, 2, 0, 0, 0, 0, 0, 0
111-46-6	-628.5	3.718, 3.596, 4.47	4, 4, 2	2, 0, 0, 0, 0, 1, 0, 0, 0, 0, 0
60-29-7	-279.5	3.472, 1.206	4, 6	0, 0, 0, 0, 0, 1, 0, 0, 0, 0, 0
95-92-1	-805.5	4.359, 1.384	4, 6	0, 0, 0, 0, 2, 0, 0, 0, 0, 0, 0
84-66-2	-776.6	4.363, 7.71, 7.53, 1.366	4, 2, 2, 6	0, 0, 0, 0, 2, 0, 0, 0, 0, 0, 0
105-57-7	-491.4	4.686, 3.54, 1.214, 1.301	1, 4, 6, 3	0, 0, 0, 0, 0, 2, 0, 0, 0, 0, 0
629-14-1	-451.4	3.584, 3.54, 1.219	4, 4, 6	0, 0, 0, 0, 0, 2, 0, 0, 0, 0, 0

462-95-3	-450.5	4.678, 3.599, 1.225	2, 4, 6	0, 0, 0, 0, 0, 2, 0, 0, 0, 0, 0
565-80-0	-352.9	2.77, 1.081	2, 12	0, 0, 0, 1, 0, 0, 0, 0, 0, 0, 0
108-83-8	-408.5	2.255, 0.911, 2.139	4, 12, 2	0, 0, 0, 1, 0, 0, 0, 0, 0, 0, 0
108-20-3	-351.5	3.64, 1.13	2, 12	0, 0, 0, 0, 0, 1, 0, 0, 0, 0, 0
91-16-7	-290.3	3.858, 6.89	6, 4	0, 0, 0, 0, 0, 2, 0, 0, 0, 0, 0
110-71-4	-376.6	3.545, 3.394	4, 6	0, 0, 0, 0, 0, 2, 0, 0, 0, 0, 0
109-87-5	-377.8	4.572, 3.359	2, 6	0, 0, 0, 0, 0, 2, 0, 0, 0, 0, 0
4744_10_9	-443.6	4.29, 3.318, 0.912, 1.62	1, 6, 3, 2	0, 0, 0, 0, 0, 2, 0, 0, 0, 0, 0
534-15-6	-420.6	4.568, 3.309, 1.281	1, 6, 3	0, 0, 0, 0, 0, 2, 0, 0, 0, 0, 0
505-22-6	-379.7	4.853, 3.912, 1.78	2, 4, 2	0, 0, 0, 0, 0, 2, 0, 0, 0, 0, 0
123-91-1	-353.9	3.692	8	0, 0, 0, 0, 0, 2, 0, 0, 0, 0, 0
646-06-0	-333.5	4.902, 3.877	2, 4	0, 0, 0, 0, 0, 2, 0, 0, 0, 0, 0
75-84-3	-399.4	3.28, 2.05, 0.906	2, 1, 9	1, 0, 0, 0, 0, 0, 0, 0, 0, 0, 0
3938-95-2	-577.2	4.112, 1.195, 1.247	2, 9, 3	0, 0, 0, 0, 1, 0, 0, 0, 0, 0, 0
111-62-6	-775.8	2.283, 4.121, 5.344, 2.009, 0.881, 1.25, 1.27, 1.31, 1.616	2, 2, 2, 4, 3, 3, 8, 12, 2	0, 0, 0, 0, 1, 0, 0, 0, 0, 0, 0
539-82-2	-553	2.29, 4.122, 0.92, 1.255, 1.15	2, 2, 3, 3, 4	0, 0, 0, 0, 1, 0, 0, 0, 0, 0, 0
90-00-6	-208.8	4.8, 7.142, 7.081, 6.887, 6.757, 2.639, 1.237	1, 1, 1, 1, 1, 2, 3	1, 0, 0, 0, 0, 0, 0, 0, 0, 0, 0
620-17-7	-214.3	5.07, 7.146, 6.779, 6.683, 6.652, 2.595, 1.211	1, 1, 1, 1, 1, 2, 3	1, 0, 0, 0, 0, 0, 0, 0, 0, 0, 0
149-57-5	-635.1	2.285, 0.944, 0.897, 1.32, 1.5, 1.55, 1.64, 11.7	1, 3, 3, 4, 1, 1, 2, 1	0, 1, 0, 0, 0, 0, 0, 0, 0, 0, 0
104-76-7	-432.8	3.522, 3.496, 2.26, 0.89, 1.11, 1.15, 1.29, 1.34, 1.39, 1.479, 1.647	1, 1, 1, 6, 1, 1, 2, 1, 2, 1, 1	1, 0, 0, 0, 0, 0, 0, 0, 0, 0, 0
108-64-5	-571	2.2, 4.13, 0.958, 1.257, 2.1	2, 2, 6, 3, 1	0, 0, 0, 0, 1, 0, 0, 0, 0, 0, 0
107-21-1	-460	3.716, 3.17	4, 2	2, 0, 0, 0, 0, 0, 0, 0, 0, 0, 0
64-17-5	-277.6	3.687, 2.61, 1.226	2, 1, 3	1, 0, 0, 0, 0, 0, 0, 0, 0, 0, 0
103-73-1	-152.6	3.963, 7.22, 6.9, 6.8, 1.37	2, 2, 1, 2, 3	0, 0, 0, 0, 0, 1, 0, 0, 0, 0, 0
141-78-6	-479.3	2.038, 4.119, 1.26	3, 2, 3	0, 0, 0, 0, 1, 0, 0, 0, 0, 0, 0
111-43-3	-328.8	3.365, 0.93, 1.59	4, 6, 4	0, 0, 0, 0, 0, 1, 0, 0, 0, 0, 0
109-92-2	-167.4	3.74, 1.27, 6.46, 3.96, 4.17	2, 3, 1, 1, 1	0, 0, 0, 0, 0, 1, 0, 0, 0, 0, 0
105-37-3	-502.7	2.319, 4.132, 1.259, 1.14	2, 2, 3, 3	0, 0, 0, 0, 1, 0, 0, 0, 0, 0, 0
628-32-0	-303.6	3.465, 3.373, 1.203, 0.93, 1.6	2, 2, 3, 3, 2	0, 0, 0, 0, 0, 1, 0, 0, 0, 0, 0
111-71-7	-311.5	9.751, 2.4, 0.88, 1.3, 1.62	1, 2, 3, 4, 4	0, 0, 1, 0, 0, 0, 0, 0, 0, 0, 0
142-62-1	-583.8	2.348, 0.902, 1.33, 1.639, 11.2	2, 3, 4, 2, 1	0, 1, 0, 0, 0, 0, 0, 0, 0, 0, 0
111-27-3	-377.5	3.624, 1.79, 0.9, 1.32, 1.56	2, 1, 3, 4, 2	1, 0, 0, 0, 0, 0, 0, 0, 0, 0, 0
589-38-8	-320.2	2.41, 2.39, 1.06, 0.92, 1.6	2, 2, 3, 3, 2	0, 0, 0, 1, 0, 0, 0, 0, 0, 0, 0
107-18-6	-171.8	4.07, 3.11, 5.925, 5.21, 5.07	2, 1, 1, 1, 1	1, 0, 0, 0, 0, 0, 0, 0, 0, 0, 0
64-19-7	-484.3	2.098, 11.42	3, 1	0, 1, 0, 0, 0, 0, 0, 0, 0, 0, 0
67-64-1	-248.4	2.15, 2.15	3, 3	0, 0, 0, 1, 0, 0, 0, 0, 0, 0, 0

98-86-2	-142.5	2.55, 7.46, 7.52, 7.92	3, 2, 1, 2	0, 0, 0, 1, 0, 0, 0, 0, 0, 0, 0
100-66-3	-114.8	3.84, 6.94, 6.98, 7.33	3, 2, 1, 2	0, 0, 0, 0, 0, 1, 0, 0, 0, 0, 0
75-07-0	-192.2	2.206, 9.789	3, 1	0, 0, 1, 0, 0, 0, 0, 0, 0, 0, 0
100-52-7	-87	10.02, 7.56, 7.64, 7.87	1, 2, 1, 2	0, 0, 1, 0, 0, 0, 0, 0, 0, 0, 0
123-72-8	-239.2	9.764, 2.37, 0.97, 1.64	1, 2, 3, 2	0, 0, 1, 0, 0, 0, 0, 0, 0, 0, 0
107-88-0	-501	3.79, 4.02, 3.89, 1.212, 1.668	2, 1, 2, 3, 2	2, 0, 0, 0, 0, 0, 0, 0, 0, 0, 0
110-63-4	-505.3	3.63, 3.08, 1.64	4, 2, 4	2, 0, 0, 0, 0, 0, 0, 0, 0, 0, 0
107-92-6	-533.8	2.334, 0.983, 1.675, 11.51	2, 3, 2, 1	0, 1, 0, 0, 0, 0, 0, 0, 0, 0, 0
71-36-3	-327.3	3.626, 2.24, 0.94, 1.39, 1.53	2, 1, 3, 2, 2	1, 0, 0, 0, 0, 0, 0, 0, 0, 0, 0
78-92-2	-342.6	3.709, 2.37, 0.93, 1.173, 1.46	1, 1, 3, 3, 2	1, 0, 0, 0, 0, 0, 0, 0, 0, 0, 0
78-93-3	-273.3	2.449, 2.139, 1.058	2, 3, 3	0, 0, 0, 1, 0, 0, 0, 0, 0, 0, 0
123-86-4	-529.2	2.038, 4.062, 0.943, 1.39, 1.6	3, 2, 3, 2, 2	0, 0, 0, 0, 1, 0, 0, 0, 0, 0, 0
111-34-2	-218.8	3.675, 0.94, 1.39, 1.61, 6.459, 3.964	2, 3, 2, 2, 1, 2	0, 0, 0, 0, 0, 1, 0, 0, 0, 0, 0
108-39-4	-194	5.06, 6.64, 6.65, 6.75, 7.117, 2.297	1, 1, 1, 1, 1, 3	1, 0, 0, 0, 0, 0, 0, 0, 0, 0, 0
142-96-1	-377.9	3.4, 0.92, 1.38, 1.54	4, 6, 4, 4	0, 0, 0, 0, 0, 1, 0, 0, 0, 0, 0
6863-58-7	-401.5	3.35, 0.91, 1.1, 1.48	2, 6, 6, 4	0, 0, 0, 0, 0, 1, 0, 0, 0, 0, 0
112-30-1	-478.1	3.613, 1.84, 0.89, 1.27, 1.56	2, 1, 3, 14, 2	1, 0, 0, 0, 0, 0, 0, 0, 0, 0, 0
96-48-0	-420.9	2.483, 4.35, 2.28	2, 2, 2	0, 0, 0, 0, 1, 0, 0, 0, 0, 0, 0
502-56-7	-398.2	2.382, 0.91, 1.3, 1.54	4, 6, 4, 4	0, 0, 0, 1, 0, 0, 0, 0, 0, 0, 0
591-78-6	-322	2.43, 2.14, 0.9, 1.32, 1.56	2, 3, 3, 2, 2	0, 0, 0, 1, 0, 0, 0, 0, 0, 0, 0
108-94-1	-271.2	2.35, 2.07	4, 6	0, 0, 0, 1, 0, 0, 0, 0, 0, 0, 0
120-92-3	-235.9	2.33, 2.33	4, 4	0, 0, 0, 1, 0, 0, 0, 0, 0, 0, 0
506-30-9	-940	2.34, 0.88, 1.25, 1.64, 11	2, 3, 32, 2, 1	0, 1, 0, 0, 0, 0, 0, 0, 0, 0, 0
57-11-4	-884.7	2.35, 0.88, 1.26, 1.64, 11.2	2, 3, 28, 2, 1	0, 1, 0, 0, 0, 0, 0, 0, 0, 0, 0
506-12-7	-865.6	2.34, 0.89, 1.26, 1.63, 11.3	2, 3, 26, 2, 1	0, 1, 0, 0, 0, 0, 0, 0, 0, 0, 0
57-10-3	-838.1	2.35, 0.9, 1.27, 1.63, 11.8	2, 3, 24, 2, 1	0, 1, 0, 0, 0, 0, 0, 0, 0, 0, 0
544-63-8	-788.8	2.34, 0.88, 1.295, 1.63, 11.7	2, 3, 20, 2, 1	0, 1, 0, 0, 0, 0, 0, 0, 0, 0, 0
143-07-7	-737.9	2.33, 0.89, 1.3, 1.65, 11.5	2, 3, 16, 2, 1	0, 1, 0, 0, 0, 0, 0, 0, 0, 0, 0
111-14-8	-610.2	2.35, 0.89, 1.315, 1.63, 11.17	2, 3, 6, 2, 1	0, 1, 0, 0, 0, 0, 0, 0, 0, 0, 0
142-77-8	-816.9	2.27, 4.061, 5.34, 2.01, 0.88, 0.94, 1.43, 1.5, 1.61	2, 2, 2, 4, 3, 3, 22, 2, 2	0, 0, 0, 0, 1, 0, 0, 0, 0, 0, 0
5454-79-5	-416.1	3.54, 2, 0.88, 1.255, 1.625, 1.915	1, 1, 3, 4, 3, 2	1, 0, 0, 0, 0, 0, 0, 0, 0, 0, 0
615-38-3	-390.2	3.78, 1.93, 0.98, 1.3, 1.64	1, 1, 3, 4, 5	1, 0, 0, 0, 0, 0, 0, 0, 0, 0, 0
629-11-8	-548.6	3.62, 2.42, 1.44, 1.57	4, 2, 4, 4	2, 0, 0, 0, 0, 0, 0, 0, 0, 0, 0
100-51-6	-160.7	4.595, 2.3, 7.4	2, 1, 5	1, 0, 0, 0, 0, 0, 0, 0, 0, 0, 0
1002-84-2	-811.7	2.34, 0.89, 1.26, 1.63, 10.6	2, 3, 22, 2, 1	0, 1, 0, 0, 0, 0, 0, 0, 0, 0, 0
108-93-0	-348.2	3.58, 2.63, 1.52	1, 1, 10	1, 0, 0, 0, 0, 0, 0, 0, 0, 0, 0

96-41-3	-300.1	4.31, 2.03, 1.655	1, 1, 8	1, 0, 0, 0, 0, 0, 0, 0, 0, 0, 0
66-25-1	-342.9	9.758, 2.41, 0.88, 1.3, 1.638	1, 2, 3, 8, 2	0, 0, 1, 0, 0, 0, 0, 0, 0, 0, 0
334-48-5	-684.3	2.35, 0.88, 1.295, 1.62, 11.9	2, 3, 12, 2, 1	0, 1, 0, 0, 0, 0, 0, 0, 0, 0, 0
1731-88-0	-717.9	2.3, 3.67, 0.87, 1.585, 1.25	2, 3, 3, 2, 18	0, 0, 0, 0, 1, 0, 0, 0, 0, 0, 0
1731-84-6	-616.2	2.26, 3.62, 0.86, 1.52, 1.24	2, 3, 3, 2, 10	0, 0, 0, 0, 1, 0, 0, 0, 0, 0, 0
111-82-0	-693	2.28, 3.64, 0.86, 1.265, 1.6	2, 3, 3, 16, 2	0, 0, 0, 0, 1, 0, 0, 0, 0, 0, 0
591-68-4	-613.3	2.26, 4.04, 0.87, 1.58, 1.305	2, 2, 6, 4, 4	0, 0, 0, 0, 1, 0, 0, 0, 0, 0, 0
124-13-0	-290.9	9.79, 2.37, 0.905, 1.35, 1.65	1, 2, 3, 4, 2	0, 0, 1, 0, 0, 0, 0, 0, 0, 0, 0
25144-05-2	-345	2.36, 0.916, 1.59	4, 6, 4	0, 0, 0, 1, 0, 0, 0, 0, 0, 0, 0
95-48-7	-173.5	4.79, 7.11, 7.07, 6.82, 6.74, 2.236	1, 1, 1, 1, 1, 3	1, 0, 0, 0, 0, 0, 0, 0, 0, 0, 0
106-44-5	-172	5.2, 7.002, 6.721, 2.254	1, 2, 2, 3	1, 0, 0, 0, 0, 0, 0, 0, 0, 0, 0
108-95-2	-155.2	5.35, 7.24, 6.931, 6.838	1, 2, 2, 1	1, 0, 0, 0, 0, 0, 0, 0, 0, 0, 0
612-17-9	84.2	7.09, 5.883, 3.349	4,2,4	0, 0, 0, 0, 0, 0, 0, 0, 0, 0, 0
645-49-8	183.3	7.38, 6.572	10, 2	0, 0, 0, 0, 0, 0, 0, 0, 0, 0, 0
1014-60-4	-188.8	7.433, 7.21, 1.323	1,3,18	0, 0, 0, 0, 0, 0, 0, 0, 0, 0, 0
447-53-0	71.6	7.29, 6.421, 5.988, 2.75, 2.28	4, 1, 1, 2, 2	0, 0, 0, 0, 0, 0, 0, 0, 0, 0, 0
496-11-7	11.5	7.265, 7.165, 2.95, 2.1	2, 2, 4, 2	0, 0, 0, 0, 0, 0, 0, 0, 0, 0, 0
538-93-2	-69.8	7.27, 7.14, 7.18, 2.47, 0.9, 1.87	2, 2, 1, 2, 6, 1	0, 0, 0, 0, 0, 0, 0, 0, 0, 0, 0
98-82-8	-41.1	7.2, 7.255, 7.32, 2.94, 1.29	1, 2, 2, 1, 6	0, 0, 0, 0, 0, 0, 0, 0, 0, 0, 0
527-84-4	-73.3	7.17, 3.15, 2.35, 1.24	4, 1, 3, 6	0, 0, 0, 0, 0, 0, 0, 0, 0, 0, 0
535-77-3	-78.6	7.05, 7.17, 2.87, 2.3, 1.25	2, 2, 1, 3, 6	0, 0, 0, 0, 0, 0, 0, 0, 0, 0, 0
99-87-6	-78	7.135, 2.89, 2.34, 1.26	4, 1, 3, 6	0, 0, 0, 0, 0, 0, 0, 0, 0, 0, 0
78-78-4	-178.4	0.865, 0.869, 1.2, 1.447	3, 6, 2, 1	0, 0, 0, 0, 0, 0, 0, 0, 0, 0, 0
78-79-5	48.2	1.85, 6.44, 4.99, 5.06, 5.19	3, 1, 2, 1, 1	0, 0, 0, 0, 0, 0, 0, 0, 0, 0, 0
563-46-2	-61.1	2.015, 1.728, 1.031, 4.67	2, 3, 3, 2	0, 0, 0, 0, 0, 0, 0, 0, 0, 0, 0
563-45-1	-51.5	2.27, 0.95, 5.8, 4.9	1, 6, 1, 2	0, 0, 0, 0, 0, 0, 0, 0, 0, 0, 0
513-35-9	-68.6	1.56, 1.6, 1.68, 5.19	3, 3, 3, 1	0, 0, 0, 0, 0, 0, 0, 0, 0, 0, 0
871-83-0	-309.8	0.87, 0.889, 1.16, 1.27, 1.31, 1.522	6, 3, 2, 8, 2, 1	0, 0, 0, 0, 0, 0, 0, 0, 0, 0, 0
107-83-5	-204.6	0.864, 0.88, 1.29, 1.15, 1.541	6, 3, 2, 2, 1	0, 0, 0, 0, 0, 0, 0, 0, 0, 0, 0
763-29-1	-90	1.703, 1.98, 0.9, 1.47, 4.68	3, 2, 3, 2, 2	0, 0, 0, 0, 0, 0, 0, 0, 0, 0, 0
691-37-2	-80	1.94, 0.9, 1.63, 5.77, 4.97, 4.96	2, 6, 1, 1, 1, 1	0, 0, 0, 0, 0, 0, 0, 0, 0, 0, 0
625-27-4	-98.5	1.68, 1.6, 1.972, 0.932, 5.113	3, 3, 2, 3, 1	0, 0, 0, 0, 0, 0, 0, 0, 0, 0, 0
691-38-3	-87	5.306, 5.209, 1.608, 2.618, 0.944	1, 1, 3, 1, 6	0, 0, 0, 0, 0, 0, 0, 0, 0, 0, 0
592-27-8	-255	0.87, 0.889, 1.29, 1.26, 1.16, 1.524	6, 3, 2, 4, 2, 1	0, 0, 0, 0, 0, 0, 0, 0, 0, 0, 0
589-53-7	-251.6	0.839, 0.877, 1.26, 1.08, 1.34, 1.41	3, 6, 4, 2, 2, 1	0, 0, 0, 0, 0, 0, 0, 0, 0, 0, 0
591-76-4	-229.5	0.867, 0.889, 1.17, 1.27, 1.518	6, 3, 2, 4, 1	0, 0, 0, 0, 0, 0, 0, 0, 0, 0, 0

3524-73-0	-100	2.05, 0.889, 1.26, 1.55, 5.795, 4.98, 4.91	2, 6, 2, 1, 1, 1, 1	0, 0, 0, 0, 0, 0, 0, 0, 0, 0, 0
90-12-0	56.3	7.958, 7.808, 7.672, 7.48, 7.45, 7.336, 7.279, 2.654	1, 1, 1, 1, 1, 1, 1, 3	0, 0, 0, 0, 0, 0, 0, 0, 0, 0, 0
1192-37-6	-61.3	2.11, 1.78, 4.574	4, 6, 2	0, 0, 0, 0, 0, 0, 0, 0, 0, 0, 0
108-87-2	-190.1	0.87, 1.84, 1.49	3, 5, 6	0, 0, 0, 0, 0, 0, 0, 0, 0, 0, 0
96-37-7	-137.9	0.97, 1.8, 1.75, 1.18	3, 1, 6, 2	0, 0, 0, 0, 0, 0, 0, 0, 0, 0, 0
693-89-0	-36.4	2.36, 1.72, 1.9, 5.3	4, 3, 2, 1	0, 0, 0, 0, 0, 0, 0, 0, 0, 0, 0
111-84-2	-274.7	0.88, 1.29, 1.27	6, 4, 10	0, 0, 0, 0, 0, 0, 0, 0, 0, 0, 0
111-65-9	-250.1	0.88, 1.26	6, 12	0, 0, 0, 0, 0, 0, 0, 0, 0, 0, 0
111-66-0	-124.5	2.02, 0.87, 1.53, 5.77, 4.97, 4.91	2, 3, 8, 1, 1, 1	0, 0, 0, 0, 0, 0, 0, 0, 0, 0, 0
7642_04_8	-135.7	5.66, 1.598, 2.03, 0.9, 1.52	2, 3, 2, 3, 6	0, 0, 0, 0, 0, 0, 0, 0, 0, 0, 0
13389-42-9	-135.7	5.47, 1.633, 1.95, 0.883, 1.52	2, 3, 2, 3, 6	0, 0, 0, 0, 0, 0, 0, 0, 0, 0, 0
109-67-1	-46.9	2.02, 0.91, 1.43, 5.81, 4.97, 4.93	2, 3, 2, 1, 1, 1	0, 0, 0, 0, 0, 0, 0, 0, 0, 0, 0
627-20-3	-53.7	5.64, 2.04, 1.6, 0.96	2, 2, 3, 3	0, 0, 0, 0, 0, 0, 0, 0, 0, 0, 0
646-04-8	-58.2	5.47, 5.4, 1.97, 1.637, 0.96	1, 1, 2, 3, 3	0, 0, 0, 0, 0, 0, 0, 0, 0, 0, 0
109-66-0	-173.5	0.884, 1.3, 1.26	6, 4, 2	0, 0, 0, 0, 0, 0, 0, 0, 0, 0, 0
1678-92-8	-237.4	0.87, 1.53, 1.84, 1.53	3, 4, 5, 6	0, 0, 0, 0, 0, 0, 0, 0, 0, 0, 0
1070-87-7	-280	0.977, 1.259	18, 2	0, 0, 0, 0, 0, 0, 0, 0, 0, 0, 0
16747-38-9	-277.9	0.806, 0.681, 1.66	12, 6, 2	0, 0, 0, 0, 0, 0, 0, 0, 0, 0, 0
119-64-2	-29.2	7.05, 2.75, 1.79	4, 4, 4	0, 0, 0, 0, 0, 0, 0, 0, 0, 0, 0
526-73-8	-58.5	7, 6.95, 2.267, 2.157	1, 2, 6, 3	0, 0, 0, 0, 0, 0, 0, 0, 0, 0, 0
108-67-8	-63.4	6.78, 2.26	3, 9	0, 0, 0, 0, 0, 0, 0, 0, 0, 0, 0
464-06-2	-236.5	0.834, 0.83, 1.378	9, 6, 1	0, 0, 0, 0, 0, 0, 0, 0, 0, 0, 0
594-56-9	-177.7	1.751, 1.06, 4.722, 4.656	3, 9, 1, 1	0, 0, 0, 0, 0, 0, 0, 0, 0, 0, 0
108-88-3	12.4	7.26, 7.16, 2.36	2, 3, 3	0, 0, 0, 0, 0, 0, 0, 0, 0, 0, 0
3522-94-9	-293.3	0.862, 0.88, 1.14, 1.44	9, 6, 4, 1	0, 0, 0, 0, 0, 0, 0, 0, 0, 0, 0
540-84-1	-259.2	0.891, 0.907, 1.122, 1.659	9, 6, 2, 1	0, 0, 0, 0, 0, 0, 0, 0, 0, 0, 0
107-39-1	-145.9	1.94, 1.78, 0.926, 4.83, 4.63	2, 3, 9, 1, 1	0, 0, 0, 0, 0, 0, 0, 0, 0, 0, 0
107-40-4	-142.4	1.713, 1.664, 1.087, 5.164	3, 3, 9, 1	0, 0, 0, 0, 0, 0, 0, 0, 0, 0, 0
95-47-6	-24.4	7.07, 2.22	4, 6	0, 0, 0, 0, 0, 0, 0, 0, 0, 0, 0
108-38-3	-25.4	7.109, 6.96, 6.94, 2.278	1, 1, 2, 6	0, 0, 0, 0, 0, 0, 0, 0, 0, 0, 0
106-42-3	-24.4	7.046, 2.296	4, 6	0, 0, 0, 0, 0, 0, 0, 0, 0, 0, 0
1120-21-4	-327.2	0.89, 1.26	6, 18	0, 0, 0, 0, 0, 0, 0, 0, 0, 0, 0
638-04-0	-222.9	0.865, 1.8, 1.4, 1.09	6, 4, 4, 2	0, 0, 0, 0, 0, 0, 0, 0, 0, 0, 0
2207_03_6	-215.7	0.895, 1.77, 1.51, 1.46, 1.273, 1.12	6, 2, 2, 2, 2, 2	0, 0, 0, 0, 0, 0, 0, 0, 0, 0, 0
2207_04_7	-222.4	0.861, 1.65, 1.25, 0.92	6, 4, 4, 2	0, 0, 0, 0, 0, 0, 0, 0, 0, 0, 0
135-01-3	-68.5	7.14, 2.64, 1.217	4, 4, 6	0, 0, 0, 0, 0, 0, 0, 0, 0, 0, 0

141-93-5	-73.5	7.19, 7.03, 7, 2.617, 1.232	1, 1, 2, 4, 6	0, 0, 0, 0, 0, 0, 0, 0, 0, 0, 0
105-05-5	-72.8	7.11, 2.605, 1.22	4, 4, 6	0, 0, 0, 0, 0, 0, 0, 0, 0, 0, 0
590-35-2	-238.3	0.864, 0.88, 1.18	9, 3, 4	0, 0, 0, 0, 0, 0, 0, 0, 0, 0, 0
565-59-3	-233.1	0.788, 0.796, 0.856, 0.863, 1.1, 1.36, 1.17, 1.56	3, 3, 3, 3, 1, 1, 1, 1	0, 0, 0, 0, 0, 0, 0, 0, 0, 0, 0
108-08-7	-234.6	0.853, 1.028, 1.619	12, 2, 2	0, 0, 0, 0, 0, 0, 0, 0, 0, 0, 0
562-49-2	-234.2	0.792, 0.797, 1.203	6, 6, 4	0, 0, 0, 0, 0, 0, 0, 0, 0, 0, 0
2213-32-3	-117	1.683, 1.882, 0.87, 1.759, 4.719, 4.641	2, 3, 6, 1, 1, 1	0, 0, 0, 0, 0, 0, 0, 0, 0, 0, 0
762-62-9	-110.6	1.94, 0.888, 5.81, 5.02, 5.03	2, 9, 1, 1, 1	0, 0, 0, 0, 0, 0, 0, 0, 0, 0, 0
625-65-0	-123.1	1.657, 1.606, 2.47, 0.92, 4.935	3, 3, 1, 6, 1	0, 0, 0, 0, 0, 0, 0, 0, 0, 0, 0
590-73-8	-261.9	0.865, 0.9, 1.28, 1.23, 1.17	9, 3, 2, 2, 2	0, 0, 0, 0, 0, 0, 0, 0, 0, 0, 0
584-94-1	-252.6	0.853, 0.883, 0.781, 0.798, 1.28, 1.06, 1.22, 1.35, 1.54	3, 3, 3, 3, 2, 1, 1, 1, 1	0, 0, 0, 0, 0, 0, 0, 0, 0, 0, 0
589-43-5	-257	0.858, 0.866, 0.84, 0.827, 1.11, 1.11, 0.97, 1.31, 1.63, 1.38	3, 3, 3, 3, 1, 1, 1, 1, 1, 1	0, 0, 0, 0, 0, 0, 0, 0, 0, 0, 0
592-13-2	-260.4	0.873, 1.17, 1.14, 1.48	12, 2, 2, 2	0, 0, 0, 0, 0, 0, 0, 0, 0, 0, 0
100-41-4	-12.3	7.26, 2.63, 1.22	5, 2, 3	0, 0, 0, 0, 0, 0, 0, 0, 0, 0, 0
760-21-4	-87.1	2.033, 1.036, 4.692	4, 6, 2	0, 0, 0, 0, 0, 0, 0, 0, 0, 0, 0
1678-91-7	-212.1	0.86, 1.19, 1.74, 1.21, 1.13, 0.85, 0.86	3, 2, 5, 2, 1, 2, 1	0, 0, 0, 0, 0, 0, 0, 0, 0, 0, 0
1640-89-7	-163.4	0.9, 1.31, 1.85, 1.08	3, 2, 7, 2	0, 0, 0, 0, 0, 0, 0, 0, 0, 0, 0
2146-38-5	-53.3	2.24, 1.86, 1.041, 2.07, 5.31	4, 2, 3, 2, 1	0, 0, 0, 0, 0, 0, 0, 0, 0, 0, 0
874-41-9	-84.1	7.02, 6.96, 6.95, 2.573, 2.27, 2.26, 1.182	1, 1, 1, 2, 3, 3, 3	0, 0, 0, 0, 0, 0, 0, 0, 0, 0, 0
1758-88-9	-84.8	6.97, 2.57, 2.28, 2.245, 1.188	3, 2, 3, 3, 3	0, 0, 0, 0, 0, 0, 0, 0, 0, 0, 0
16747-32-3	-272.7	0.854, 0.92, 1.503, 1.077, 0.733	6, 9, 2, 2, 1	0, 0, 0, 0, 0, 0, 0, 0, 0, 0, 0
1068-87-7	-269.7	0.845, 0.876, 0.904, 1.25, 1.74, 0.785	6, 6, 3, 2, 2, 1	0, 0, 0, 0, 0, 0, 0, 0, 0, 0, 0
609-26-7	-249.6	0.828, 0.856, 1.21, 1.31, 1.69, 0.91	6, 6, 2, 2, 1, 1	0, 0, 0, 0, 0, 0, 0, 0, 0, 0, 0
1067-08-9	-252.8	0.757, 0.742, 1.193	9, 3, 6	0, 0, 0, 0, 0, 0, 0, 0, 0, 0, 0
617-78-7	-224.9	0.843, 1.28, 1.1	9, 6, 1	0, 0, 0, 0, 0, 0, 0, 0, 0, 0, 0
619-99-8	-250.4	0.835, 0.884, 1.28, 1.21, 1.18	6, 3, 6, 2, 1	0, 0, 0, 0, 0, 0, 0, 0, 0, 0, 0
112-41-4	-226.2	2.02, 0.89, 1.46, 5.795, 4.98, 4.93	2, 3, 16, 1, 1, 1	0, 0, 0, 0, 0, 0, 0, 0, 0, 0, 0
112-40-3	-350.9	0.88, 1.27	6, 20	0, 0, 0, 0, 0, 0, 0, 0, 0, 0, 0
611-14-3	-46.4	7.22, 2.292, 2.61, 1.2	4, 3, 2, 3	0, 0, 0, 0, 0, 0, 0, 0, 0, 0, 0
620-14-4	-48.7	7.173, 7.01, 7, 6.99, 2.609, 2.329, 1.225	1, 1, 1, 1, 2, 3, 3	0, 0, 0, 0, 0, 0, 0, 0, 0, 0, 0
622-96-8	-49.8	7.08, 2.303, 2.59, 1.215	4, 3, 2, 3	0, 0, 0, 0, 0, 0, 0, 0, 0, 0, 0
142-82-5	-224.2	0.88, 1.3, 1.27	6, 4, 6	0, 0, 0, 0, 0, 0, 0, 0, 0, 0, 0
592-76-7	-97.9	2.05, 0.9, 1.6, 5.8, 4.93, 4.91	2, 3, 6, 1, 1, 1	0, 0, 0, 0, 0, 0, 0, 0, 0, 0, 0
14686-13-6	-109.5	5.66, 1.96, 1.63, 0.89, 1.53	2, 2, 3, 3, 4	0, 0, 0, 0, 0, 0, 0, 0, 0, 0, 0
14686-14-7	-109.3	5.41, 1.97, 0.9, 0.95, 1.36	2, 4, 3, 3, 2	0, 0, 0, 0, 0, 0, 0, 0, 0, 0, 0
544-76-3	-456.1	0.882, 1.26	6, 28	0, 0, 0, 0, 0, 0, 0, 0, 0, 0, 0

629-73-2	-328.7	2.03, 0.88, 1.26, 5.8, 4.97, 4.92	2, 3, 24, 1, 1, 1	0, 0, 0, 0, 0, 0, 0, 0, 0, 0, 0
592-42-7	54.1	2.151, 5.809, 4.95, 4.99	4, 2, 2, 2	0, 0, 0, 0, 0, 0, 0, 0, 0, 0, 0
110-54-3	-198.7	0.89, 1.29, 1.27	6, 4, 4	0, 0, 0, 0, 0, 0, 0, 0, 0, 0, 0
592-41-6	-74.2	2.06, 0.9, 1.5, 5.8, 4.96, 4.92	2, 3, 4, 1, 1, 1	0, 0, 0, 0, 0, 0, 0, 0, 0, 0, 0
4050-45-7	-85.5	5.44, 5.37, 1.93, 1.64, 0.89, 1.37	1, 1, 2, 3, 3, 2	0, 0, 0, 0, 0, 0, 0, 0, 0, 0, 0
7642_09_3	-78.9	5.33, 2.03, 0.961	2, 4, 6	0, 0, 0, 0, 0, 0, 0, 0, 0, 0, 0
71-43-2	49.1	7.339	6	0, 0, 0, 0, 0, 0, 0, 0, 0, 0, 0
1636-39-1	-178.9	1.127, 1.48, 1.577, 1.728	4, 4, 6, 4	0, 0, 0, 0, 0, 0, 0, 0, 0, 0, 0
104-51-8	-63.2	7.17, 7.18, 7.268, 2.606, 0.925, 1.355, 1.596	1, 2, 2, 2, 3, 2, 2	0, 0, 0, 0, 0, 0, 0, 0, 0, 0, 0
36383-15-0	-66.4	7.17, 7.28, 0.83, 1.24, 1.59, 2.58	3, 2, 3, 3, 2, 1	0, 0, 0, 0, 0, 0, 0, 0, 0, 0, 0
98-06-6	-71.9	7.37, 7.465, 7.815, 1.35	2, 1, 2, 9	0, 0, 0, 0, 0, 0, 0, 0, 0, 0, 0
98-19-1	-146.5	6.82, 7, 2.302, 1.295	1, 2, 6, 9	0, 0, 0, 0, 0, 0, 0, 0, 0, 0, 0
1075-38-3	-109.7	6.97, 7.14, 7.16, 7.18, 2.35, 1.31	1, 1, 1, 1, 3, 9	0, 0, 0, 0, 0, 0, 0, 0, 0, 0, 0
98-51-1	-109.7	7.11, 7.26, 2.309, 1.302	2, 2, 3, 9	0, 0, 0, 0, 0, 0, 0, 0, 0, 0, 0
110-82-7	-156.4	1.429	12	0, 0, 0, 0, 0, 0, 0, 0, 0, 0, 0
287-92-3	-105.1	1.504	10	0, 0, 0, 0, 0, 0, 0, 0, 0, 0, 0
124-18-5	-300.9	0.881, 1.26, 1.39	6, 12, 4	0, 0, 0, 0, 0, 0, 0, 0, 0, 0, 0
872-05-9	-173.8	2.03, 0.89, 1.28, 1.41, 5.79, 4.92, 4.98	2, 3, 12, 2, 1, 1, 1	0, 0, 0, 0, 0, 0, 0, 0, 0, 0, 0
75-83-2	-213.8	0.84, 0.855, 1.22	3, 9, 2	0, 0, 0, 0, 0, 0, 0, 0, 0, 0, 0
563-78-0	-93.2	1.703, 2.245, 1.023, 4.66	3, 1, 6, 2	0, 0, 0, 0, 0, 0, 0, 0, 0, 0, 0
558-37-2	-87.5	1.011, 5.834, 4.82, 4.926	9, 1, 1, 1	0, 0, 0, 0, 0, 0, 0, 0, 0, 0, 0
110-83-8	-38.5	5.661, 1.98, 1.61	2, 4, 4	0, 0, 0, 0, 0, 0, 0, 0, 0, 0, 0
92-51-3	-273.7	1.68, 1.17, 0.98	10, 6, 6	0, 0, 0, 0, 0, 0, 0, 0, 0, 0, 0
1678-93-9	-263.1	0.88, 1.33, 1.74, 1.64, 1.33, 1.13, 0.87	3, 6, 4, 1, 3, 1, 2	0, 0, 0, 0, 0, 0, 0, 0, 0, 0, 0
590-66-9	-218.7	0.876, 1.435, 1.24	6, 6, 4	0, 0, 0, 0, 0, 0, 0, 0, 0, 0, 0
6876-23-9	-218.2	0.89, 1.47, 1.78	6, 6, 4	0, 0, 0, 0, 0, 0, 0, 0, 0, 0, 0
624-29-3	-215.6	0.906, 1.19, 1.4, 1.59	6, 4, 2, 4	0, 0, 0, 0, 0, 0, 0, 0, 0, 0, 0
2207_01_4	-211.8	0.83, 1.88, 1.46	6, 5, 5	0, 0, 0, 0, 0, 0, 0, 0, 0, 0, 0
1003_64_1	-103.5	2.17, 2.1, 1.51, 1.575, 5.17	2, 2, 3, 6, 1	0, 0, 0, 0, 0, 0, 0, 0, 0, 0, 0
827-52-1	-76.6	7.47, 2.47, 1.6, 2.08	5, 1, 5, 5	0, 0, 0, 0, 0, 0, 0, 0, 0, 0, 0
2040-96-2	-188.8	0.89, 1.28, 1.74, 1.6, 1.51, 1.06	3, 4, 3, 2, 2, 2	0, 0, 0, 0, 0, 0, 0, 0, 0, 0, 0
3524-75-2	-64.5	2.052, 1.858, 1.733, 1.59, 1.51, 1.136, 5.8, 4.983, 4.928	2, 1, 2, 2, 2, 2, 1, 1, 1	0, 0, 0, 0, 0, 0, 0, 0, 0, 0, 0
1638-26-2	-172.1	0.96, 1.475	6, 8	0, 0, 0, 0, 0, 0, 0, 0, 0, 0, 0
13269-52-8	-86.1	5.43, 2, 0.79	2, 4, 6	0, 0, 0, 0, 0, 0, 0, 0, 0, 0, 0
7688-21-3	-83.9	5.38, 5.44, 1.53, 1.98, 0.87, 1.33	1, 1, 3, 2, 3, 2	0, 0, 0, 0, 0, 0, 0, 0, 0, 0, 0
104-72-3	-218.3	7.47, 2.58, 0.88, 1.6, 1.26	5, 2, 3, 2, 14	0, 0, 0, 0, 0, 0, 0, 0, 0, 0, 0

563-79-1	-101.4	1.64	12	0, 0, 0, 0, 0, 0, 0, 0, 0, 0, 0
142-29-0	4.3	5.73, 2.3, 1.82	2, 4, 2	0, 0, 0, 0, 0, 0, 0, 0, 0, 0, 0
106-98-9	-20.8	2.02, 0.98, 4.92, 5.84	2, 3, 1, 2	0, 0, 0, 0, 0, 0, 0, 0, 0, 0, 0
760-20-3	-78.2	2.1, 0.98, 0.88, 1.29, 5.7, 4.95, 4.9	1, 3, 3, 2, 1, 1, 1	0, 0, 0, 0, 0, 0, 0, 0, 0, 0, 0
100-42-5	103.8	7.3, 6.692, 5.225, 5.737	5, 1, 1, 1	0, 0, 0, 0, 0, 0, 0, 0, 0, 0, 0
593-45-3	-505.2	0.88, 1.26	6, 32	0, 0, 0, 0, 0, 0, 0, 0, 0, 0, 0

Table S3. Database used in the paper.

S4 - Comparison of Descriptor Vector Combinations:

Table S4 summarizes the predictive performance of different descriptor combinations for the complete dataset (Model 3 – 364 organic molecules). For this comparison, the analyzed ^{1}H NMR spectral region was defined from 0.0 to 12.0 ppm, with a bin width of 0.5 ppm.

Due to the nature of NMR-based descriptors, some bins contain zero values because not all compounds exhibit signals across the entire spectral range and certain regions of the spectrum are typically signal-free. Nevertheless, the total number of predictors remains consistent across descriptor constructions.

As shown in Table S4, models based solely on spectral descriptors or heteroatom motif descriptors individually exhibit significantly lower predictive performance. In contrast, combining spectral information with heteroatom motif descriptors leads to a substantial improvement in accuracy. Among the tested variants, the ISV + Exists descriptor combination achieved the best balance between predictive accuracy and model simplicity, yielding a 30× repeated 10-fold CV R^2 of 0.989 ± 0.001 while requiring fewer predictors than more complex descriptor combinations.

The influence of bin width and analyzed spectral range on the ISV + Exists descriptor combination is further examined in Section S5 and S6.

Descriptor Combination	Number of Predictors	Optimal PLS Components Based on Nested CV	Calibration R^2	30× 10-Fold CV R^2	30× 10-Fold CV RMSE (kJ mol^{-1})	30× 10-Fold CV MAE (kJ mol^{-1})
ISV+Exists	35	9 [range 6-10]	0.9941	0.989 ± 0.001	23.4 ± 0.7	13.9 ± 0.4
MSV+ISV +Exists	59	10 [range 8-38]	0.9921	0.982 ± 0.001	29.0 ± 0.8	16.3 ± 0.5
WSV+Exists	35	9 [range 8-26]	0.9936	0.988 ± 0.001	24 ± 1	15.3 ± 0.3
WSV+ISV +Exists	59	11 [range 8-27]	0.9934	0.985± 0.001	26.5 ± 0.7	14.7 ± 0.4
ISV	24	3 [range 1-5]	0.778	0.74 ± 0.01	112 ± 2	87 ± 1.3
MSV+ISV	48	4 [range 2-16]	0.812	0.769 ± 0.008	105.1 ± 1.8	79.3 ± 1.3
WSV	24	3 [range 1-5]	0.777	0.738 ± 0.009	111.8 ± 1.8	86.9 ± 1.3
WSV+ISV	48	3 [range 1-2]	0.778	0.74 ± 0.01	112 ± 2	86.7 ± 1.3
Exists	11	2 [range 2-3]	0.758	0.738 ± 0.004	112.0 ± 0.8	89.1 ± 0.5

Table S4. Comparison of predictive performance for PLS models constructed using different descriptor vector combinations.

S5 - Comparison of Analyzed NMR Area:

As shown in Table S5, the optimal spectral window for analysis was determined to be 0–10.5 ppm, based on results obtained using Model III with a bin width of 0.5 ppm based on ISV + Exists descriptor combination. Although the dataset contains signals extending to approximately 12.0 ppm, these correspond to carboxylic acid O–H. Such signals are known to be highly sensitive to experimental conditions, including hydrogen bonding, concentration, and exchange processes, often resulting in broad, variable, and less reproducible chemical shifts. Consequently, they are less suitable as reliable descriptors for feature construction. In the present dataset, the most upfield carboxylic acid signal occurs slightly above 10.5 ppm, while all other proton resonances fall within the 0–10.5 ppm range. This window therefore retains chemically informative signals while excluding condition dependent contributions, providing a balanced and robust basis for descriptor construction.

Analyzed spectral window	Number of Predictors	Optimal PLS Components	Calibration R^2	30× 10-Fold CV R^2	30× 10-Fold CV RMSE (kJ mol⁻¹)	30× 10-Fold CV MAE (kJ mol⁻¹)
0-10.5 ppm	32	9 [range 8-13]	0.9949	0.9915 ± 0.0004	20.1 ± 0.4	12.9 ± 0.3
0-12.0 ppm	35	9 [range 6-10]	0.9941	0.989 ± 0.001	23.4 ± 0.7	13.9 ± 0.4

Table S5: Influence of NMR spectral window on the predictive performance of the ISV + Exists descriptor combination in model 3.

S6 - Comparison of Different Bin widths:

Tables S6–S9 summarize the predictive performance obtained for different bin widths across Models 1–3. For this comparison, the previously established ISV + Exists descriptor combination was employed, while the analyzed ^{1}H NMR spectral region was defined from 0.0 to 10.5 ppm. Due to the nature of NMR-based descriptors, some bins contain zero values because not all compounds exhibit signals across the entire spectral range and certain regions of the spectrum are typically signal-free.

As shown in Tables S6–S9, several bin widths yield comparably strong predictive performance. In particular, 0.5, 0.75, and 1.5 ppm consistently provide high cross-validated accuracy and low prediction errors across the tested models. Among these candidates, a bin width of 0.75 ppm was selected for the models presented in this work. This choice reflects its consistently strong performance across the evaluated benchmarks, relatively low standard deviation values in repeated validation tests, and a moderate number of predictors compared with the finer binning scheme of 0.5 ppm.

It is important to note, however, that the results indicate a broad performance plateau across the tested bin widths. Alternative values, particularly 0.5 and 1.5 ppm, also produce highly accurate models and could reasonably be employed without significant loss of predictive performance. The use of PLS latent variables further mitigates the risk of overfitting despite variations in descriptor dimensionality.

Bin Width	Number of Predictors	Optimal PLS Components	Calibration R^2	30× 10-Fold CV R^2	30× 10-Fold CV RMSE (kJ mol^{-1})	30× 10-Fold CV MAE (kJ mol^{-1})
0.25 ppm	53	11 [range 7-13]	0.9952	0.990 ± 0.001	22.1 ± 0.5	14.3 ± 0.3
0.5 ppm	32	9 [range 8-13]	0.9949	0.9915 ± 0.0004	20.1 ± 0.4	12.9 ± 0.3
0.75 ppm	25	8 [range 7-10]	0.9937	0.9891 ± 0.0007	22.8 ± 0.7	14.0 ± 0.3
1.5 ppm	18	8 [range 7-9]	0.9934	0.9905 ± 0.0003	21.3 ± 0.4	14.1 ± 0.3
1.75 ppm	17	6 [range 5-8]	0.9890	0.9867 ± 0.0004	25.3 ± 0.4	18.3 ± 0.3

Table S6: Influence of NMR bin width on the predictive performance of the ISV + Exists descriptor combination in model 3.

Bin Width	Number of Predictors	Optimal PLS Components	Calibration R^2	30× 10-Fold CV R^2	30× 10-Fold CV RMSE (kJ mol^{-1})	30× 10-Fold CV MAE (kJ mol^{-1})
0.25 ppm	48	14 [range 12-22]	0.9972	0.9946 ± 0.0004	16.1 ± 0.5	10.2 ± 0.2
0.5 ppm	27	10 [range 9-12]	0.9963	0.9940 ± 0.0005	16.8 ± 0.7	10.8 ± 0.3
0.75 ppm	20	10 [range 9-15]	0.9958	0.9945 ± 0.0002	16.2 ± 0.3	10.9 ± 0.2
1.5 ppm	13	8 [range 8-10]	0.9948	0.9942 ± 0.0002	16.7 ± 0.2	11.5 ± 0.2
1.75 ppm	12	7 [range 6-7]	0.9915	0.9904 ± 0.0002	21.3 ± 0.2	16.4 ± 0.2

Table S7: Influence of NMR bin width on the predictive performance of the ISV + Exists descriptor combination in model 2.

Bin Width	Number of Predictors	Optimal PLS Components	Calibration R^2	30× 10-Fold CV R^2	30× 10-Fold CV RMSE (kJ mol^{-1})	30× 10-Fold CV MAE (kJ mol^{-1})
0.25 ppm	42	8 [range 5-12]	0.9899	0.976 ± 0.005	16.9 ± 1.5	11.0 ± 0.6
0.5 ppm	21	6 [range 5-8]	0.9852	0.974 ± 0.004	17.8 ± 1.3	11.5 ± 0.6
0.75 ppm	14	5 [range 4-8]	0.9845	0.981 ± 0.001	15.2 ± 0.5	10.5 ± 0.3
1.5 ppm	7	4 [range 4-5]	0.9788	0.977 ± 0.001	16.8 ± 0.4	11.9 ± 0.2
1.75 ppm	6	4 [range 3-5]	0.9703	0.967 ± 0.001	19.8 ± 0.4	14.2 ± 0.3

Table S8: Influence of NMR bin width on the predictive performance of the ISV + Exists descriptor combination in model 1.

Model type	Bin Width	Number of Predictors	Optimal PLS Components	30x 75/25 external test R^2	30x 75/25 external test RMSE (kJ mol^{-1})	30x 75/25 external test MAE (kJ mol^{-1})
Model 1	0.5	21	6 [range 5-7]	0.97 ± 0.01	18.1 ± 3.3	12 ± 2
	0.75	14	5 [range 4-8]	0.980 ± 0.009	14.7 ± 2.9	10 ± 2
	1.5	7	4 [range 4-4]	0.975 ± 0.009	16.6 ± 2.9	12 ± 2
Model 2	0.5	27	10 [range 9-12]	0.994 ± 0.003	16.7 ± 3.2	11.0 ± 1.6
	0.75	20	10 [range 9-11]	0.994 ± 0.002	16.3 ± 2.2	11.2 ± 1.3
	1.5	13	8 [range 8-10]	0.994 ± 0.002	16.6 ± 2.1	11.6 ± 1.4
Model 3	0.5	32	9 [range 7-10]	0.990 ± 0.003	20.7 ± 3.2	13.6 ± 1.6
	0.75	25	8 [range 7-9]	0.987 ± 0.007	22.7 ± 5.7	14.4 ± 2.2
	1.5	18	7 [range 7-8]	0.990 ± 0.004	20.9 ± 3.7	14.0 ± 1.6

Table S9: Influence of NMR bin width on the predictive performance of the ISV + Exists descriptor combination in model 1-3 in the repeated 75/25 external test.

S7 - Correlation Plots for Individual Models

The following figures present the correlation (parity) plots obtained for Models 1–3. In all cases, the bin width was set to 0.75 ppm, and the analyzed ^{1}H NMR spectral region was defined from 0.0 to 10.5 ppm.

For models employing HNMR bin-based descriptors coupled with heteroatom-based motif correction (Models 2 and 3), additional correlation plots based solely on the HNMR bin descriptors without the motif correction are included for comparison.

Blue solid markers correspond to training-set predictions, while orange open markers represent results obtained from repeated nested 10-fold cross-validation. The dashed line (y = x) denotes perfect agreement between predicted and experimental values.

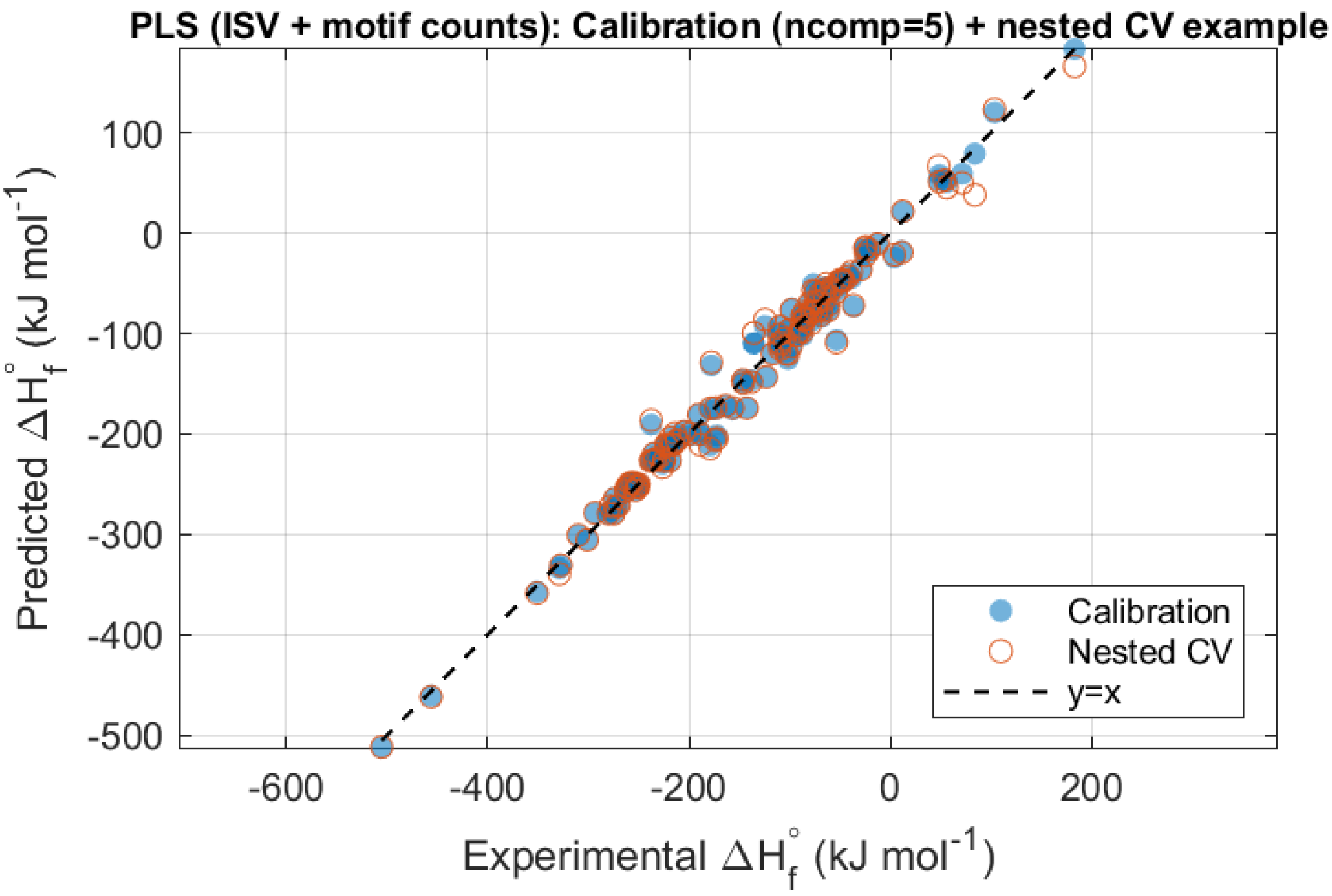

Figure S6. Correlation (parity) plot for Model 1, describing hydrocarbons using binned ^{1}H NMR spectral descriptors only.

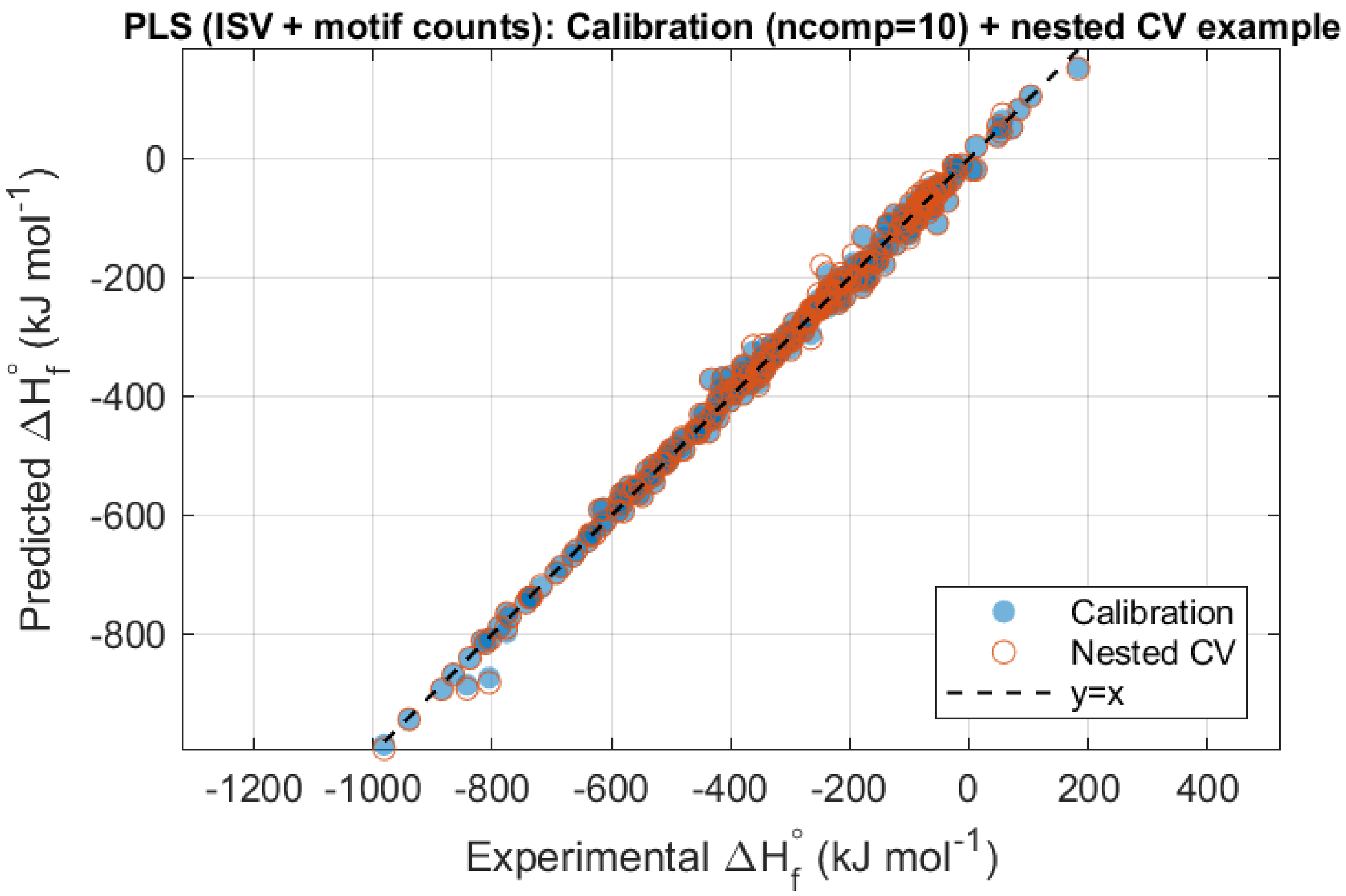

Figure S7. Correlation (parity) plot for Model 2, employing binned ^{1}H NMR spectral descriptors combined with heteroatom motif correction.

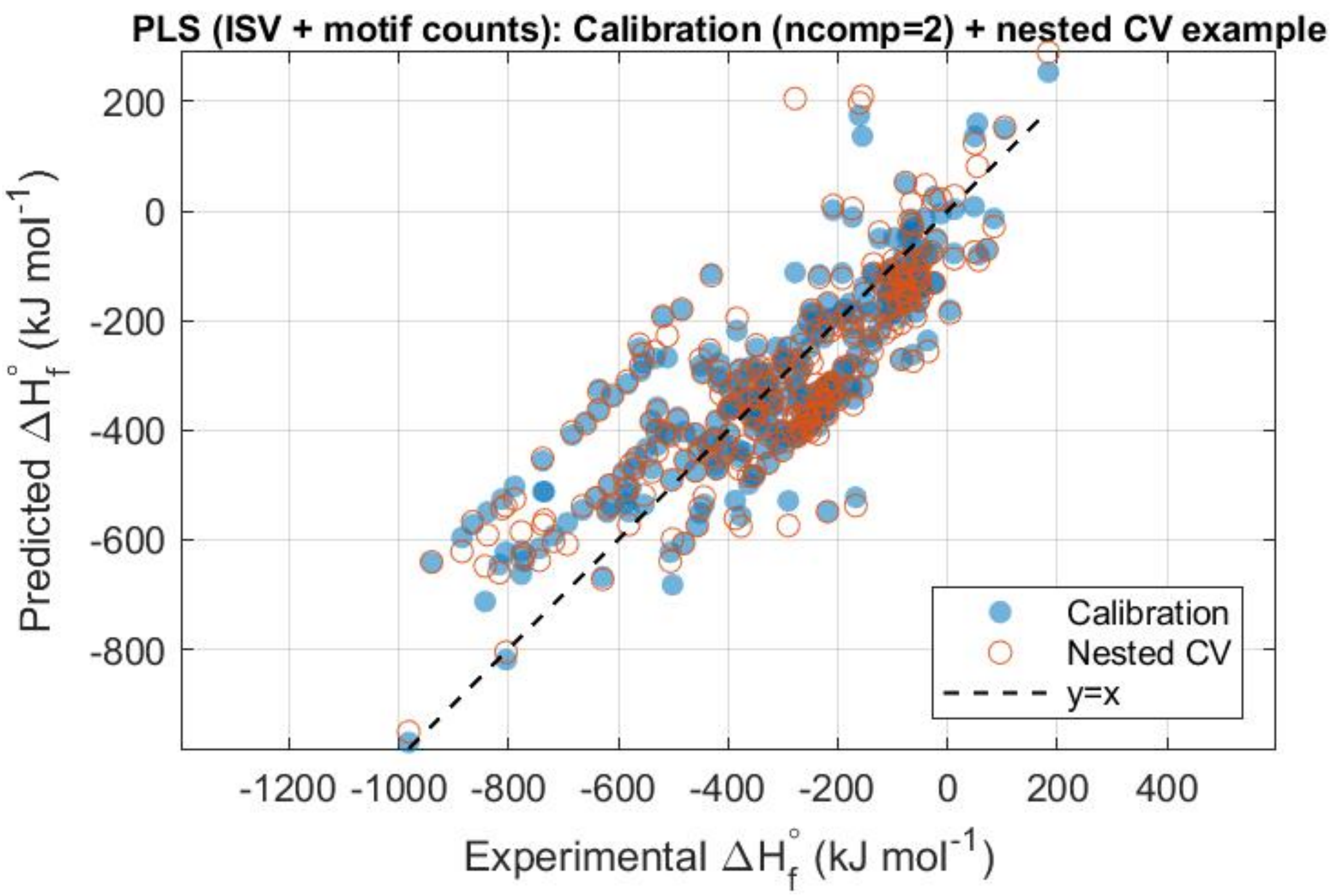

Figure S8: Correlation (parity) plot for Model 2 using binned ^{1}H NMR spectral descriptors only, shown for comparison with the heteroatom-corrected model.

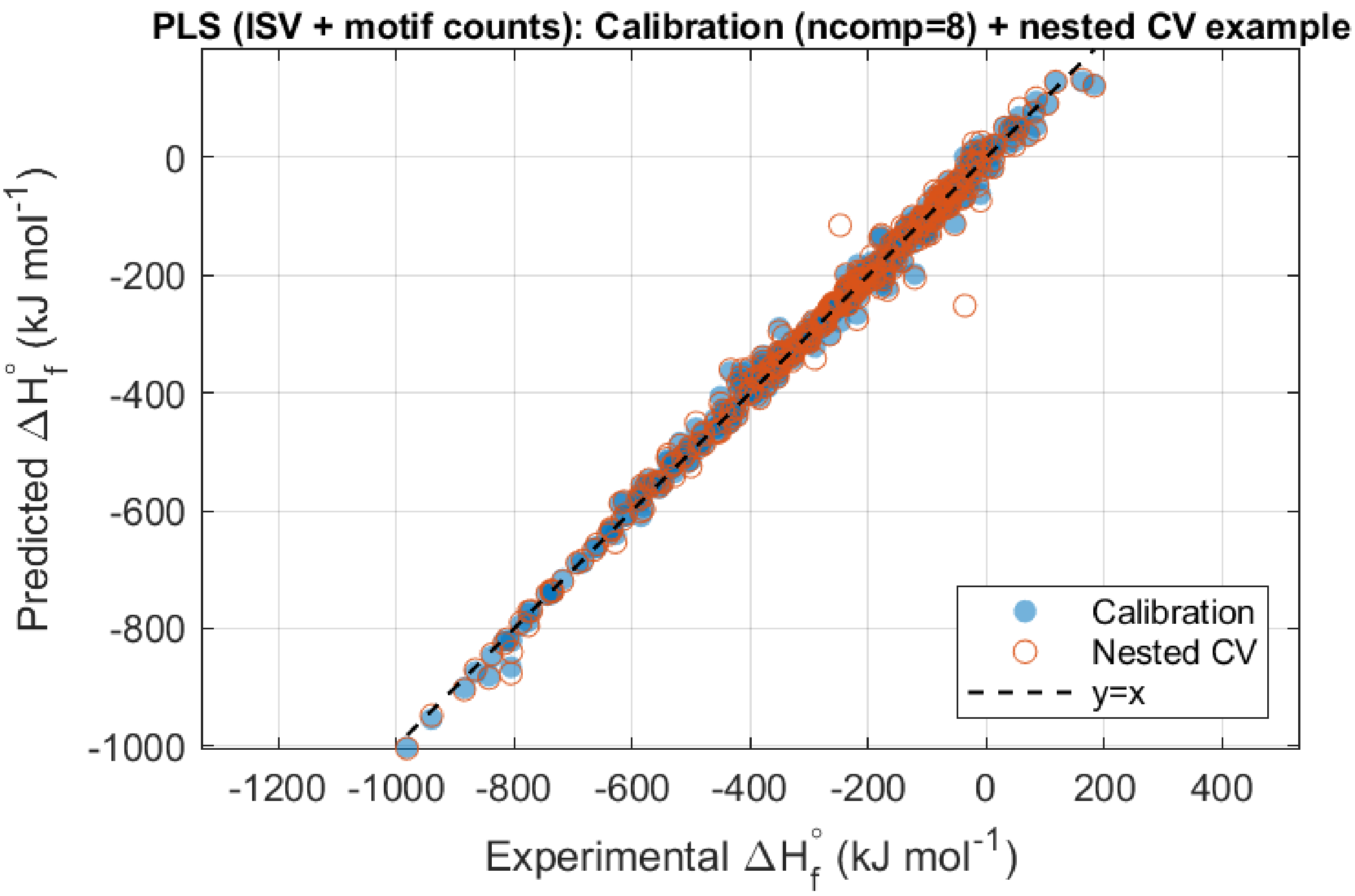

Figure S9. Correlation (parity) plot for Model 3, employing binned ^{1}H NMR spectral descriptors combined with heteroatom motif correction.

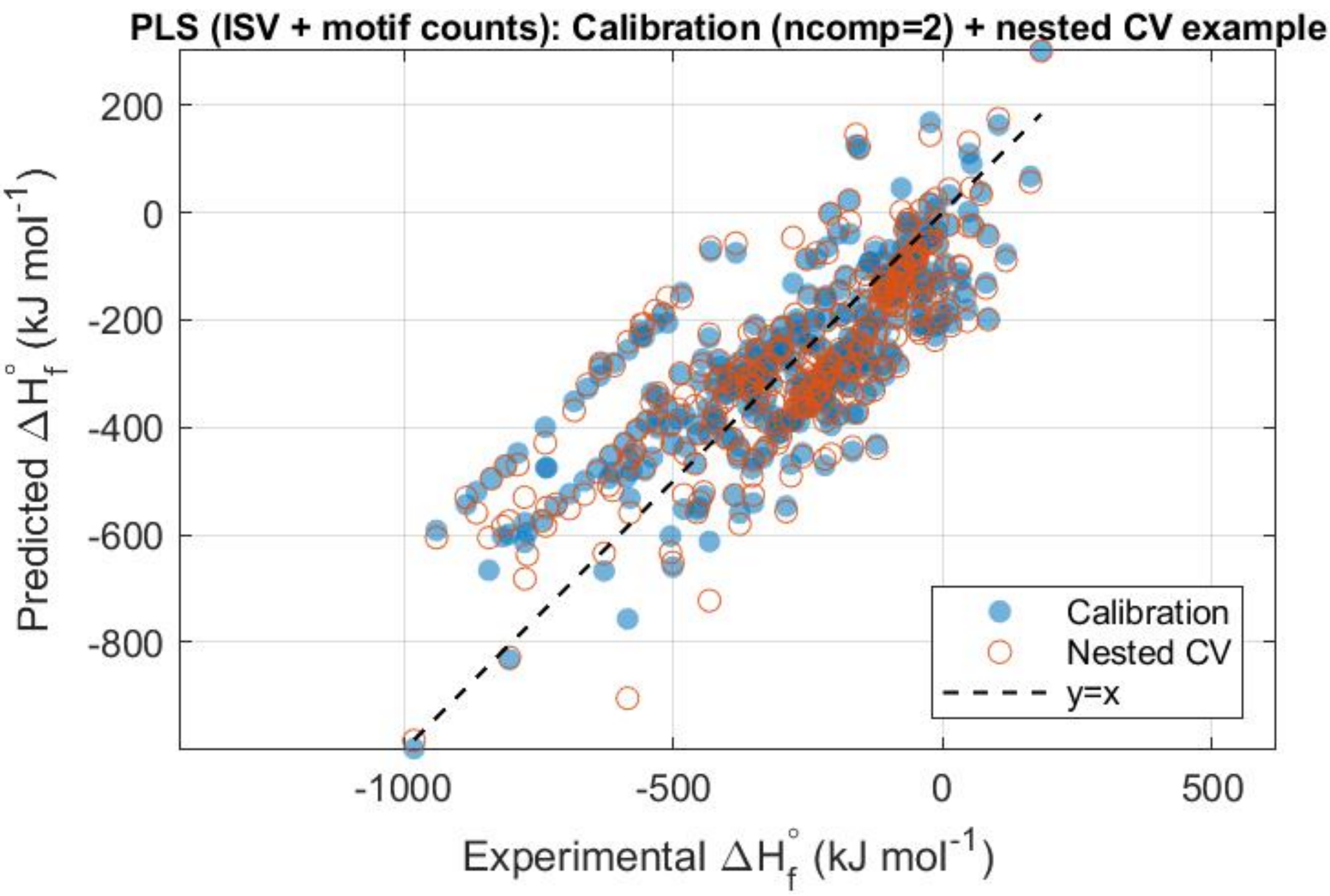

Figure S10. Correlation (parity) plot for Model 3 using binned ^{1}H NMR spectral descriptors only, shown for comparison with the heteroatom-corrected model.

S8 - Permutation Tests (Y-scrambling)

Y-permutation tests for the nested cross-validation of Models 1–3 are presented. A total of 500 permutations were performed. For each model, the corresponding p-value, average performance metrics obtained from the permutations, and the distribution histogram are reported. Table S9 summarizes the results of the Y-permutation tests performed for Models 1–3. In all cases, the predictive performance of the original models is substantially higher than that obtained for the scrambled models, with empirical p-values of approximately 0.002, indicating that the observed correlations are unlikely to arise from chance.

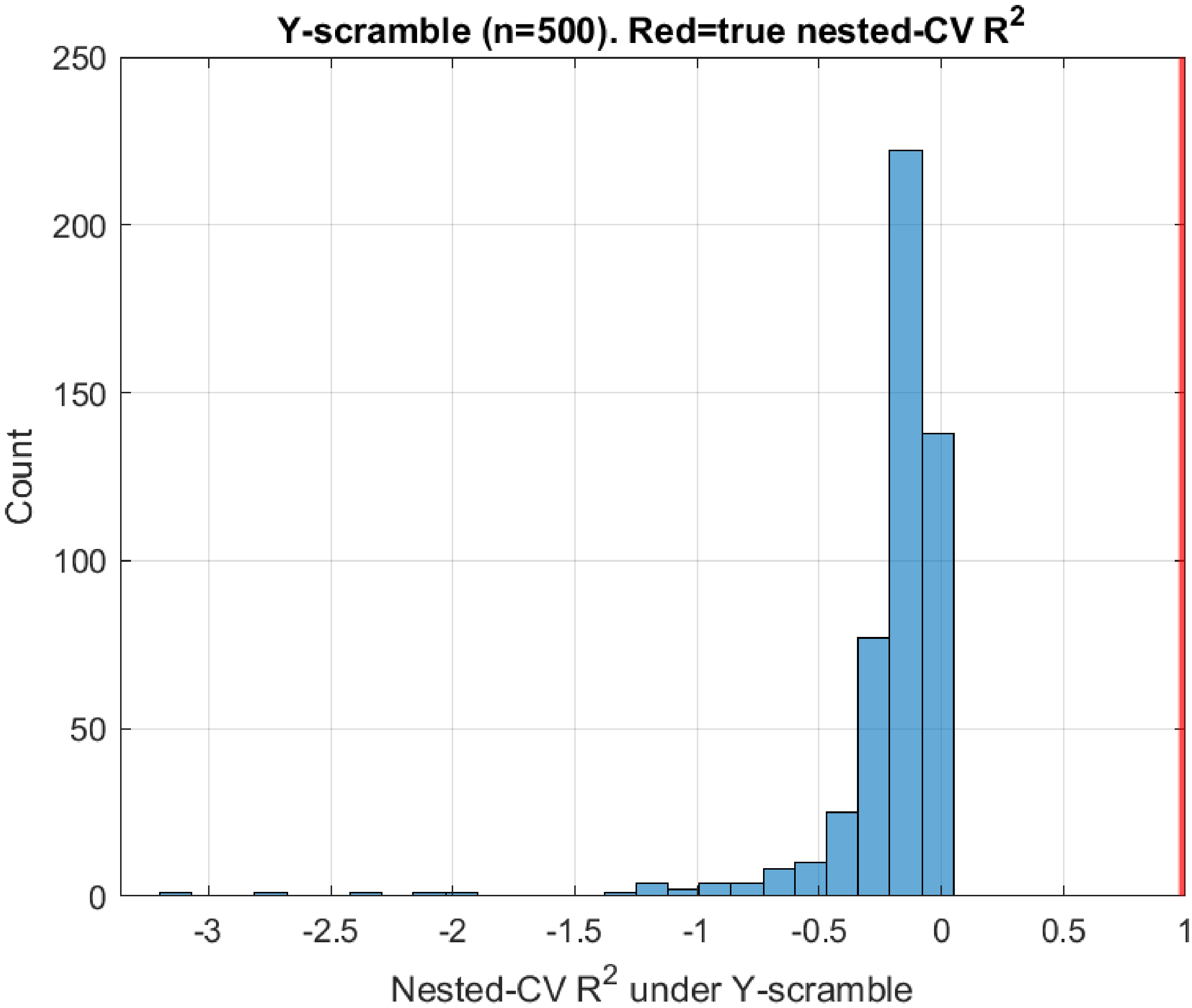

Figure S11. Distribution of R^2 values obtained from 500 Y-permutations of Model 1 (binned ^1H NMR descriptors). The red line indicates the R^2 value from the 30× repeated nested cross-validation of the original model.

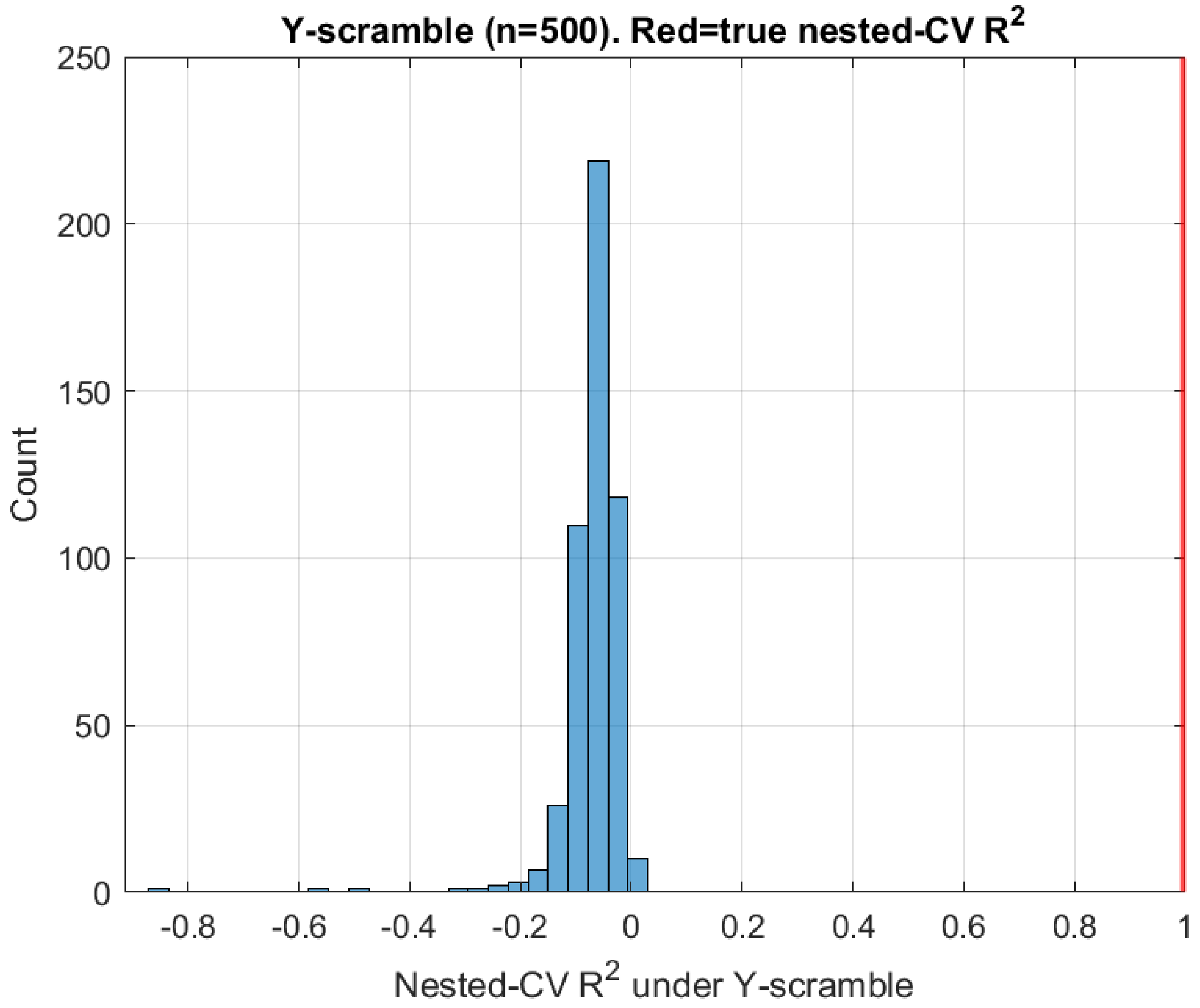

Figure S12. Distribution of R^2 values obtained from 500 Y-permutations of Model 2 (binned ^1H NMR descriptors + Hetero atom motif corrections). The red line indicates the R^2 value from the 30× repeated nested cross-validation of the original model.

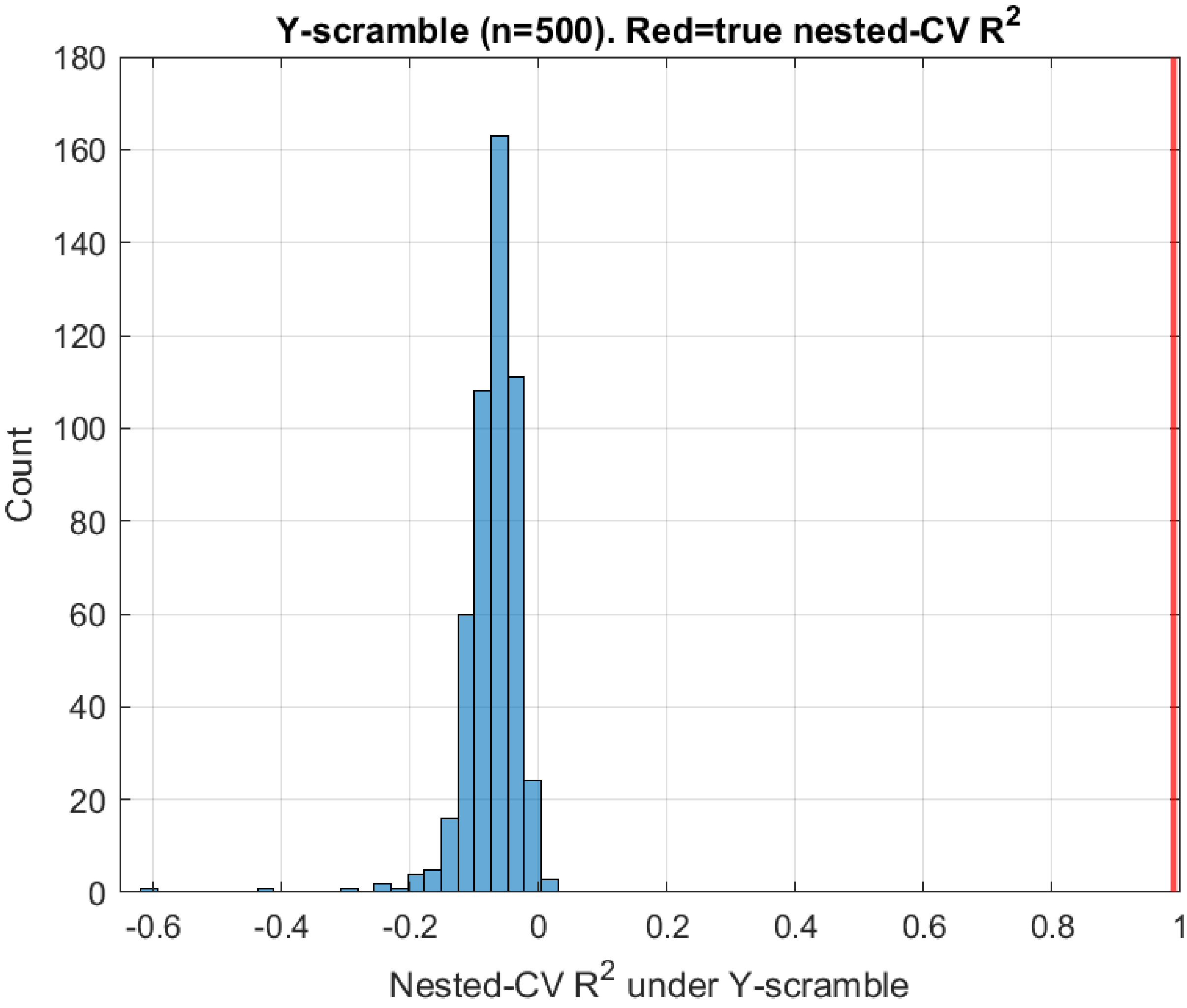

Figure S13. Figure S13. Distribution of R^2 values obtained from 500 Y-permutations of Model 3 (binned ^{1}H NMR descriptors + Hetero atom motif corrections). The red line indicates the R^2 value from the 30× repeated nested cross-validation of the original model.

Model	Nested CV R^2 (True Model)	Scrambled R^2 (mean ± SD)	Range	Empirical P value
Model 1	0.9808	-0.210 ± 0.299	−3.123 – 0.032	$p < 0.002$
Model 2	0.9945	-0.069 ± 0.060	−0.852 – 0.025	$p < 0.002$
Model 3	0.9891	-0.072 ± 0.047	−0.611 – 0.014	$p < 0.002$

Table S9. Results of Y-permutation tests for Models 1–3 using repeated nested cross-validation

S9 - Comparison to Benson

For the 42-molecule benchmark subset, Benson-based liquid-phase standard enthalpies of formation were obtained by first estimating the gas-phase values using the Benson group additivity method [64] and then converting them to the liquid phase using experimental enthalpies of vaporization from NIST [65] according to $\Delta H_f^\circ(l) = \Delta H_f^\circ(g) - \Delta H_{vap}$.

The molecules included in this comparison fall within both the chemical scope of the present model and the applicability domain of the original Benson increment scheme. Molecules containing cyano or nitro substituents were excluded from the comparison because such functional groups are outside the scope of the Benson parameterization used in this work.

Two representative calculations are provided below as illustrative examples. The resulting Benson-derived liquid-phase enthalpies of formation used for the benchmark comparison are listed in Table S11. And Table S10 compares the predictive performance of the Benson group additivity method and the NMR-PLS model for the same benchmark subset of 42 molecules.

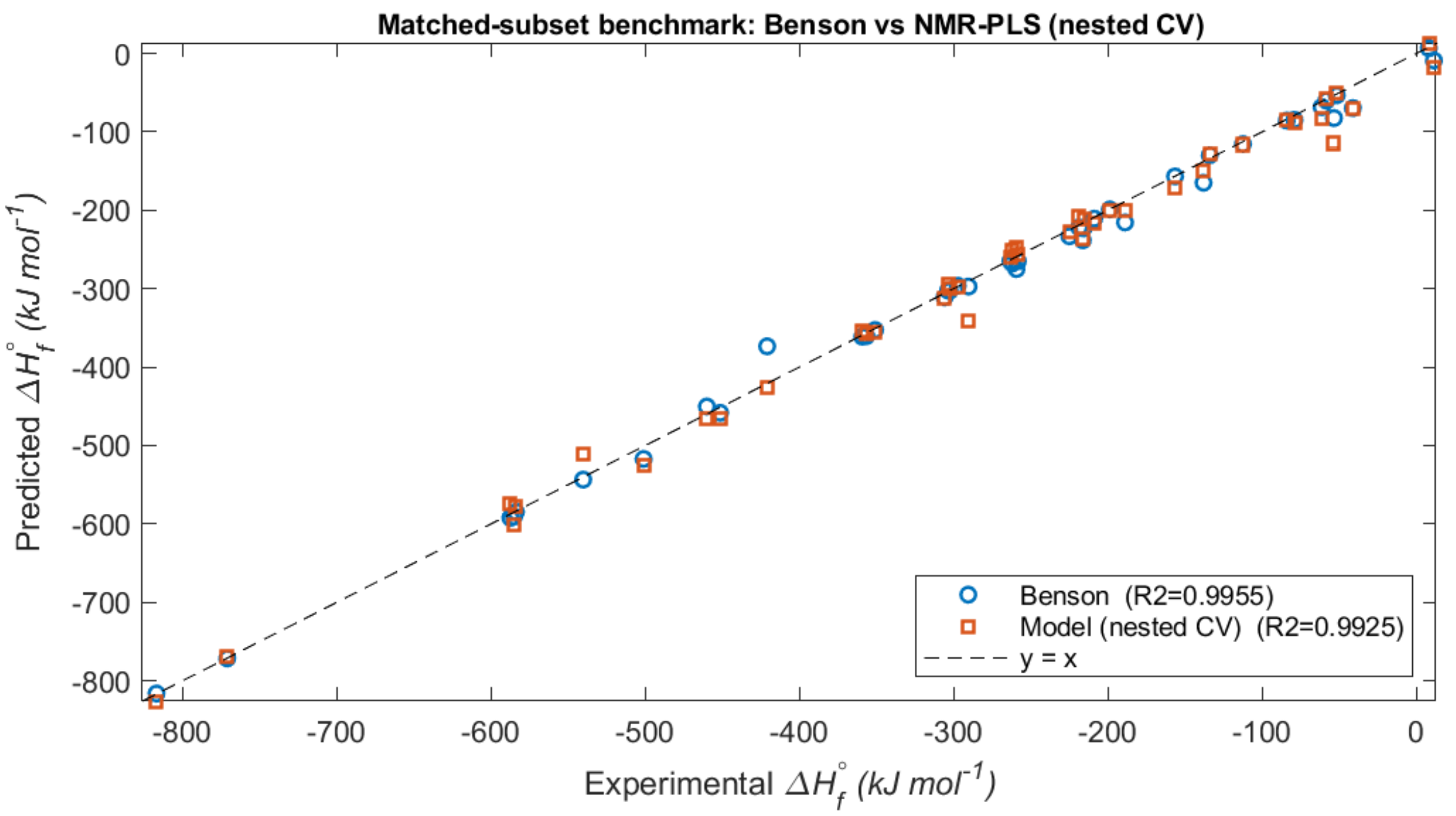

Figure S14: Comparison of predictive performance on a matched subset of the dataset between the Benson group additivity method and NMR–PLS predictions (Model III, nested CV example).

Example 1: 91-16-7

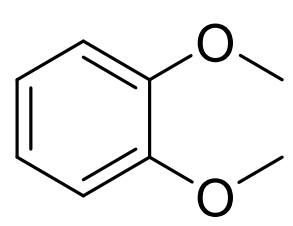

$\Delta H_f^\circ(g) =$ 4*C_B-(H) + 2*C_B-(O) + 2*O-(C_B)(C) + 2*C-(O)$(H)_3$ = 4.184 kJ/kcal* ((4*3.30 kcal/mol + 2*(-0.9 kcal/mol) +2*(-23.0 kcal/mol) +2*(-10.08 kcal/mol)) = -229.12 kj/mol

$\Delta H_f^\circ(l) = \Delta H_f^\circ(g) - \Delta H_{vap}$ = -229.12 kj/mol – 68.1 kj/mol = **-297.22 kj/mol**

Example 2: 75-31-0

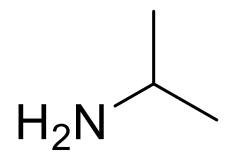

$\Delta H_f^{\circ}(g) =$ 2*C-(C)(H)3 + C-(N)(C)2(H) + N-(C)(H)2 = 4.184 kJ/kcal* (2*(-10.20 kcal/mol) - 5.2 kcal/mol +4.8 kcal/mol) = 83.68 kj/mol

$\Delta H_f^{\circ}(l) = \Delta H_f^{\circ}(g) - \Delta H_{vap}$ = -87.03 kj/mol – 28.5 kj/mol = **115.5 kj/mol**

Method	R^2	RMSE (kJ mol^{-1})	MAE (kJ mol^{-1})
Benson group additivity	0.9955	13.27	8.33
NMR-PLS model (nested CV example)	0.9925	17.23	11.65
NMR-PLS model (training-reference)	0.9943	14.96	10.10

Table S10. Comparison of prediction accuracy between the Benson group additivity method and the NMR-PLS model for the benchmark subset of 42 molecules.

#	CAS RN	Final $\Delta H^{\circ}_{f_{\text{liquid}}}$ (Kj mol^{-1})
1	496-11-7	-8.9
2	107-88-0	-517.06
3	142-77-8	-816.3
4	2207-03-6	-223
5	90-00-6	-210.67
6	75-31-0	-115.52
7	563-45-1	-53.3
8	75-65-0	-361.16
9	7132-64-1	-771.5
10	106-70-7	-543.5
11	1192-37-6	-68.4
12	107-87-9	-296.3
13	71-23-8	-302.76
14	540-84-1	-274.97
15	142-62-1	-584.62
16	629-14-1	-458.25
17	590-73-8	-267.8
18	91-16-7	-297.22
19	2146-38-5	-82.35
20	617-78-7	-233.06
21	107-21-1	-450.23
22	628-32-0	-302.23
23	110-54-3	-198.86
24	7642-09-3	-84.6
25	123-75-1	-69.65
26	7688-21-3	-85.52
27	34713-94-5	-360.49
28	96-37-7	-164.83

29	109-99-9	-238.2
30	96-48-0	-373.47
31	2040-96-2	-215.49
32	142-68-7	-264.93
33	110-82-7	-156.76
34	97-85-8	-592.19
35	646-04-8	-60.09
36	112-40-3	-352.62
37	1678-93-9	-264.64
38	811-93-8	-129.69
39	110-96-3	-222.0
40	103-69-5	6.69
41	1116-76-3	-590.53
42	100-37-8	-311.07

Table S11. Liquid-phase formation enthalpies obtained from Benson gas-phase estimates combined with experimental vaporization enthalpies.

S10 - Variable Importance in Projection (VIP) Plots

Variable Importance in Projection (VIP) scores are presented for each model. In addition to the individual predictor VIP values, the mean VIP and the share of total VIP contribution for each descriptor block are also reported. The VIP plots illustrate the relative importance of the original predictors within the latent PLS components, reflecting their contribution to the explained variance of the response variable (ΔH°f). Across all models, the block of binned ^{1}H NMR descriptors consistently contributes slightly more to the predictive information than the heteroatom motif (exists) block, indicating that the spectral descriptors provide the primary source of information for predicting formation enthalpies.

Model 1, which uses only binned ^{1}H NMR descriptors, assigns the entire predictive contribution to the spectral descriptor block, with a mean VIP value of 0.722 accounting for 100% of the total VIP importance.

In Model 2, the ISV block exhibits a mean VIP of 0.723, contributing 57.7% of the total VIP importance, while the motif (exists) block shows a mean VIP of 1.017, accounting for 42.3%.

For Model 3, the contributions are more balanced: the ISV block has a mean VIP of 0.760 (50.0%), while the motif block has a mean VIP of 0.887 (50.0%).

Overall, the VIP analysis indicates that spectral descriptors provide the dominant predictive information, while motif descriptors contribute complementary structural corrections.

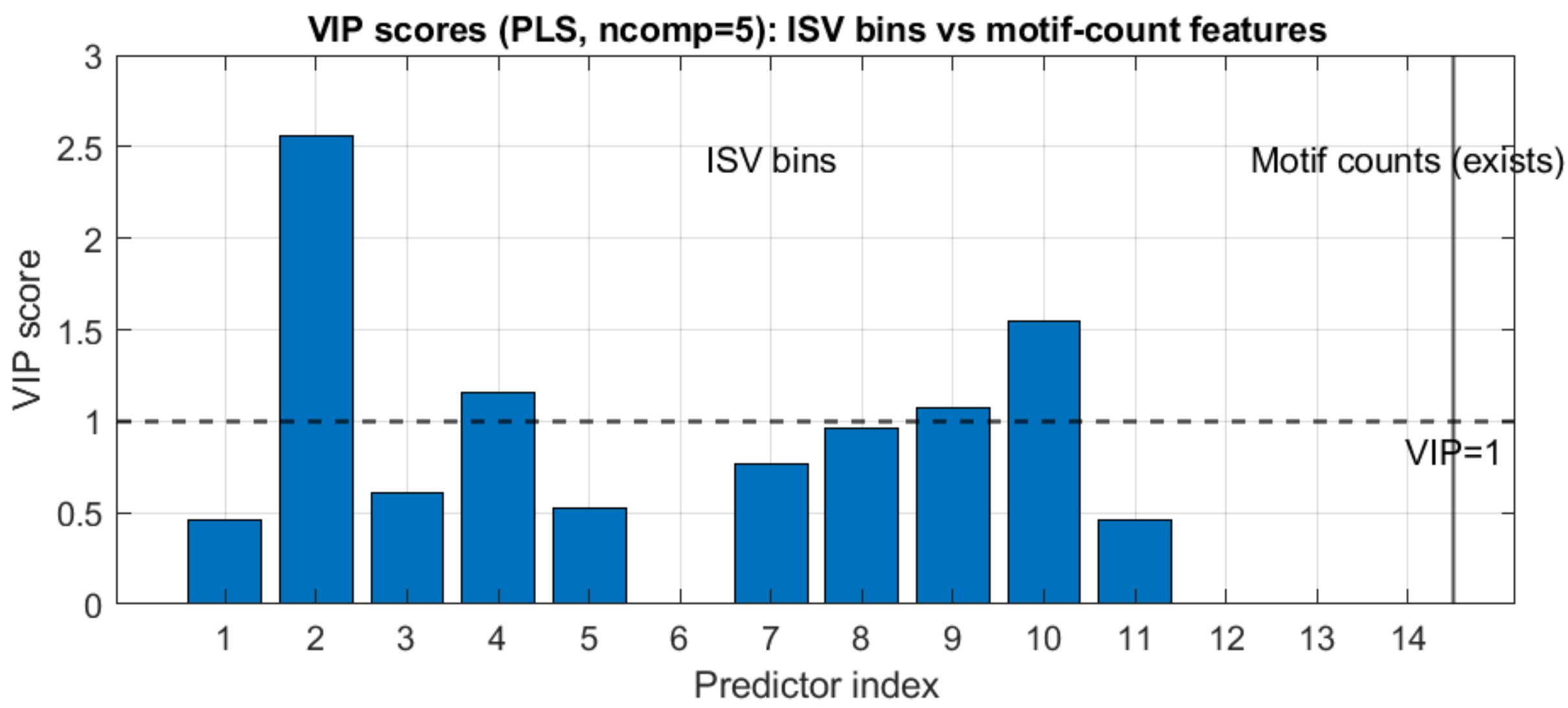

Figure S15. Variable Importance in Projection (VIP) scores for Model 1 using binned ^{1}H NMR descriptors.

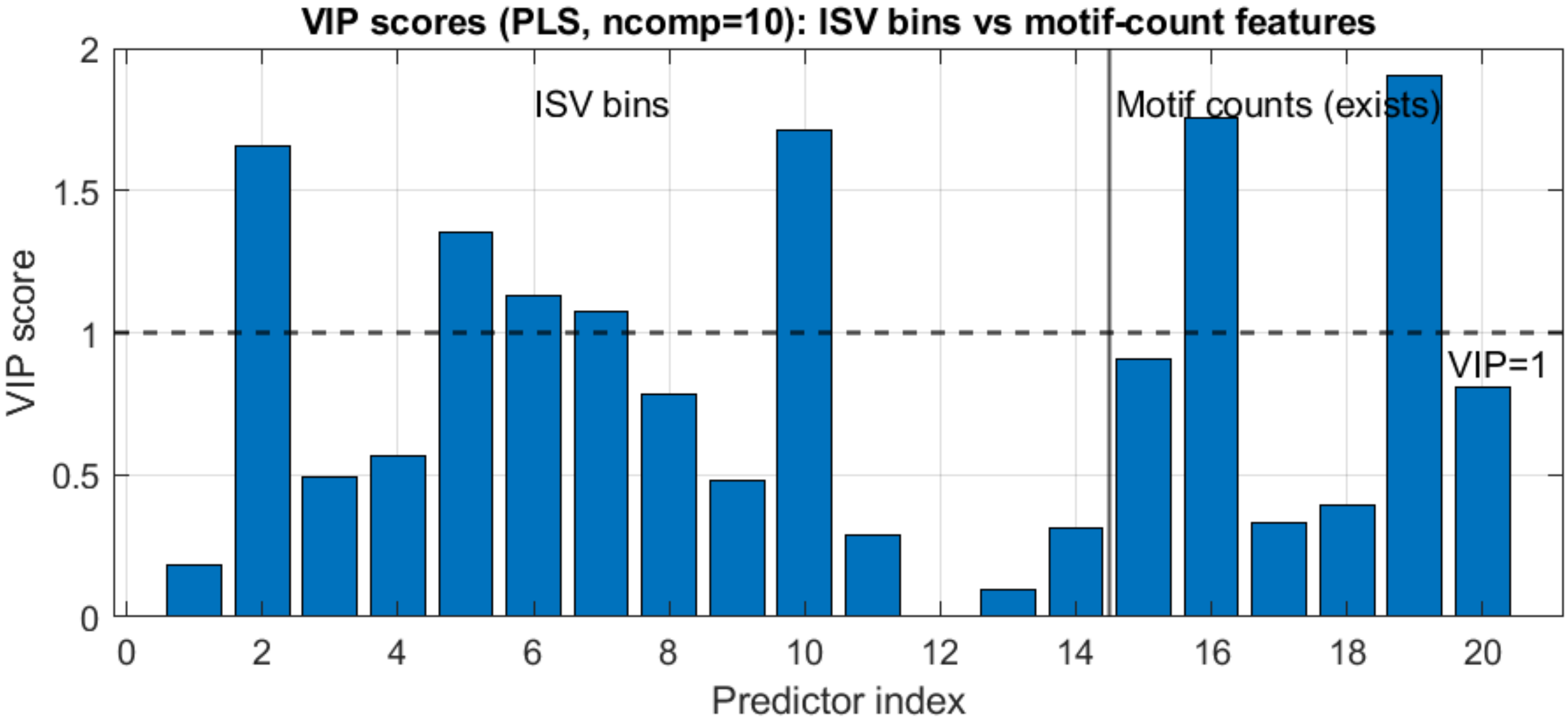

Figure S16. Variable Importance in Projection (VIP) scores for Model 2 using binned ^1H NMR descriptors and heteroatom motif corrections.

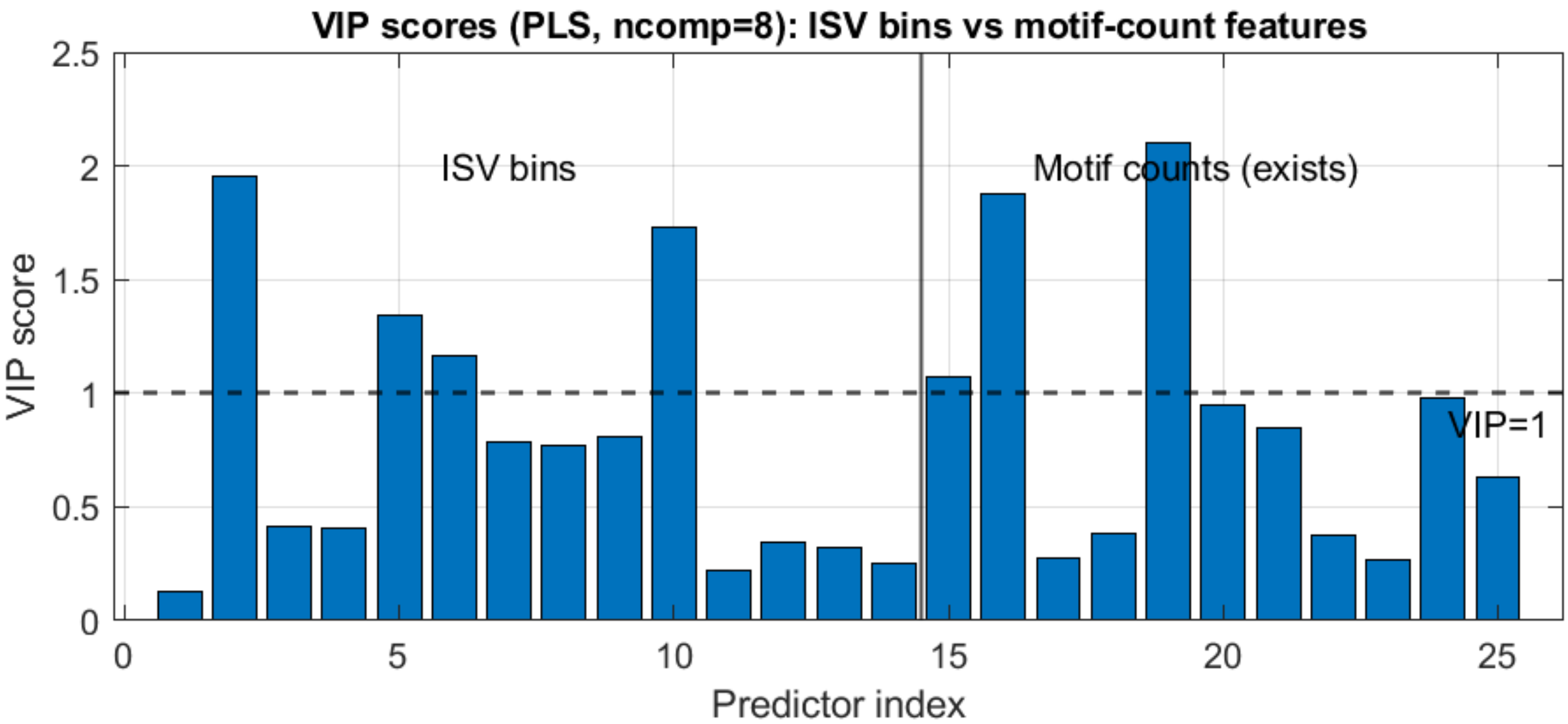

Figure S17. Variable Importance in Projection (VIP) scores for Model 3 using binned ^1H NMR descriptors and heteroatom motif corrections.

S11 - Regression equation of the final PLS model:

To provide an explicit analytical form of the final PLS model, the regression coefficients were back-transformed from the standardized descriptor space to the original variable space. This allows the fitted model to be expressed as a direct linear equation in terms of the experimental descriptors, analogous in form to a conventional multiple linear regression: $\Delta H_f^{\circ}(l) = b_0 + \sum_j b_j x_j$

where x_j are the original ISV bin and motif-count descriptors, b_j are the corresponding back-transformed coefficients, and b_0 is the intercept. The resulting coefficients are listed in Table S12, note that the units are kj mol[-1].

Intercept	-13.0391	ISV 6.75-7.50ppm	10.95254	Exists 06	-160.588
ISV 0.00-0.75ppm	-14.9721	ISV 7.50-8.25ppm	19.47679	Exists 07	11.8798
ISV 0.75-1.50ppm	-13.2872	ISV 8.25-9.00ppm	120.6419	Exists 08	19.46121
ISV 1.50-2.25ppm	-9.8262	ISV 9.00-9.75ppm	-85.1125	Exists 09	42.66984
ISV 2.25-3.00ppm	-6.85906	ISV 9.75-10.50ppm	-53.7371	Exists 10	87.37121
ISV 3.00-3.75ppm	-5.01695	Exists 01	-208.205	Exists 11	-80.5897
ISV 3.75-4.50ppm	-5.20284	Exists 02	-442.876		
ISV 4.50-5.25ppm	13.88171	Exists 03	-94.6011		
ISV 5.25-6.00ppm	21.21965	Exists 04	-182.469		
ISV 6.00-6.75ppm	14.00768	Exists 05	-376.237		

Table S12. Back transformed regression coefficients for the final PLS model expressed in the original descriptor space.

ISV descriptors correspond to integrated proton signal intensities within 0.75 ppm bins spanning the 0 –10.5 ppm chemical shift range. Motif descriptors (exists_01–exists_11) represent count indicators for predefined heteroatom-containing functional group environments used in the model. Each exists descriptor corresponds to a specific motif defined in Table S13.

Exists 1	Exists 2	Exists 3	Exists 4	Exists 5	Exists 6
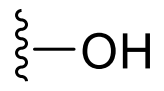	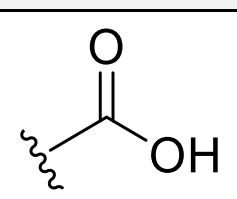	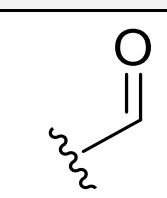	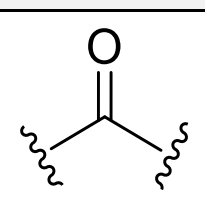	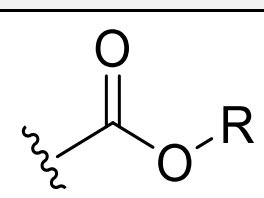	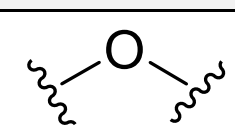
Exists 7	Exists 8	Exists 9	Exists 10	Exists 11	
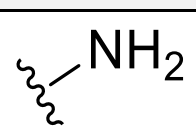	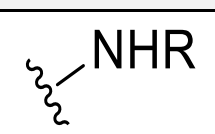	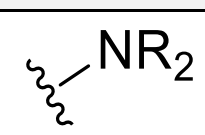	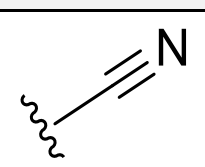	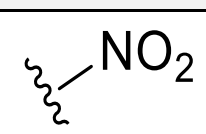	

Table S13. Chemical motif definitions for the heteroatom count descriptors (exists_01–exists_11) used as predictors in the PLS model.

S12 – Reaction enthalpy calculation:

For this comparison, the reactions considered included eight molecules originally present in the 364-molecule dataset. To ensure a legitimate evaluation of truly unseen data, molecules 1–9 were removed from the database prior to model training, resulting in a reduced dataset of 356 molecules used for prediction.

^{1}H NMR spectra for molecules 1-12 were obtained from SDBS[2], whereas the ^{1}H NMR spectrum of molecule 13 was obtained from Enamine LTD[49].

An experimental standard enthalpy of formation of liquid water ($H_2O_{(l)}$) was used in the calculations, with the value of $\Delta H_f^\circ(l)$ = -285.83 (Kj mol^{-1})[66]. Reaction enthalpy sources are listed in the main manuscript. Table S14 summarizes the predictive performance (30 repetitions of nested 10-fold cross-validation) of the model trained on the 356-molecule dataset used for the unseen data predictions. Table S15 summarizes the calculated standard enthalpies of formation for the molecules used in this study. The complete model (Model 3) was used for all calculations.

R^2	0.9887 ± 0.0004
RMSE	23.3 ± 0.5
MAE	14.1 ± 0.3

Table S14. Predictive performance of Model 3 for the 356-molecule dataset based on 30 repeated nested 10-fold cross-validation.

No.	Molecule	CAS	Calculated $\Delta H_f^\circ(l)$ from ^{1}H NMR Using Model III(kj mol^{-1})
1	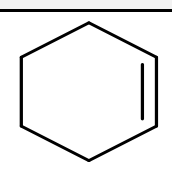	110-83-8	-51.44
2	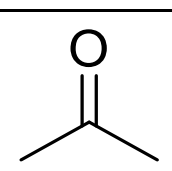	67-64-1	-254.41
3	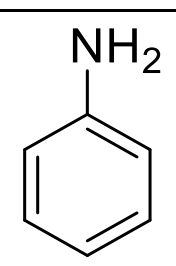	62-53-3	57.03
4	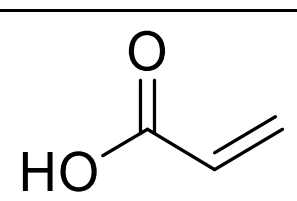	79-10-7	-407.33
5	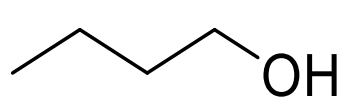	71-36-3	-329.01
6	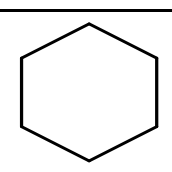	110-82-7	-173.40
7	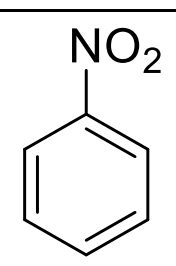	98-95-3	-5.74

8	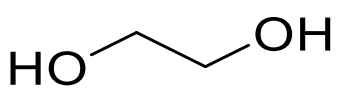	107-21-1	-462.01
9	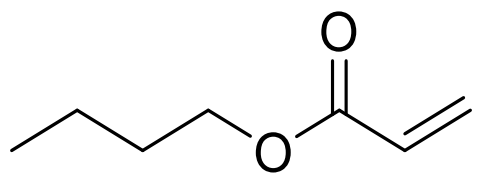	141-32-2	-433.88
10	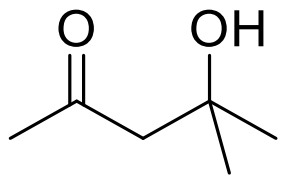	123-42-2	-532.13
11	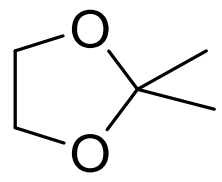	2916-31-6	-433.22
12	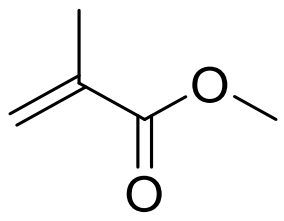	80-62-6	-396.44
13	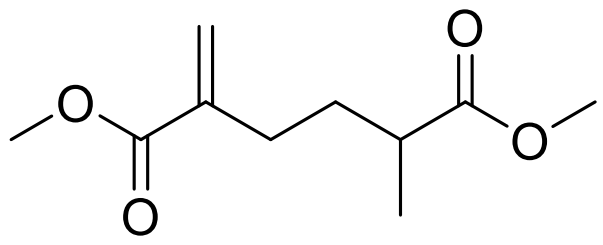	4513-62-6	-840.24

Table S15. Calculated standard enthalpies of formation ($\Delta H_f^\circ(l)$) for molecules used in the unseen-data reaction enthalpy calculations.

S13 - MATLAB Script for Data Analysis:

Note: Each validation section uses a fixed random seed to ensure full reproducibility of the reported metrics.

```
%% ============================================================
%  NMR → ΔHf° approximation via PLS
%  Model definition used in paper:
%    - ISV – Integration Sum Vector: binned proton integrations only (no shift values in X)
%    - Motif counts: coarse functional-group counts (exists matrix)
%  Validation:
%    - Repeated nested 10-fold CV (outer repeats)
%    - Repeated 75/25 holdout with inner CV for ncomp
%    - Y scramble under same nested protocol
%% ============================================================
clear; clc; close all;
warning('off','MATLAB:table:ModifiedAndSavedVarnames');
%% ----------------------- USER SETTINGS -----------------------
rng(2025);
% Binning parameters (paper should match these)
binWidth = 0.75;
ppmMin   = 0.0;
ppmMax   = 10.50;
% Validation parameters
outerK       = 10;
outerRepeats = 30;
holdFrac     = 0.25;
holdRepeats  = 30;
innerK       = 5;     % inner CV folds
maxCompCap   = 40;    % cap on candidate ncomp
% Y-scramble
nScramble    = 500;
% Outputs
outDir = "results_pls_isv";
if ~exist(outDir, 'dir'); mkdir(outDir); end
logFile = fullfile(outDir, "data_parse_log.txt");
summaryCSV = fullfile(outDir, "performance_summary.csv");
%% ------------------------ READ DATA ------------------------
fprintf("Reading input files...\n");
T = readtable("dataset.xlsx");
Y = T.Enthalpy;
shiftStr = T.ShiftVecs;
intStr   = T.IntVecs;
exists = table2array(T(:,5:end));
nMol = height(T);
shiftVecs = cell(nMol,1);
intVecs   = cell(nMol,1);
for i = 1:nMol
    shiftVecs{i} = localParseNums(shiftStr{i});
    intVecs{i}   = localParseNums(intStr{i});
end
%% ----------------------- BINNING → ISV ---------------------
fprintf("Binning to ISV...\n");
edges = ppmMin:binWidth:ppmMax;
nBins = numel(edges) - 1;
ISV = zeros(nMol, nBins);
for r = 1:nMol
    s = shiftVecs{r};
    v = intVecs{r};
    if isempty(s); continue; end
    keep = (s >= ppmMin) & (s < ppmMax) & ~isnan(s) & ~isnan(v);
    s = s(keep);
    v = v(keep);
    if isempty(s); continue; end
    b = discretize(s, edges);
```

```
    ISV(r,:) = accumarray(b(:), v(:), [nBins 1], @sum, 0).';
end
% predictor matrix
X_raw = [ISV,exists];
X_raw(isnan(X_raw)) = 0;
%% ------------------------- METRICS ----------------------------
rmse = @(y,yh) sqrt(mean((y - yh).^2));
mae  = @(y,yh) mean(abs(y - yh));
r2   = @(y,yh) 1 - sum((y - yh).^2) / sum((y - mean(y)).^2);
%% ============================================================
%  A) Repeated nested 10-fold CV
%% ============================================================
fprintf("\nRunning repeated nested %d-fold CV (%d repeats)...\n", outerK, outerRepeats);
cv_R2   = zeros(outerRepeats,1);
cv_RMSE = zeros(outerRepeats,1);
cv_MAE  = zeros(outerRepeats,1);
chosenN_all = [];
rng(1);
for r = 1:outerRepeats
    cv_outer = cvpartition(nMol, 'KFold', outerK);
    yhat_all = nan(nMol,1);
    for f = 1:cv_outer.NumTestSets
        tr = training(cv_outer,f);
        te = test(cv_outer,f);
        [Xtrz, mu, sig] = zscore(X_raw(tr,:));
        sig(sig==0) = 1;
        Xtez = (X_raw(te,:) - mu) ./ sig;
        ytr = Y(tr);
        maxCompLoc = min([maxCompCap, size(Xtrz,2)-1, sum(tr)-1]);
        bestN = local_choose_ncomp_1se(Xtrz, ytr, maxCompLoc, innerK);
        chosenN_all(end+1,1) = bestN;
        [~,~,~,~,beta] = plsregress(Xtrz, ytr, bestN);
        yhat_all(te) = [ones(sum(te),1) Xtez] * beta;
    end
    cv_R2(r)   = r2(Y, yhat_all);
    cv_RMSE(r) = rmse(Y, yhat_all);
    cv_MAE(r)  = mae(Y, yhat_all);
end
fprintf("=== Repeated nested %d-fold CV (n=%d) ===\n", outerK, outerRepeats);
fprintf("R²   = %.4f ± %.4f\n", mean(cv_R2),   std(cv_R2));
fprintf("RMSE = %.2f ± %.2f  (kJ mol^-1)\n", mean(cv_RMSE), std(cv_RMSE));
fprintf("MAE  = %.2f ± %.2f  (kJ mol^-1)\n", mean(cv_MAE),  std(cv_MAE));
fprintf("Chosen ncomp (all folds): mode=%d, median=%d, range=[%d,%d]\n", ...
    mode(chosenN_all), median(chosenN_all), min(chosenN_all), max(chosenN_all));
bestN_global = mode(chosenN_all);
%% ============================================================
%  B) Calibration fit (full data) using bestN_global
%% ============================================================
[Xz, mu_all, sig_all] = zscore(X_raw);
sig_all(sig_all==0) = 1;
[~,~,~,~,betaG] = plsregress(Xz, Y, bestN_global);
yhat_train = [ones(nMol,1) Xz] * betaG;
train_R2   = r2(Y, yhat_train);
train_RMSE = rmse(Y, yhat_train);
train_MAE  = mae(Y, yhat_train);
fprintf("\n=== Calibration (full data) using ncomp=%d ===\n", bestN_global);
fprintf("R²   = %.4f\n", train_R2);
fprintf("RMSE = %.2f  (kJ mol^-1)\n", train_RMSE);
fprintf("MAE  = %.2f  (kJ mol^-1)\n", train_MAE);
%% ============================================================
%  B2) Export linear equation in ORIGINAL (unscaled) predictors
%% ============================================================
beta_z = betaG(2:end);
b_raw  = beta_z(:) ./ sig_all(:);
b0_raw = betaG(1) - sum( (mu_all(:) ./ sig_all(:)) .* beta_z(:) );
nBins = size(ISV,2);
p_total = size(X_raw,2);
p_exists = p_total - nBins;
edges = ppmMin:binWidth:ppmMax;
```

```
binLabels = strings(nBins,1);
for k = 1:nBins
    binLabels(k) = sprintf("ISV_%0.2f-%0.2fppm", edges(k), edges(k+1));
end
existsLabels = strings(p_exists,1);
for j = 1:p_exists
    existsLabels(j) = sprintf("exists_%02d", j);
end
predNames = [binLabels; existsLabels];
% equation coefficients in CSV
EqTbl = table(predNames, b_raw, 'VariableNames', {'Predictor','Coeff_rawX'});
EqTbl = [table("Intercept", b0_raw, 'VariableNames', {'Predictor','Coeff_rawX'}); EqTbl];
writetable(EqTbl, fullfile(outDir, "pls_equation_rawX_coeffs.csv"));
fprintf("\nSaved raw-X linear equation coefficients to pls_equation_rawX_coeffs.csv\n");
fprintf("Equation form: yhat = %.6g + sum_j Coeff_rawX(j) * X_raw(j)\n", b0_raw);
%% ==============================================================
%  C) Repeated 75/25 holdout with inner CV for ncomp
%% ==============================================================
fprintf("\nRunning repeated 75/25 holdout (%d repeats)...\n", holdRepeats);
te_R2   = zeros(holdRepeats,1);
te_RMSE = zeros(holdRepeats,1);
te_MAE  = zeros(holdRepeats,1);
chosenN_HO = zeros(holdRepeats,1);
for r = 1:holdRepeats
    rng(3000 + r);
    ho = cvpartition(nMol, 'HoldOut', holdFrac);
    tr = training(ho); te = test(ho);
    [Xtrz, mu, sig] = zscore(X_raw(tr,:));
    sig(sig==0) = 1;
    Xtez = (X_raw(te,:) - mu) ./ sig;
    ytr = Y(tr); yte = Y(te);
    maxCompLoc = min([maxCompCap, size(Xtrz,2)-1, sum(tr)-1]);
    bestN = local_choose_ncomp_1se(Xtrz, ytr, maxCompLoc, innerK);
    chosenN_HO(r) = bestN;
    [~,~,~,~,beta] = plsregress(Xtrz, ytr, bestN);
    yhat_te = [ones(sum(te),1) Xtez] * beta;
    te_R2(r)   = r2(yte, yhat_te);
    te_RMSE(r) = rmse(yte, yhat_te);
    te_MAE(r)  = mae(yte, yhat_te);
end
fprintf("=== Repeated 75/25 holdout (n=%d) ===\n", holdRepeats);
fprintf("TEST R²   = %.4f ± %.4f\n", mean(te_R2),   std(te_R2));
fprintf("TEST RMSE = %.2f ± %.2f  (kJ mol^-1)\n", mean(te_RMSE), std(te_RMSE));
fprintf("TEST MAE  = %.2f ± %.2f  (kJ mol^-1)\n", mean(te_MAE),  std(te_MAE));
fprintf("Chosen ncomp (holdout): mode=%d, median=%d, range=[%d,%d]\n", ...
    mode(chosenN_HO), median(chosenN_HO), min(chosenN_HO), max(chosenN_HO));
%% ==============================================================
%  D) Parity plot + residual diagnostics (representative nested CV split)
%% ==============================================================
cv_outer = cvpartition(nMol, 'KFold', outerK);
yhat_cv_plot = nan(nMol,1);
for f = 1:cv_outer.NumTestSets
    tr = training(cv_outer,f);
    te = test(cv_outer,f);
    [Xtrz, mu, sig] = zscore(X_raw(tr,:));
    sig(sig==0) = 1;
    Xtez = (X_raw(te,:) - mu) ./ sig;
    ytr = Y(tr);
    maxCompLoc = min([maxCompCap, size(Xtrz,2)-1, sum(tr)-1]);
    bestN = local_choose_ncomp_1se(Xtrz, ytr, maxCompLoc, innerK);
    [~,~,~,~,beta] = plsregress(Xtrz, ytr, bestN);
    yhat_cv_plot(te) = [ones(sum(te),1) Xtez] * beta;
end
% Parity plot
figure('Units','centimeters','Position',[2 2 10 8.5],'Color','w'); hold on;
scatter(Y, yhat_train, 34, 'filled', 'MarkerFaceAlpha', 0.55);
scatter(Y, yhat_cv_plot, 34, 'o', 'MarkerEdgeAlpha', 0.85);
xy = [min(Y) max(Y)];
plot(xy, xy, 'k--', 'LineWidth', 1.0);
```

```
axis equal; grid on; box on;
xlabel('Experimental \DeltaH_f^\circ (kJ mol^{-1})');
ylabel('Predicted \DeltaH_f^\circ (kJ mol^{-1})');
title(sprintf('PLS (ISV + motif counts): Calibration (ncomp=%d) + nested CV example', bestN_global),
...
      'FontName','Arial','FontSize',9.5);
legend({'Calibration','Nested CV','y=x'}, 'Location','southeast', 'Box','on', ...
       'FontName','Arial','FontSize',9.0);
print(gcf, fullfile(outDir,"parity_calibration_vs_nestedCV.png"), '-dpng', '-r600');
% Residual diagnostics
res_train = Y - yhat_train;
res_cv    = Y - yhat_cv_plot;
figure('Color','w');
subplot(1,2,1);
scatter(yhat_train, res_train, 20, 'filled'); grid on; box on; yline(0,'k--');
xlabel('Predicted \DeltaH_f^\circ'); ylabel('Residual (kJ mol^{-1})');
title('Calibration residuals');
subplot(1,2,2);
scatter(yhat_cv_plot, res_cv, 20, 'filled'); grid on; box on; yline(0,'k--');
xlabel('Predicted \DeltaH_f^\circ'); ylabel('Residual (kJ mol^{-1})');
title('Nested CV residuals (representative split)');
print(gcf, fullfile(outDir,"residuals_cal_vs_cv.png"), '-dpng', '-r600');
figure('Color','w');
histogram(res_cv, 50); grid on; box on;
xlabel('Residual (kJ mol^{-1})'); ylabel('Count');
title('CV residual distribution (representative split)');
print(gcf, fullfile(outDir,"residual_hist_cv.png"), '-dpng', '-r600');
figure('Color','w');
scatter(Y, res_cv, 20, 'filled'); grid on; box on; yline(0,'k--');
xlabel('Experimental \DeltaH_f^\circ (kJ mol^{-1})');
ylabel('Residual (kJ mol^{-1})');
title('Residual vs experimental (nested CV, representative split)');
print(gcf, fullfile(outDir,"residual_vs_experimental.png"), '-dpng', '-r600');
%% =========================================================
%  E) Y-scramble test (nested CV)
%% =========================================================
fprintf("\nRunning Y-scramble (nested CV), n=%d...\n", nScramble);
true_R2 = mean(cv_R2);
scr_R2   = zeros(nScramble,1);
scr_RMSE = zeros(nScramble,1);
scr_MAE  = zeros(nScramble,1);
rng(777);
for s = 1:nScramble
    Yperm = Y(randperm(nMol));
    cv_outer = cvpartition(nMol, 'KFold', outerK);
    yhat_all = nan(nMol,1);
    for f = 1:cv_outer.NumTestSets
        tr = training(cv_outer,f);
        te = test(cv_outer,f);
        [Xtrz, mu, sig] = zscore(X_raw(tr,:));
        sig(sig==0) = 1;
        Xtez = (X_raw(te,:) - mu) ./ sig;
        ytr = Yperm(tr);
        maxCompLoc = min([maxCompCap, size(Xtrz,2)-1, sum(tr)-1]);
        bestN = local_choose_ncomp_1se(Xtrz, ytr, maxCompLoc, innerK);
        [~,~,~,~,beta] = plsregress(Xtrz, ytr, bestN);
        yhat_all(te) = [ones(sum(te),1) Xtez] * beta;
    end
    scr_R2(s)   = r2(Yperm, yhat_all);
    scr_RMSE(s) = rmse(Yperm, yhat_all);
    scr_MAE(s)  = mae(Yperm, yhat_all);
end
p_emp = (1 + sum(scr_R2 >= true_R2)) / (nScramble + 1);
fprintf("=== Y-scramble (nested CV) ===\n");
fprintf("True nested-CV mean R² = %.4f\n", true_R2);
fprintf("Scrambled R²: mean=%.3f ± %.3f (min=%.3f, max=%.3f)\n", ...
    mean(scr_R2), std(scr_R2), min(scr_R2), max(scr_R2));
fprintf("Empirical p-value vs scramble: p = %.4g\n", p_emp);
figure('Color','w');
```

```
histogram(scr_R2, 25); grid on; box on;
xline(true_R2, 'r', 'LineWidth', 1.8);
xlabel('Nested-CV R^2 under Y-scramble');
ylabel('Count');
title(sprintf('Y-scramble (n=%d). Red=true nested-CV R^2', nScramble));
print(gcf, fullfile(outDir,"y_scramble_hist.png"), '-dpng', '-r600');
%% ============================================================
%  F) Variable Importance in Projection (VIP)
%% ============================================================
fprintf("\nComputing VIP scores (calibration model, ncomp=%d)...\n", bestN_global);
[~,~,~,~,~,PCTVAR,~,statsVIP] = plsregress(Xz, Y, bestN_global);
vip = local_vip_pls(statsVIP.W, PCTVAR(2,1:bestN_global));
% blocks: [ISV, exists]
nBins   = size(ISV,2);
p_total = size(X_raw,2);
p_exists = p_total - nBins;
vip_isv    = vip(1:nBins);
vip_exists = vip(nBins+1:end);
meanVIP_isv    = mean(vip_isv);
meanVIP_exists = mean(vip_exists);
share_isv    = sum(vip_isv.^2) / sum(vip.^2);
share_exists = sum(vip_exists.^2) / sum(vip.^2);
fprintf("VIP block summary:\n");
fprintf("  ISV   : mean VIP = %.3f | share (sum VIP^2) = %.1f%%\n", meanVIP_isv,    100*share_isv);
fprintf("  exists: mean VIP = %.3f | share (sum VIP^2) = %.1f%%\n", meanVIP_exists, 100*share_exists);
fprintf("VIP>1 counts:\n");
fprintf("  ISV   : %d / %d\n", nnz(vip_isv > 1), numel(vip_isv));
fprintf("  exists: %d / %d\n", nnz(vip_exists > 1), numel(vip_exists));
p_total = size(X_raw,2);
nBins   = size(ISV,2);
p_exists = p_total - nBins;
if numel(vip) ~= p_total
    error("VIP length (%d) does not match number of predictors (%d).", ...
          numel(vip), p_total);
end
edges = ppmMin:binWidth:ppmMax;
binLabels = strings(nBins,1);
for k = 1:nBins
    binLabels(k) = sprintf("ISV_%0.2f-%0.2fppm", edges(k), edges(k+1));
end
existsLabels = strings(p_exists,1);
for j = 1:p_exists
    existsLabels(j) = sprintf("exists_%02d", j);
end
varNames = [binLabels; existsLabels];
if numel(varNames) ~= numel(vip)
    error("Label length mismatch after construction.");
end
VIPtbl = table(cellstr(varNames), vip(:), ...
    'VariableNames', {'Predictor','VIP'});
writetable(VIPtbl, fullfile(outDir,'vip_scores.csv'));
figure('Color','w','Units','centimeters','Position',[2 2 18 7]);
bar(vip); hold on; grid on; box on;
yline(1,'k--','LineWidth',1.2,'Label','VIP=1','LabelVerticalAlignment','bottom');
xline(nBins+0.5,'k-','LineWidth',1.0);
xlabel('Predictor index');
ylabel('VIP score');
title(sprintf('VIP scores (PLS, ncomp=%d): ISV bins vs motif-count features', bestN_global), ...
      'FontName','Arial','FontSize',10);
text(nBins/2, max(vip)*0.95, "ISV bins", 'HorizontalAlignment','center', 'FontName','Arial');
text(nBins + p_exists/2, max(vip)*0.95, "Motif counts (exists)", 'HorizontalAlignment','center',
'FontName','Arial');
print(gcf, fullfile(outDir,"vip_plot.png"), '-dpng','-r600');
fprintf("Saved VIP plot + CSV to %s\n", outDir);
%% -------------------- SAVE SUMMARY TABLE ---------------------
Tsaveddata = table;
Tsaveddata.n = nMol;
Tsaveddata.binWidth = binWidth;
Tsaveddata.ppmMin = ppmMin;
```

```
Tsaveddata.ppmMax = ppmMax;
Tsaveddata.train_R2   = train_R2;
Tsaveddata.train_RMSE = train_RMSE;
Tsaveddata.train_MAE  = train_MAE;
Tsaveddata.cv_R2_mean   = mean(cv_R2);
Tsaveddata.cv_R2_sd     = std(cv_R2);
Tsaveddata.cv_RMSE_mean = mean(cv_RMSE);
Tsaveddata.cv_RMSE_sd   = std(cv_RMSE);
Tsaveddata.cv_MAE_mean  = mean(cv_MAE);
Tsaveddata.cv_MAE_sd    = std(cv_MAE);
Tsaveddata.ho_R2_mean   = mean(te_R2);
Tsaveddata.ho_R2_sd     = std(te_R2);
Tsaveddata.ho_RMSE_mean = mean(te_RMSE);
Tsaveddata.ho_RMSE_sd   = std(te_RMSE);
Tsaveddata.ho_MAE_mean  = mean(te_MAE);
Tsaveddata.ho_MAE_sd    = std(te_MAE);
Tsaveddata.ncomp_mode   = bestN_global;
Tsaveddata.ncomp_range  = string(sprintf("[%d,%d]", min(chosenN_all), max(chosenN_all)));
Tsaveddata.yScramble_n = nScramble;
Tsaveddata.yScramble_p = p_emp;
Tsaveddata.yScramble_R2_mean = mean(scr_R2);
Tsaveddata.yScramble_R2_max  = max(scr_R2);
writetable(Tsaveddata, summaryCSV);
fprintf("\nSaved summary CSV to: %s\n", summaryCSV);
fprintf("Saved figures to: %s\n", outDir);
%% ============================================================
%  Benson comparison (matched subset, fair + consistent)
%  benson.xlsx expected columns:
%    col1 = Benson-predicted ΔHf°
%    col2 = Experimental ΔHf° (for the same subset)
%  idx_map maps rows in benson.xlsx -> rows in your dataset arrays
%% ============================================================
B = readmatrix('benson.xlsx');
Y_benson = B(:,1);   % Benson predictions
Y_exp_B  = B(:,2);   % experimental values used for parity x-axis (subset)
% Indices of molecules in the main dataset that have Benson group
% estimates available (matched by CAS).
% Generated separately; see SI Table S11.
idx_map = [ ...
231; 184; 208; 284; 159; 1; 240; 91; 98; 103;...
253; 122; 128; 276; 173; 142; 296; 147; 304; 311;...
164; 171; 325; 328; 41; 357; 85; 255; 132; 196;...
353; 133; 337; 74; 264; 314; 346; 4; 23; 25; 13; 22;
]';
n = numel(Y);
if any(idx_map < 1) || any(idx_map > n)
    error('idx_map contains indices outside the dataset range 1..%d. Check row filtering / mapping.', 
n);
end
if numel(idx_map) ~= numel(Y_exp_B) || numel(idx_map) ~= numel(Y_benson)
    error('Length mismatch: idx_map (%d) vs benson rows (%d).', numel(idx_map), size(B,1));
end
delta = Y(idx_map) - Y_exp_B;
if max(abs(delta)) > 1e-6
    warning('Experimental values in benson.xlsx do not exactly match Y(idx_map). Max abs diff = %.3g. 
Using Y(idx_map) as ground truth.', max(abs(delta)));
end
Y_true = Y(idx_map);
Y_model_CV = yhat_cv_plot(idx_map);
Y_model_CAL = yhat_train(idx_map);
% ---------- metrics helper ----------
metrics = @(ytrue,yhat) struct( ...
    'R2',   1 - sum((ytrue - yhat).^2)/sum((ytrue - mean(ytrue)).^2), ...
    'RMSE', sqrt(mean((ytrue - yhat).^2)), ...
    'MAE',  mean(abs(ytrue - yhat)) );
M_ben = metrics(Y_exp_B, Y_benson);
M_cv  = metrics(Y_exp_B, Y_model_CV);
M_cal = metrics(Y_exp_B, Y_model_CAL);
fprintf('\n=== Matched subset comparison (n=%d) ===\n', numel(Y_true));
```

```
fprintf('Benson          : R2=%.4f  RMSE=%.2f  MAE=%.2f\n', M_ben.R2, M_ben.RMSE, M_ben.MAE);
fprintf('Model (nested CV): R2=%.4f  RMSE=%.2f  MAE=%.2f\n', M_cv.R2,  M_cv.RMSE,  M_cv.MAE);
fprintf('Model (calib)    : R2=%.4f  RMSE=%.2f  MAE=%.2f  [reference only]\n', ...
        M_cal.R2, M_cal.RMSE, M_cal.MAE);
% ---------- (Benson vs Model CV) ----------
figure('Color','w','Units','centimeters','Position',[2 2 12.5 6]);
hold on; grid on; box on;
plot(Y_exp_B, Y_benson, 'o', 'LineWidth',1.2, 'MarkerSize',5, ...
    'DisplayName', sprintf('Benson  (R2=%.4f)', M_ben.R2));
plot(Y_exp_B, Y_model_CV, 's', 'LineWidth',1.2, 'MarkerSize',5, ...
    'DisplayName', sprintf('Model (nested CV)  (R2=%.4f)', M_cv.R2));
mn = min([Y_exp_B; Y_benson; Y_model_CV]);
mx = max([Y_exp_B; Y_benson; Y_model_CV]);
plot([mn mx],[mn mx],'k--','DisplayName','y = x');
xlabel('Experimental \it{\DeltaH_f^\circ} (kJ mol^{-1})');
ylabel('Predicted \it{\DeltaH_f^\circ} (kJ mol^{-1})');
title('Matched-subset benchmark: Benson vs NMR-PLS (nested CV)','FontName','Arial','FontSize',9.5);
legend('Location','southeast','Box','on','FontName','Arial','FontSize',9.0);
xlim([mn mx]); ylim([mn mx]);
print(gcf, fullfile(outDir,'benson_vs_model_nestedCV.png'), '-dpng','-r600');
%% ====================== LOCAL FUNCTIONS =====================
function v = localParseNums(x)
    % Parse a cell/entry into numeric ROW vector (possibly empty)
    if iscell(x) && numel(x)==1
        x = x{1};
    end
    if isempty(x)
        v = [];
        return
    end
    if isnumeric(x)
        v = x(:).';
        v = v(~isnan(v));
        return
    end
    try
        s = string(x);
    catch
        v = [];
        return
    end
    if numel(s) > 1
        s = join(s, " ");
    end
    s = strtrim(s);
    if strlength(s)==0 || any(ismissing(s))
        v = [];
        return
    end
    s = regexprep(s, "[,;]", " ");
    tokens = regexp(s, "[-+]?\d*\.?\d+(?:[eE][-+]?\d+)?", "match");
    if isempty(tokens)
        v = [];
    else
        v = str2double(tokens);
        v = v(~isnan(v));
        v = v(:).';
    end
end
function bestN = local_choose_ncomp_1se(Xtrz, ytr, maxComp, innerK)
    cv_in = cvpartition(numel(ytr), 'KFold', innerK);
    rmse_by_comp = zeros(maxComp,1);
    se_by_comp   = zeros(maxComp,1);
    for nc = 1:maxComp
        fold_rmse = zeros(cv_in.NumTestSets,1);
        for f = 1:cv_in.NumTestSets
            tr2 = training(cv_in,f);
            te2 = test(cv_in,f);
            [~,~,~,~,beta] = plsregress(Xtrz(tr2,:), ytr(tr2), nc);
```

```
            yhat = [ones(sum(te2),1) Xtrz(te2,:)] * beta;
            fold_rmse(f) = sqrt(mean((ytr(te2) - yhat).^2));
        end
        rmse_by_comp(nc) = mean(fold_rmse);
        se_by_comp(nc)   = std(fold_rmse) / sqrt(cv_in.NumTestSets);
    end
    [min_rmse, idx_min] = min(rmse_by_comp);
    thr = min_rmse + se_by_comp(idx_min); % 1-SE threshold
    cand = find(rmse_by_comp <= thr);
    bestN = cand(1); % smallest within 1-SE
end
%% -------- local VIP function (place near other local functions) --------
function vip = local_vip_pls(W, PCTVAR_Y)
    % Here we normalize SSY to sum=1, so denominator is 1.
    [p, A] = size(W);
    SSY = PCTVAR_Y(:)';             % row
    SSY = SSY / sum(SSY);           % normalize to sum=1
    w2 = sum(W.^2,1);               % 1 x A, ||w_a||^2
    vip = zeros(p,1);
    for j = 1:p
        vip(j) = sqrt( p * sum( SSY .* (W(j,:).^2 ./ w2) ) );
    end
end
%%
```

S14 - References:

(1) Rumble, J. R. *CRC Handbook of Chemistry and Physics Online*, 106th ed.; CRC Press: Boca Raton, FL, 2025.

(2) *AIST:Spectral Database for Organic Compounds,SDBS*. https://sdbs.db.aist.go.jp/SearchInformation.aspx (accessed 2026-02-21).

(3) Yao, Y.-Y.; Zhao, X.-L. A Rapid, Efficient Method for Deprotection of Oximes to Carbonyl Compounds with NaClO2 in Water. *Lett. Org. Chem.* *12* (1), 73–76. https://doi.org/10.2174/157017861201150112125024.

(4) Chemical Abstracts Service (CAS). *CAS SciFinder^n Spectral Data (Spectral Data Library, John Wiley & Sons, Inc.)*. CAS SciFinder^n. https://scifinder.cas.org/.

(5) *Regioselective Arene Functionalization: Simple Substitution of Carboxylate by Alkyl Groups - Krüger - 2009 - Chemistry – A European Journal - Wiley Online Library*. https://chemistry-europe.onlinelibrary.wiley.com/doi/10.1002/chem.200901774 (accessed 2026-02-22).

(6) Cherney, A. H.; Hedley, S. J.; Mennen, S. M.; Tedrow, J. S. Xantphos as a Branch-Selective Ligand for the Acyclic Sec-Alkyl Negishi Cross-Coupling of Heteroaryl Halides. *Organometallics* **2019**, *38* (1), 97–102. https://doi.org/10.1021/acs.organomet.8b00590.

(7) Rickert, A.; Krombach, V.; Hamers, O.; Zorn, H.; Maison, W. Enzymatic Allylic Oxidations with a Lyophilisate of the Edible Fungus Pleurotus Sapidus. *Green Chem.* **2012**, *14* (3), 639–644. https://doi.org/10.1039/C2GC16317A.

(8) Hydrolysis of Diazonium Salts Using a Two-Phase System (CPME and Water). https://doi.org/10.1002/hc.21275.

(9) *Nickel-Catalyzed Negishi Cross-Coupling Reactions of Secondary Alkylzinc Halides and Aryl Iodides | Organic Letters*. https://pubs.acs.org/doi/10.1021/ol200098d (accessed 2026-02-22).

(10) Zhu, R.; Jiang, J.-L.; Li, X.-L.; Deng, J.; Fu, Y. A Comprehensive Study on Metal Triflate-Promoted Hydrogenolysis of Lactones to Carboxylic Acids: From Synthetic and Mechanistic Perspectives. *ACS Catal.* **2017**, *7* (11), 7520–7528. https://doi.org/10.1021/acscatal.7b01569.

(11) Dai, P.-F.; Qu, J.-P.; Kang, Y.-B. Organocatalyzed Aerobic Oxidation of Aldehydes to Acids. *Org. Lett.* **2019**, *21* (5), 1393–1396. https://doi.org/10.1021/acs.orglett.9b00101.

(12) *Manganese(I)-Catalyzed β-Methylation of Alcohols Using Methanol as C1 Source - Kaithal - 2020 - Angewandte Chemie International Edition - Wiley Online Library*. https://onlinelibrary.wiley.com/doi/10.1002/anie.201909035 (accessed 2026-02-22).

(13) *A Well-Defined Monomeric Aluminum Complex as an Efficient and General Catalyst in the Meerwein–Ponndorf–Verley Reduction - McNerney - 2014 - Chemistry – A European Journal - Wiley Online Library*. https://chemistry-europe.onlinelibrary.wiley.com/doi/10.1002/chem.201404994 (accessed 2026-02-22).

(14) *Electrophilic Cleavage of Cyclopropylmethystannanes: An Experimental Comparison of σ−σ and σ−π Conjugation | The Journal of Organic Chemistry*. https://pubs.acs.org/doi/10.1021/jo047822p (accessed 2026-02-22).

(15) Ray, R.; Jana, R. D.; Bhadra, M.; Maiti, D.; Lahiri, G. K. Efficient and Simple Approaches Towards Direct Oxidative Esterification of Alcohols. *Chem. – Eur. J.* **2014**, *20* (47), 15618–15624. https://doi.org/10.1002/chem.201403786.

(16) Minakawa, M.; Baek, H.; Yamada, Y. M. A.; Han, J. W.; Uozumi, Y. Direct Dehydrative Esterification of Alcohols and Carboxylic Acids with a Macroporous Polymeric Acid Catalyst. *Org. Lett.* **2013**, *15* (22), 5798–5801. https://doi.org/10.1021/ol4028495.

(17) Rogow, D. L.; Fei, H.; Brennan, D. P.; Ikehata, M.; Zavalij, P. Y.; Oliver, A. G.; Oliver, S. R. J. Hydrothermal Synthesis of Two Cationic Bismuthate Clusters: An Alkylenedisulfonate

Bridged Hexamer, [Bi6O4(OH)4(H2O)2][(CH2)2(SO3)2]3 and a Rare Nonamer Templated by Triflate, [Bi9O8(OH)6][CF3SO3]5. *Inorg. Chem.* **2010**, *49* (12), 5619–5624. https://doi.org/10.1021/ic1004402.
(18) Murray, A. T.; Matton, P.; Fairhurst, N. W. G.; John, M. P.; Carbery, D. R. Biomimetic Flavin-Catalyzed Aldehyde Oxidation. *Org. Lett.* **2012**, *14* (14), 3656–3659. https://doi.org/10.1021/ol301496m.
(19) Chemical Abstracts Service (CAS). *CAS SciFinder^n Spectral Data (Advanced Chemistry Development, Inc.)*. CAS SciFinder^n. https://scifinder.cas.org/.
(20) Tsai, B.-C.; Liu, Y.-H.; Peng, S.-M.; Liu, S.-T. Coordination Chemistry of an Unsymmetrical Naphthyridine-Based Tetradentate Ligand toward Various Transition-Metal Ions. *Eur. J. Inorg. Chem.* **2016**, *2016* (17), 2783–2790. https://doi.org/10.1002/ejic.201600163.
(21) *A New Efficient Catalytic System for the Chemoselective Cobalt-Catalyzed Cross-Coupling of Aryl Grignard Reagents with Primary and Secondary Alkyl Bromides | Organic Letters*. https://pubs.acs.org/doi/10.1021/ol802362e (accessed 2026-02-22).
(22) Perdriau, S.; Chang, M.-C.; Otten, E.; Heeres, H. J.; de Vries, J. G. Alkene Isomerisation Catalysed by a Ruthenium PNN Pincer Complex. *Chem. – Eur. J.* **2014**, *20* (47), 15434–15442. https://doi.org/10.1002/chem.201403236.
(23) Jones, I. C.; Sharman, G. J.; Pidgeon, J. 1H and 13C NMR Data to Aid the Identification and Quantification of Residual Solvents by NMR Spectroscopy. *Magn. Reson. Chem.* **2005**, *43* (6), 497–509. https://doi.org/10.1002/mrc.1578.
(24) Osako, T.; Torii, K.; Uozumi, Y. Aerobic Flow Oxidation of Alcohols in Water Catalyzed by Platinum Nanoparticles Dispersed in an Amphiphilic Polymer. *RSC Adv.* **2014**, *5* (4), 2647–2654. https://doi.org/10.1039/C4RA14947E.
(25) Mishra, A. K.; Moorthy, J. N. Mechanochemical Catalytic Oxidations in the Solid State with in Situ-Generated Modified IBX from 3,5-Di-Tert-Butyl-2-Iodobenzoic Acid (DTB-IA)/Oxone. *Org. Chem. Front.* **2017**, *4* (3), 343–349. https://doi.org/10.1039/C6QO00588H.
(26) Chan, A. P. Y.; Parkinson, J. A.; Rosair, G. M.; Welch, A. J. Bis(Phosphine)Hydridorhodacarborane Derivatives of 1,1'-Bis(Ortho-Carborane) and Their Catalysis of Alkene Isomerization and the Hydrosilylation of Acetophenone. *Inorg. Chem.* **2020**, *59* (3), 2011–2023. https://doi.org/10.1021/acs.inorgchem.9b03351.
(27) Geng, H.; Huang, P.-Q. Versatile and Chemoselective Transformation of Aliphatic and Aromatic Secondary Amides to Nitriles. *Tetrahedron* **2015**, *71* (23), 3795–3801. https://doi.org/10.1016/j.tet.2015.03.094.
(28) *Direct Preparation of Nitriles from Carboxylic Acids in Continuous Flow | The Journal of Organic Chemistry*. https://pubs.acs.org/doi/10.1021/jo401945r (accessed 2026-02-22).
(29) Maegawa, T.; Akashi, A.; Yaguchi, K.; Iwasaki, Y.; Shigetsura, M.; Monguchi, Y.; Sajiki, H. Efficient and Practical Arene Hydrogenation by Heterogeneous Catalysts under Mild Conditions. *Chem. – Eur. J.* **2009**, *15* (28), 6953–6963. https://doi.org/10.1002/chem.200900361.
(30) *Metal‐Dependent Reaction Tuning with Cyclopentylmetal Reagents: Application to the Asymmetric Synthesis of (+)‐α‐Conhydrine and (S)‐2‐Cyclopentyl‐2‐phenylglycolic Acid - Roy - 2009 - Chemistry – A European Journal - Wiley Online Library*. https://chemistry-europe.onlinelibrary.wiley.com/doi/10.1002/chem.200801603 (accessed 2026-02-22).
(31) Wiitala, K. W.; Al-Rashid, Z. F.; Dvornikovs, V.; Hoye, T. R.; Cramer, C. J. Evaluation of Various DFT Protocols for Computing 1H and 13C Chemical Shifts to Distinguish Stereoisomers: Diastereomeric 2-, 3-, and 4-Methylcyclohexanols as a Test Set. *J. Phys. Org. Chem.* **2007**, *20* (5), 345–354. https://doi.org/10.1002/poc.1151.
(32) Witt, T.; Häußler, M.; Kulpa, S.; Mecking, S. Chain Multiplication of Fatty Acids to Precise Telechelic Polyethylene. *Angew. Chem. Int. Ed.* **2017**, *56* (26), 7589–7594. https://doi.org/10.1002/anie.201702796.

(33) Chemical Abstracts Service (CAS). *CAS SciFinder^n Spectral Data (Bio-Rad Laboratories)*. CAS SciFinder^n. https://scifinder.cas.org/.
(34) *LARGE SCALE, EFFICIENT SYNTHESIS OF 9-UNSUBSTITUTED DIPYRRINONE: Synthetic Communications: Vol 32, No 7*. https://www.tandfonline.com/doi/abs/10.1081/SCC-120003151 (accessed 2026-02-23).
(35) Chemical Abstracts Service (CAS). *CAS SciFinder^n Spectral Data (SDBS, National Institute of Advanced Industrial Science and Technology, Japan)*. CAS SciFinder^n. https://scifinder.cas.org/.
(36) Terent'ev, A. O.; Borisov, D. A.; Yaremenko, I. A.; Ogibin, Y. N.; Nikishin, G. I. Oxidation of Substituted β-Diketones with Hydrogen Peroxide: Synthesis of Esters through the Formation of Bridged 1,2,4,5-Tetraoxanes. *Synthesis* **2010**, *2010* (07), 1145–1149. https://doi.org/10.1055/s-0029-1219225.
(37) Siddiki, S. M. A. H.; Toyao, T.; Kon, K.; Touchy, A. S.; Shimizu, K. Catalytic Hydrolysis of Hydrophobic Esters on/in Water by High-Silica Large Pore Zeolites. *J. Catal.* **2016**, *344*, 741–748. https://doi.org/10.1016/j.jcat.2016.08.021.
(38) Li, X.-L.; Zhang, K.; Jiang, J.-L.; Zhu, R.; Wu, W.-P.; Deng, J.; Fu, Y. Synthesis of Medium-Chain Carboxylic Acids or α,ω-Dicarboxylic Acids from Cellulose-Derived Platform Chemicals. *Green Chem.* **2018**, *20* (2), 362–368. https://doi.org/10.1039/C7GC03125D.
(39) Mao, F.; Sui, D.; Qi, Z.; Fan, H.; Chen, R.; Huang, J. Heterogeneous Cobalt Catalysts for Reductive Amination with H2: General Synthesis of Secondary and Tertiary Amines. *RSC Adv.* **2016**, *6* (96), 94068–94073. https://doi.org/10.1039/C6RA21415K.
(40) Liang, R.; Li, S.; Wang, R.; Lu, L.; Li, F. N-Methylation of Amines with Methanol Catalyzed by a Cp*Ir Complex Bearing a Functional 2,2'-Bibenzimidazole Ligand. *Org. Lett.* **2017**, *19* (21), 5790–5793. https://doi.org/10.1021/acs.orglett.7b02723.
(41) *N,N-Dipropyl-1-propanamine(102-69-2) 1H NMR spectrum*. https://www.chemicalbook.com/SpectrumEN_102-69-2_1HNMR.htm (accessed 2026-02-23).
(42) Khumraksa, B.; Phakhodee, W.; Pattarawarapan, M. Rapid Oxidation of Organic Halides with *N*-Methylmorpholine N-Oxide in an Ionic Liquid under Microwave Irradiation. *Tetrahedron Lett.* **2013**, *54* (15), 1983–1986. https://doi.org/10.1016/j.tetlet.2013.01.131.
(43) Qiu, J. C.; Pradhan, P. P.; Blanck, N. B.; Bobbitt, J. M.; Bailey, W. F. Selective Oxoammonium Salt Oxidations of Alcohols to Aldehydes and Aldehydes to Carboxylic Acids. *Org. Lett.* **2012**, *14* (15), 4034–4034. https://doi.org/10.1021/ol301884a.
(44) Sawama, Y.; Morita, K.; Asai, S.; Kozawa, M.; Tadokoro, S.; Nakajima, J.; Monguchi, Y.; Sajiki, H. Palladium on Carbon-Catalyzed Aqueous Transformation of Primary Alcohols to Carboxylic Acids Based on Dehydrogenation under Mildly Reduced Pressure. *Adv. Synth. Catal.* **2015**, *357* (6), 1205–1210. https://doi.org/10.1002/adsc.201401123.
(45) Chinta, S. P.; Goller, S.; Uhl, G.; Schulz, S. Identification and Synthesis of Branched Wax-Type Esters, Novel Surface Lipids from the Spider Argyrodes Elevatus (Araneae: Theridiidae). *Chem. Biodivers.* **2016**, *13* (9), 1202–1220. https://doi.org/10.1002/cbdv.201600020.
(46) Shukla, P.; Sharma, A.; Pallavi, B.; Cheng, C. H. Nickel-Catalyzed Reductive Heck Type Coupling of Saturated Alkyl Halides with Acrylates and Oxabenzonorbornadiene. *Tetrahedron* **2015**, *71* (15), 2260–2266. https://doi.org/10.1016/j.tet.2015.02.067.
(47) Amézquita-Valencia, M.; Achonduh, G.; Alper, H. Pd-Catalyzed Regioselective Alkoxycarbonylation of 1-Alkenes Using a Lewis Acid [SnCl2 or Ti(OiPr)4] and a Phosphine. *J. Org. Chem.* **2015**, *80* (12), 6419–6424. https://doi.org/10.1021/acs.joc.5b00851.
(48) Chu, Q.; He, G.; Xi, Y.; Wang, P.; Yu, H.; Liu, R.; Zhu, H. Green Synthesis of Low-Carbon Chain Nitroalkanes via a Novel Tandem Reaction of Ketones Catalyzed by TS-1. *Catal. Commun.* **2018**, *108*, 46–50. https://doi.org/10.1016/j.catcom.2018.01.022.

(49) Chemical Abstracts Service (CAS). *CAS SciFinder^n Spectral Data (Enamine Ltd.)*. CAS SciFinder^n. https://scifinder.cas.org/ (accessed 2026-02-22).
(50) Metz, A. E.; Kozlowski, M. C. 2-Aryl-2-Nitroacetates as Central Precursors to Aryl Nitromethanes, α-Ketoesters, and α-Amino Acids. *J. Org. Chem.* **2013**, *78* (2), 717–722. https://doi.org/10.1021/jo302071s.
(51) Marcé, P.; Lynch, J.; Blacker, A. J.; Williams, J. M. J. Conversion of Nitroalkanes into Carboxylic Acids via Iodide Catalysis in Water. *Chem. Commun.* **2016**, *52* (5), 1013–1016. https://doi.org/10.1039/C5CC08681G.
(52) Vidal-Albalat, A.; Świderek, K.; Izquierdo, J.; Rodríguez, S.; Moliner, V.; González, F. V. Catalytic Enantioselective Epoxidation of Nitroalkenes. *Chem. Commun.* **2016**, *52* (65), 10060–10063. https://doi.org/10.1039/C6CC03539F.
(53) Bailey, W. F.; Gagnier, R. P.; Patricia, J. J. Reactions of Tert-Butyllithium with .Alpha.,.Omega.-Dihaloalkanes. Evidence for Single-Electron-Transfer-Mediated Metal-Halogen Interchange Involving Alkyl Radical-Halide Ion Adducts. *J. Org. Chem.* **1984**, *49* (12), 2098–2107. https://doi.org/10.1021/jo00186a004.
(54) Pan, Y.; Luo, Z.; Xu, X.; Zhao, H.; Han, J.; Xu, L.; Fan, Q.; Xiao, J. Ru-Catalyzed Deoxygenative Transfer Hydrogenation of Amides to Amines with Formic Acid/Triethylamine. *Adv. Synth. Catal.* **2019**, *361* (16), 3800–3806. https://doi.org/10.1002/adsc.201900406.
(55) Ballini, R.; Barboni, L.; Giarlo, G. The First Conversion of Primary Alkyl Halides to Nitroalkanes under Aqueous Medium. *J. Org. Chem.* **2004**, *69* (20), 6907–6908. https://doi.org/10.1021/jo049048b.
(56) Shao, P.; Wang, S.; Chen, C.; Xi, C. Cp2TiCl2-Catalyzed Regioselective Hydrocarboxylation of Alkenes with CO2. *Org. Lett.* **2016**, *18* (9), 2050–2053. https://doi.org/10.1021/acs.orglett.6b00665.
(57) Erdik, E.; Koçoğlu, M. Copper Catalyzed Magnesium-Barbier Reaction for γ-Selective Alkyl–Allyl Coupling. *Tetrahedron Lett.* **2007**, *48* (24), 4211–4214. https://doi.org/10.1016/j.tetlet.2007.04.060.
(58) Nguyen, T. V.; Lyons, D. J. M. A Novel Aromatic Carbocation-Based Coupling Reagent for Esterification and Amidation Reactions. *Chem. Commun.* **2015**, *51* (15), 3131–3134. https://doi.org/10.1039/C4CC09539A.
(59) Langley, C. H.; Lii, J.-H.; Allinger, N. L. Molecular Mechanics Calculations on Carbonyl Compounds. IV. Heats of Formation. *J. Comput. Chem.* **2001**, *22* (13), 1476–1483. https://doi.org/10.1002/jcc.1101.
(60) Verevkin, S. P.; Krasnykh, E. L.; Vasiltsova, T. V.; Koutek, B.; Doubsky, J.; Heintz, A. Vapor Pressures and Enthalpies of Vaporization of a Series of the Linear Aliphatic Aldehydes. *Fluid Phase Equilibria* **2003**, *206* (1–2), 331–339. https://doi.org/10.1016/S0378-3812(03)00035-9.
(61) Chickos, J. S.; Webb, P.; Nichols, G.; Kiyobayashi, T.; Cheng, P.-C.; Scott, L. The Enthalpy of Vaporization and Sublimation of Corannulene, Coronene, and Perylene at T = 298.15 K. *J. Chem. Thermodyn.* **2002**, *34* (8), 1195–1206. https://doi.org/10.1006/jcht.2002.0977.
(62) Saluja, P. P. S.; Peacock, L. A.; Fuchs, R. Enthalpies of Interaction of Aliphatic Ketones with Polar and Nonpolar Solvents. *J. Am. Chem. Soc.* **1979**, *101* (8), 1958–1962. https://doi.org/10.1021/ja00502a005.
(63) Glaser, F.; Rüland, H. Untersuchungen über Dampfdruckkurven und kritische Daten einiger technisch wichtiger organischer Substanzen. *Chem. Ing. Tech.* **1957**, *29* (12), 772–775. https://doi.org/10.1002/cite.330291204.
(64) Chickos, J. S.; Hosseini, S.; Hesse, D. G. Determination of Vaporization Enthalpies of Simple Organic Molecules by Correlations of Changes in Gas Chromatographic Net Retention Times. *Thermochim. Acta* **1995**, *249*, 41–62. https://doi.org/10.1016/0040-6031(95)90670-3.

(65) Benson, S. W.; Buss, J. H. Additivity Rules for the Estimation of Molecular Properties. Thermodynamic Properties. *J. Chem. Phys.* **1958**, *29* (3), 546–572. https://doi.org/10.1063/1.1744539.
(66) Informatics, N. O. of D. and. *NIST Chemistry WebBook*. https://webbook.nist.gov/chemistry/ (accessed 2026-03-10).
